# Single-atom Mo-tailored high-entropy-alloy ultrathin nanosheets with intrinsic tensile strain enhance electrocatalysis

**DOI:** 10.1038/s41467-024-45874-z

**Published:** 2024-03-13

**Authors:** Lin He, Menggang Li, Longyu Qiu, Shuo Geng, Yequn Liu, Fenyang Tian, Mingchuan Luo, Hu Liu, Yongsheng Yu, Weiwei Yang, Shaojun Guo

**Affiliations:** 1 State Key Laboratory of Urban Water Resource and Environment, School of Chemistry and Chemical Engineering, Harbin Institute of Technology, Harbin, Heilongjiang 150001 China; 2 School of Materials Science and Engineering, Peking University, Beijing, 100871 China; 3 Department of Chemical Engineering, School of Chemistry and Chemical Engineering, Guizhou University, Guiyang, 550025 China; 4 Analytical Instrumentation Center, State Key Laboratory of Coal Conversion, Institute of Coal Chemistry, Chinese Academy of Sciences, Taiyuan, Shanxi 030001 China; 5 Key Laboratory of Green and High-end Utilization of Salt Lake Resources, Qinghai Institute of Salt Lakes, Chinese Academy of Sciences, Xining, 810008 China

**Keywords:** Electrocatalysis, Electrocatalysis

## Abstract

The precise structural integration of single-atom and high-entropy-alloy features for energy electrocatalysis is highly appealing for energy conversion, *yet* remains a grand challenge. Herein, we report a class of single-atom Mo-tailored PdPtNiCuZn high-entropy-alloy nanosheets with dilute Pt-Pt ensembles and intrinsic tensile strain (Mo_1_-PdPtNiCuZn) as efficient electrocatalysts for enhancing the methanol oxidation reaction catalysis. The as-made Mo_1_-PdPtNiCuZn delivers an extraordinary mass activity of 24.55 A mg_Pt_^−1^ and 11.62 A mg_Pd+Pt_^−1^, along with impressive long-term durability. The planted oxophilic Mo single atoms as promoters modify the electronic structure of isolated Pt sites in the high-entropy-alloy host, suppressing the formation of CO adsorbates and steering the reaction towards the formate pathway. Meanwhile, Mo promoters and tensile strain synergistically optimize the adsorption behaviour of intermediates to achieve a more energetically favourable pathway and minimize the methanol oxidation reaction barrier. This work advances the design of atomically precise catalytic sites by creating a new paradigm of single atom-tailored high-entropy alloys, opening an encouraging pathway to the design of CO-tolerance electrocatalysts.

## Abstract (web summary)

Atomically precise modification of high-entropy alloys is highly appealing for electrocatalysis. Here, the authors design single-atom Mo-tailored high-entropy alloy nanosheets with dilute Pt-Pt ensembles and intrinsic tensile strain for promoting methanol electro-oxidation towards formate.

## Introduction

Polymer electrolyte membrane fuel cells are considered as the most promising energy conversion devices that can compensate for the increasing global demand for sustainable energy^1–3^. In particular, owing to their environmentally friendly, high energy conversion efficiency and convenient transport, direct methanol fuel cells are highly desirable devices for energy conversion in electric vehicles and portable electronic devices^4–6^. However, the high dosages of Pt-based catalysts (the most common for anodic methanol oxidation reaction (MOR)) and poor CO adsorbates (CO_ads_) (the notorious intermediate blamed for poisoning Pt active sites in MOR) tolerance hinder their commercialization^7,8^. Given the CO-poisoning effect, improving the catalyst efficiency by attenuating the further oxidation barrier of CO* or switching the reaction to a CO-free dominated pathway will be desirable^9^.

Conventional wisdom to address the activity and durability issues of precious-metal-based catalysts mainly includes manipulating architectures, alloying with other transition metals (M), tailoring morphologies, optimizing supports, and so on^10–14^. Although the construction of PtM alloys has been implemented to rationally tune the electronic structure of Pt to optimize the binding energies, the capability of electron modulation is hampered by the restricted compositional scope of M^15,16^. Moreover, the abundance of interlinked Pt-Pt ensembles in PtM alloys makes it more likely to form CO_ads_, with only the Pt sites around the additional metal atoms that can tolerate CO poisoning, making it challenging to maximize the catalytic activity and durability^17,18^.

High entropy alloys (HEAs) are promising materials for the freedom from CO poisoning and improving MOR performance because of their robust capacity for isolating Pt atoms and their expansive and modulable compositional space. Stemming from alloying-induced charge redistribution and enabling multiple active sites, various HEAs have been designed to suitably modulate the binding energies of critical reaction intermediate species toward multi-electron involved MOR electrocatalysis^19,20^. Despite recent important endeavors in the synthesis of HEAs^21–25^, precise design and optimization of HEAs at the atomic level for greatly enhancing the activity, CO tolerance, and stability for MOR is still a grand challenge.

In this study, an oxophilic Mo metal in the form of atomically dispersed sites was used to tailor the HEA nanosheets (NSs) for significantly boosting the MOR electrocatalysis in terms of activity, CO tolerance and durability. The optimized well-defined Mo_1_-PdPtNiCuZn single-atom high-entropy-alloy (SAHEA) NSs delivered an ultrahigh mass activity of 24.55 A mg_Pt_^−1^ and 11.62 A mg_Pd+Pt_^−1^, respectively for MOR, 18.13 and 8.58 folds higher than that of Pt/C catalysts, with the impressive durability. Theoretical calculations and spectroscopic results unveil that the superior MOR performance of Mo_1_-PdPtNiCuZn SAHEA NSs is due to the isolated Mo atoms and intrinsic tensile strain for constructing a suitable electronic microenvironment for the diluted Pt sites, facilitating the deep oxidation of the key reactive species. This switches the intermediates from CO_ads_ to formate that circumvents CO poisoning and accelerates the MOR process kinetically and thermodynamically.

## Results

### Material synthesis and characterizations

**Fig. 1:**
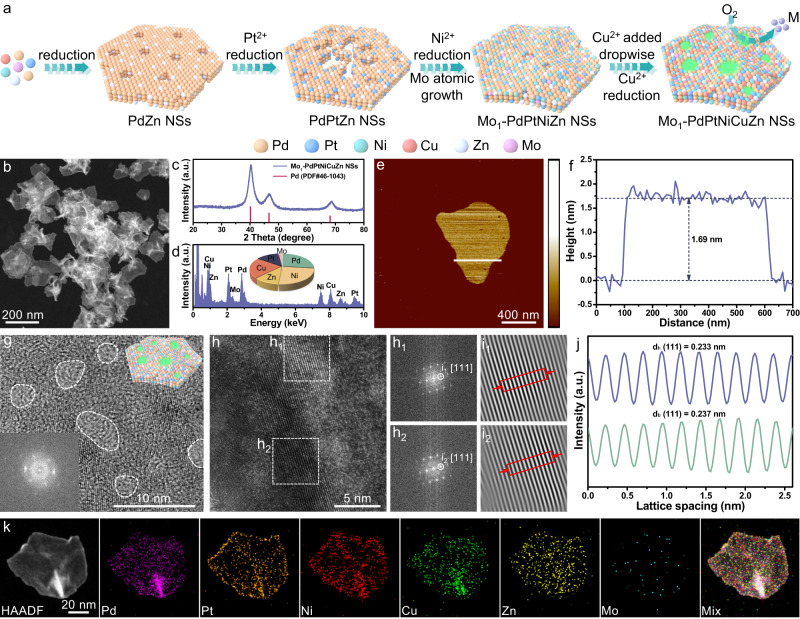
Synthesis and characterizations of Mo_1_-PdPtNiCuZn SAHEA NSs. **a** Schematic illustration of the formation mechanism of Mo_1_-PdPtNiCuZn SAHEA NSs. **b** HAADF-STEM image, (**c**) XRD pattern and (**d**) STEM-EDS spectra (*inset* is the corresponding ICP-OES result) of Mo_1_-PdPtNiCuZn SAHEA NSs. **e** AFM image and (**f**) corresponding height profile of single Mo_1_-PdPtNiCuZn SAHEA NS. **g** HRTEM image (*inset* is the FFT pattern and 3D model) of a single Mo_1_-PdPtNiCuZn SAHEA NS. **h** High-resolution HAADF-STEM image of the surface atomic arrangement on a single Mo_1_-PdPtNiCuZn SAHEA NS and h_1_, h_2_ the corresponding FFT patterns from the dashed white areas in (**h**). i_1_, i_2_ The corresponding inverse FFT patterns of h_1_ and h_2_. **j** The intensity profiles from the red areas in i_1_ and i_2_. **k** HAADF-STEM-EDS elemental mapping of a single Mo_1_-PdPtNiCuZn SAHEA NS.

The immobilization of isolated Mo-atoms on tensile-strained HEA NSs was achieved by a simple one-pot liquid-phase synthesis method (Details in Experimental Section). To understand the growth mechanism, the intermediates collected at different growth stages were investigated using Mo_1_-PdPtNiCuZn SAHEA NSs as a platform (Supplementary Figs. 1–4). Overall, the growth for SAHEA NSs can be divided into three major steps, involving the initial formation of atomic-thick Pd-rich nanosheets, controlled reduction and diffusion of other reducible atoms (e.g., Pt, Ni, Cu, and Zn), and subsequent formation of atomically dispersed Mo sites on the surface of NSs. Figure 1a schematically illustrates the synthetic procedure and shape evolution of SAHEA NSs. In particular, the appropriate dosages of Mo(CO)_6_ and reducing agent (L-ascorbic acid) are critical to forming single-atom Mo-tailored HEA NSs. The XRD patterns of different intermediates all show three typical face-centered cubic (*fcc*) diffraction peaks, which gradually shift to higher angles as the reaction progresses, revealing that more atoms were alloyed with Pd NSs during the post-reaction stages.

Representative transmission electron microscopy (TEM) and low-magnification high-angle annular dark-field scanning TEM (HAADF-STEM) images display that the Mo_1_-PdPtNiCuZn SAHEA NSs are entirely dominated by two-dimensional (2D) graphene-like ultrathin nanosheets (Supplementary Fig. 5 and Fig. 1b). X-ray diffraction (XRD) pattern exhibits a typical *fcc* structure of Pd (PDF# 46-1043) without apparent phase segregation, implying the successful creation of HEAs (Fig. 1c). Meanwhile, the weakening and broadening of diffraction peaks reveal the presence of lattice distortion in Mo_1_-PdPtNiCuZn SAHEA NSs. The atomic ratio of Pd/Pt/Ni/Cu/Zn/Mo in Mo_1_-PdPtNiCuZn SAHEA NSs is determined to be 24.1/14.6/26.3/22.1/11.1/1.8 by inductively coupled plasma mass spectrometry (ICP-MS), in well agreement with the STEM energy-dispersive X-ray spectroscopy (STEM-EDS) and inductively coupled plasma optical emission spectroscopy (ICP-OES) results (Fig. 1d and Supplementary Table 1). The average thickness was determined by atomic force microscopy (AFM) to be about 1.69 nm, further confirming their ultrathin character (Fig. 1e, f). Moreover, the polycrystalline properties and abundant atomic-scale concave defects can be revealed by the high-resolution TEM (HRTEM) image taken from individual Mo_1_-PdPtNiCuZn SAHEA NS, mainly arising from the oxidative etching by trace amount of O_2_ introduced during the addition of Cu precursors (Fig. 1g). This can be confirmed by the fact that only pierced Mo_1_-PdPtNiCuZn SAHEA NSs were obtained when introducing more O_2_ into the Cu precursor solution (Supplementary Fig. 6). The aberration-corrected HAADF-STEM image further reveals the high-crystallinity of Mo_1_-PdPtNiCuZn SAHEA NSs with the clear lattice fringes of (111) facets, which can also be verified by the corresponding fast Fourier transform (FFT) patterns (Fig. 1h–h_2_). Furthermore, the inverse FFT patterns and the corresponding integrated pixel intensities display the average lattice spacing varies from 0.233 nm to 0.237 nm (Fig. 1i, j and Supplementary Fig. 7), indicating substantial lattice distortions and ~4.4% intrinsic tensile strain in the Mo_1_-PdPtNiCuZn SAHEA NSs, arising from the difference in atomic sizes of each component, concave defects, and ultrathin thickness^26^. The elemental EDS mapping demonstrates the homogeneous distribution of Pd, Pt, Ni, Cu, and Zn elements, and isolated Mo atoms in the Mo_1_-PdPtNiCuZn SAHEA NSs (Fig. 1k). In addition, X-ray photoelectron spectroscopy (XPS) analysis was performed to further confirm the presence of Pd, Pt, Ni, Cu, Zn, and Mo in Mo_1_-PdPtNiCuZn SAHEA NSs (Supplementary Fig. 8). Note that Pd, Pt, Ni, Cu, and Zn atoms are mainly in the metallic states, while Mo atoms are primarily in the oxidation states, reflecting the fact that the Mo atoms in Mo_1_-PdPtNiCuZn HEA NSs are under-coordinated atoms and more oxyphilic than other atoms on facets. (Supplementary Fig. 9). Given the ease with which Mo is oxidized, we deduce that the Mo atoms are located on the surface of Mo_1_-PdPtNiCuZn SAHEA NSs^27^.

**Fig. 2:**
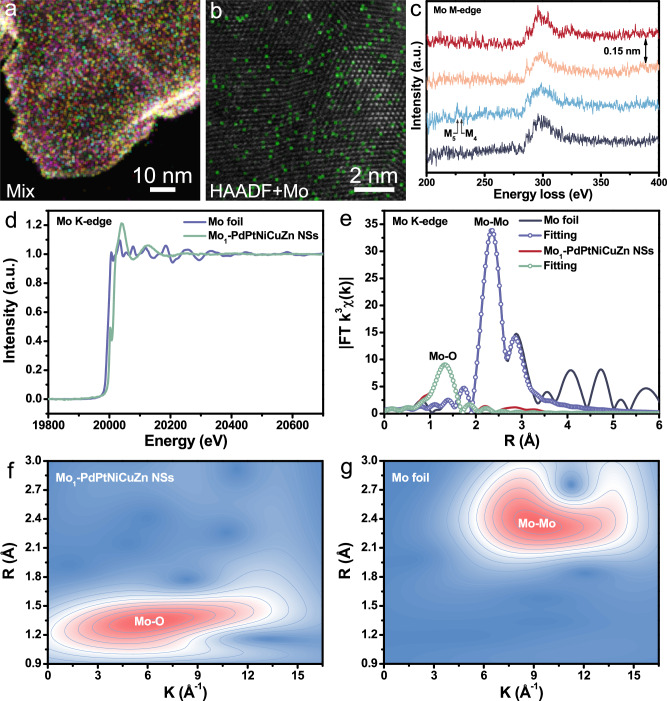
Compositional and electro-structural characterizations of Mo_1_-PdPtNiCuZn SAHEA NSs. **a**, **b** High-resolution EDX elemental mapping. **c** Background-subtracted EELS line-scanning spectra of four adjacent atomic columns in Mo_1_-PdPtNiCuZn SAHEA NSs acquired along the line in Supplementary Fig. 10 (the M_4,5_ edges for Mo are indicated). **d** Mo K-edge XANES spectra of Mo_1_-PdPtNiCuZn SAHEA NSs and Mo foil. **e** The *k*^3^-weighted Fourier transformation of the Mo K-edge EXAFS spectra. Wavelet transformation of the *k*^3^-weighted Mo K-edge EXAFS signals of (**f**) Mo_1_-PdPtNiCuZn SAHEA NSs and (**g**) Mo foil.

High-resolution EDX elemental mapping demonstrates that Pd, Pt, Ni, Cu, and Zn elements are uniformly distributed throughout the Mo_1_-PdPtNiCuZn SAHEA NSs (Fig. 2a). However, Mo atoms are distributed sporadically but evenly doped into the NSs in the form of an isolated atom, providing the preliminary evidence for the isolated Mo single-atoms on Mo_1_-PdPtNiCuZn SAHEA NSs (Fig. 2b). Atomic-level electron energy loss spectroscopy (EELS) line-scanning acquisition was further performed to confirm that Mo atoms existed as single atoms (Fig. 2c and Supplementary Fig. 10). The spectra demonstrate the presence of a Mo atom in these atomic columns, with the signature M_4,5_ edge occurring at the expected energy levels^28^. These features are not present in the EELS of other atomic positions adjacent to this atomic site, proving that the Mo species in Mo_1_-PdPtNiCuZn SAHEA NSs mainly exist as single atoms. The coordination environment and valence state of Mo atoms in Mo_1_-PdPtNiCuZn SAHEA NSs were further confirmed by the X-ray absorption fine structure (XAFS) measurements. The X-ray absorption near-edge structure (XANES) spectra show that the Mo K-edge adsorption threshold position and white line intensity of Mo_1_-PdPtNiCuZn SAHEA NSs are higher than those of Mo foil, implying that the surface-exposed Mo atoms are in high valence states, consistent with XPS results (Fig. 2d). Notably, the XANES spectrum of Mo_1_-PdPtNiCuZn SAHEA NSs indicates a shoulder around 20010 eV in the pre-edge region, indicating that the existence of MoO_*x*_ with distorted [MoO_6_] octahedron structure^29,30^. In order to further clarify the subtle information about coordination circumstance, the *k*3-weight Fourier transforms of extended X-ray absorption fine structure (FT-EXAFS) curves of Mo_1_-PdPtNiCuZn SAHEA NSs and Mo foil at Mo K-edge were fitted in R and k spaces (Fig. 2e and Supplementary Fig. 11). The corresponding fitting parameters can be inspected in Supplementary Table 2. The *k*^3^-weight FT-EXAFS spectra reveal that the Mo_1_-PdPtNiCuZn SAHEA NSs exhibit the major peak at ~ 1.33 Å, attributed to Mo-O scattering from MoO_*x*_ in SAHEAs. The characteristic peak of Mo-Mo scattering (~2.34 Å for the Mo foil) is almost absent, strongly confirming that the Mo species in Mo_1_-PdPtNiCuZn SAHEA NSs mainly exist in the form of atomically dispersed sites without long-range coordination to other Mo metal centres, further implying that the Mo species act as surface-modified species^31,32^. Furthermore, the wavelet transform (WT) EXAFS reconfirms macroscopically Mo exhibits the feature of isolated metal atoms (Fig. 2f, g). Taken cumulatively, the above results demonstrate the successful creation of single-atom Mo-tailored PdPtNiCuZn HEA NSs catalysts.

**Fig. 3:**
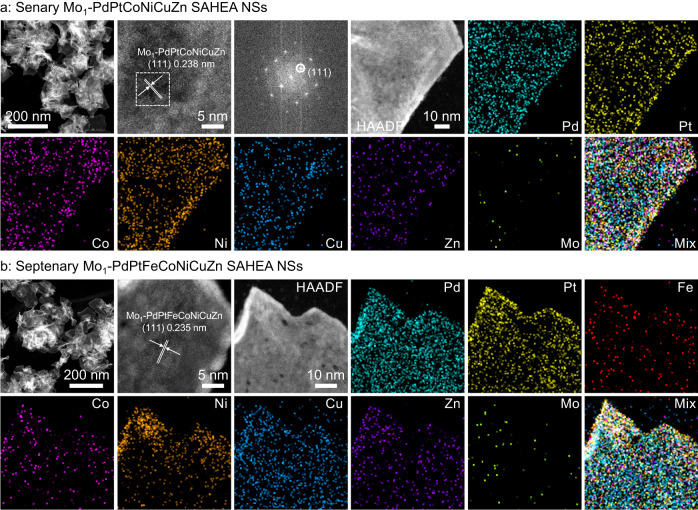
Generality for synthesis of SAHEA NSs. **a** HAADF-STEM image, HAADF-STEM-EDS elemental mapping, and high-resolution HAADF-STEM image of senary Mo_1_-PdPtCoNiCuZn SAHEA NSs with the corresponding FFT pattern taken from the white dashed area in (**a**). **b** HAADF-STEM image, high-resolution HAADF-STEM image, and HAADF-STEM-EDS elemental mapping of septenary Mo_1_-PdPtFeCoNiCuZn SAHEA NSs.

Considering that other reducible elements (e.g., Fe, Co, Mn) can be alloyed with Pt in the presence of reductant in the OAm system at 200 °C^16^, the present synthetic method for single-atom Mo-tailored HEA NSs with tensile-strained allows for the expansion of the compositional space of HEA hosts, such as senary Mo_1_-PdPtCoNiCuZn SAHEA NSs (Fig. 3a and Supplementary Fig. 12) and septenary Mo_1_-PdPtFeCoNiCuZn SAHEA NSs (Fig. 3b and Supplementary Fig. 13), etc. Remarkably, high-resolution EDX elemental mapping demonstrates the uniform distribution of Pd, Pt, (Fe), Co, Ni, Cu, and Zn elements and the isolated Mo atoms in these SAHEA NSs. The *k*^3^-weight FT-EXAFS spectra further verify the single-atom feature of Mo species (Supplementary Fig. 14). Meanwhile, conspicuous intrinsic tensile strain, lattice deformation, and defects were observed in all the SAHEA NSs mentioned above, mainly generated by the ultrathin feature and significant differences in the sizes of various atoms. These results confirm the generality of the present synthetic method for single-atom Mo-tailored SAHEA NSs. Besides, we found that the subsequent addition of Cu(acac)_2_ was the key to forming Mo_1_-PdPtNiCuZn SAHEA NSs. The Mo_1_-PdPtNiCuZn SAHEA nanoparticle assemblies (NPs) can be obtained by the simultaneous introductions of Cu(acac)_2_ and other metal precursors before the reaction, while other conditions are kept unchanged. As-obtained NPs exhibit compositions and single-atom Mo-tailored structures similar to SAHEA NSs (Supplementary Figs. 15, 16).

### Electrocatalytic performance tests toward MOR

**Fig. 4:**
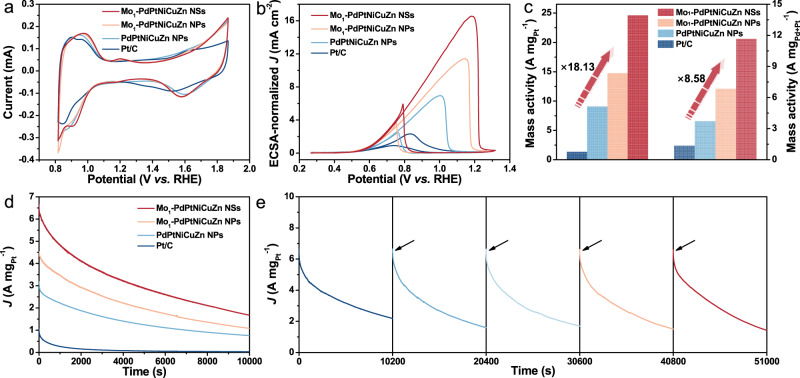
MOR performances of different catalysts in alkaline electrolytes. **a** CVs of Mo_1_-PdPtNiCuZn SAHEA NSs, Mo_1_-PdPtNiCuZn SAHEA NPs, PdPtNiCuZn HEA NPs, and Pt/C in N_2_-saturated 0.1 M HClO4. **b** ECSA-normalized MOR curves. **c** MOR mass activity of Mo_1_-PdPtNiCuZn SAHEA NSs, Mo_1_-PdPtNiCuZn SAHEA NPs, PdPtNiCuZn HEA NPs, and Pt/C in 1.0 M KOH containing 1.0 M methanol. **d** Chronoamperometric tests for MOR at 0.77 V *vs*. RHE of Mo_1_-PdPtNiCuZn SAHEA NSs, Mo_1_-PdPtNiCuZn SAHEA NPs, PdPtNiCuZn HEA NPs and Pt/C. **e** Long-term durability of Mo_1_-PdPtNiCuZn SAHEA NSs, and the arrows indicate when the electrolyte is refreshed.

To validate the potential application of these customized single-atom Mo-modified HEA catalysts in MOR, the MOR performances were evaluated in a typical three-electrode system in 1.0 M KOH + 1.0 M methanol. Before electrocatalytic tests, Mo_1_-PdPtNiCuZn SAHEA NSs, Mo_1_-PdPtNiCuZn SAHEA NPs, and PdPtNiCuZn HEA NPs were deposited onto commercial carbon support through continuous ultrasonication, and washed with ethanol to ensure the surface of catalysts be clean (Supplementary Fig. 17). The observation of typical H atom adsorption/desorption and the oxidation/reduction of Pt regions indicates the high Pt atom utilization of these catalysts (Fig. 4a). Notably, the oxidation peaks of Mo species (~1.21 V) can be obviously identified in the amplified cyclic voltammograms (CV) curves of the Mo_1_-PdPtNiCuZn SAHEA NSs and Mo_1_-PdPtNiCuZn SAHEA NPs, further demonstrating the presence of Mo atoms (Supplementary Fig. 18). The electrochemically active surface areas (ECSAs) are determined to be 70.21, 59.89, 53.32, and 58.29 m^2^ g^−1^ for Mo_1_-PdPtNiCuZn SAHEA NSs, Mo_1_-PdPtNiCuZn SAHEA NPs, PdPtNiCuZn HEA NPs, and Pt/C, respectively by the CV. The largest ECSA of Mo_1_-PdPtNiCuZn SAHEA NSs can be attributed to its ultrathin 2D characters, revealing the ultrahigh utilization of noble metals^27^. In 1.0 KOH + 1.0 M methanol, all catalysts display a clear anodic peak during the forward and reverse scanning, corresponding to the oxidation process of methanol and the intermediates (Fig. 4b and Supplementary Fig. 19). The Mo_1_-PdPtNiCuZn SAHEA NSs, Mo_1_-PdPtNiCuZn SAHEA NPs, and PdPtNiCuZn HEA NPs all show much higher MOR activities than commercial Pt/C catalysts. Planting the isolated Mo single atoms in high oxidation states as promoters into PdPtNiCuZn HEA NPs, the as-obtained Mo_1_-PdPtNiCuZn SAHEA NPs show higher activity. Moreover, the constructed Mo_1_-PdPtNiCuZn SAHEA NSs with intrinsic tensile strain deliver the highest MOR specific activity of 16.55 mA cm^−2^, obviously higher than those of Mo_1_-PdPtNiCuZn SAHEA NPs and most of reported Pd/Pt-based electrocatalysts, and also 7.13 times higher than that of commercial Pt/C (Supplementary Table 3). In addition, the mass activities of Mo_1_-PdPtNiCuZn SAHEA NSs can be calculated to be 24.55 A mg_Pt_^−1^ and 11.62 A mg_Pd+Pt_^−1^, 18.13/8.58 times higher than those of commercial Pt/C (Fig. 4c). Furthermore, the charge transfer kinetics of Mo_1_-PdPtNiCuZn SAHEA NSs, Mo_1_-PdPtNiCuZn SAHEA NPs, PdPtNiCuZn HEA NPs and Pt/C towards MOR were investigated by electrochemical impedance spectroscopy (EIS). Among the four catalysts, Mo_1_-PdPtNiCuZn SAHEA NSs represent the minimum electrochemical impedance under 0.75 V *vs*. RHE, reflecting the maximum charge conductivity (Supplementary Fig. 20). The above results suggest that tailoring the PdPtNiCuZn high-entropy alloy host with atomically dispersed Mo single atoms allows for significant modulation of the adsorption behavior of the key intermediates involved MOR, resulting in an optimal reaction path and high performance. Meanwhile, tensile strain can also assist the adsorption/desorption process, synergistically contributing to efficient MOR catalysis^27,33^. The sharp anodic peaks of Mo_1_-PdPtNiCuZn SAHEA NSs and Mo_1_-PdPtNiCuZn SAHEA NPs in the reverse scan further confirm that the electronic effect via dispersing Mo as single atoms can be utilized to enhance the reaction selectivity and kinetics, thus obtaining higher catalytic performance.

To evaluate the MOR stability, the chronoamperometry (CA) measurements of the Mo_1_-PdPtNiCuZn SAHEA NSs, Mo_1_-PdPtNiCuZn SAHEA NPs, PdPtNiCuZn HEA NPs, and commercial Pt/C catalysts were executed at 0.77 V *vs*. RHE for 10,000 s. As shown in Fig. 4d, the Mo_1_-PdPtNiCuZn SAHEA NSs, Mo_1_-PdPtNiCuZn SAHEA NPs, and PdPtNiCuZn HEA NPs all display much higher current densities than that of commercial Pt/C, indicating their excellent long-term durability toward MOR. Additionally, CA tests of the Mo_1_-PdPtNiCuZn SAHEA NSs for MOR have been further prolonged to 10 h (Supplementary Fig. 21). The current densities remain 1.15 A mg_Pt_^−1^ for MOR after the long-term durability tests, further confirming the superior stability of Mo_1_-PdPtNiCuZn SAHEA NSs. Of note, the catalytic activity of Mo_1_-PdPtNiCuZn SAHEA NSs can be restored by replacing fresh working electrolytes after five consecutive cycles of CA tests, revealing respectable reproducibility (Fig. 4e). The morphologies of Mo_1_-PdPtNiCuZn SAHEA NSs, Mo_1_-PdPtNiCuZn SAHEA NPs, and PdPtNiCuZn HEA NPs after stability tests can be well preserved, while the commercial Pt/C exhibits obvious aggregation (Supplementary Fig. 22). The 2D ultrathin nanosheet shape and multimetallic compositions of Mo_1_-PdPtNiCuZn SAHEA NSs are maintained, further verifying the impressive compositional and structural stability under the applied potential of 0.77 V (Supplementary Fig. 23). XPS spectra of Mo_1_-PdPtNiCuZn SAHEA NSs after MOR tests were very similar to that before MOR electrocatalysis, further confirming excellent stability (Supplementary Figs. 24, 25).

### Mechanism investigation

**Fig. 5:**
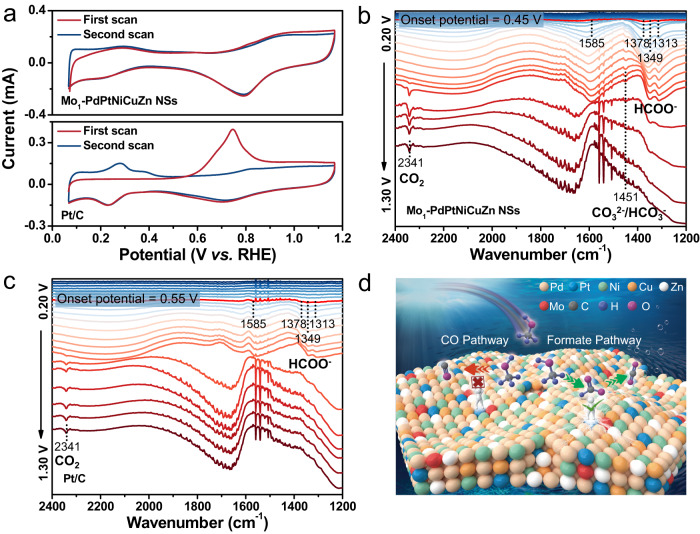
Reaction mechanism of MOR on Mo_1_-PdPtNiCuZn SAHEA NSs. **a** CO-stripping curves of Mo_1_-PdPtNiCuZn SAHEA NSs and Pt/C catalysts recorded in N_2_-saturated 1.0 M KOH. In-situ FTIR spectrum of MOR on (**b**) Mo_1_-PdPtNiCuZn SAHEA NSs/C and (**c**) Pt/C at different potentials varying from 0.20 to 1.30 V at an interval of 0.05 V in 1.0 M KOH + 1.0 M methanol solution. **d** Schematic diagram of possible pathways for MOR on Mo_1_-PdPtNiCuZn SAHEA NSs.

To gain insight into the origin of the high MOR performance of Mo_1_-PdPtNiCuZn SAHEA NSs, we employed CO stripping and in-situ spectroscopy tests. As shown in Fig. 5a, the oxidation peak of the absorbed CO at around 0.75 V *vs*. RHE is more repressed on the Mo_1_-PdPtNiCuZn SAHEA NSs compared to that of commercial Pt/C, indicating the remarkable tolerance to CO poisoning. This is because the multiple metals in the Mo_1_-PdPtNiCuZn SAHEA NSs can significantly dilute the Pt-Pt sites for forming the isolated Pt sites on the surface, and the tensile strain expands the Pt-Pt distance. These structural characteristics favor the switch from MOR to a CO-free dominated pathway due to the formation of CO_ads_ requiring the presence of at least three contiguous Pt atoms or defect sites^20^. In addition, the exposure and modification of oxophilic Mo single-atoms can effectively tune the *d*-band center of their adjacent sites, achieving a favorable balance between effective dissociation of reactants and proper binding of intermediates for improving MOR selectivity and accelerating reaction kinetics. Therefore, the Mo_1_-PdPtNiCuZn SAHEA NSs achieve double enhancement of catalytic activity and stability by avoiding the formation of CO_ads_ in MOR, boosting the deep oxidation of key reaction intermediate, and accelerating the kinetics of the rate-determining step. Moreover, the enhanced MOR electrocatalytic performance caused by the diluted Pt-Pt ensembles can be further confirmed by the fact that the Mo_1_-PdPtNiCuZn SAHEA NSs catalysts exhibit significantly enhanced MOR performances over the single-atom Mo-tailored low- and medium-entropy-alloy NSs (Supplementary Figs. 26, 27). Specifically, the construction of Mo-tailored SAHEA NSs can significantly dilute Pt-Pt ensembles to achieve the slightest formation of CO_ads_ and optimize electronic structures to enhance the formate pathway (Supplementary Fig. 28).

In-situ Fourier transform infrared (FTIR) spectroscopy was performed to track the reaction intermediate during MOR electrocatalysis. Several characteristic bands between 2400 and 1200 cm^−1^ can be observed clearly in the investigated potential window (Fig. 5b, c). Specifically, the bands at ca. 1585, 1378, and 1349 cm^−1^ can be attributed to *v*_as_ (OCO), *δ* (C-H), and *v*_s_ (OCO) of formate (HCOO^−^), respectively. The downward band located at about 1313 cm^−1^ belongs to another characteristic peak of formate whose specific vibrational mode has not been confirmed *yet*. Notably, there is no CO_L_ signal over the entire potential range, whereas CO_2_ (ca. 2341 cm^−1^) appears at high potentials, manifesting that CO_2_ originates from further oxidation of the weakly adsorbed formate^34^. Moreover, the band at about 1451 cm^−1^ belongs to the *v*_as_ of CO_3_^2-^/HCO_3_^−^, formed by the reaction of desorbed CO_2_ with OH^−^. The onset potential for producing the formate (characteristic bands of 1585, 1378, 1349, and 1313 cm^−1^) over Mo_1_-PdPtNiCuZn SAHEA NSs is 0.45 V, 100 mV lower than that of commercial Pt/C (0.55 V). The much stronger formate characteristic band (*v*_s_ (OCO) of formate) on Mo_1_-PdPtNiCuZn SAHEA NSs indicates that methanol can be more easily oxidized to formate on Mo_1_-PdPtNiCuZn SAHEA NSs (Supplementary Fig. 29). Meanwhile, more CO_2_ is observed on Mo_1_-PdPtNiCuZn SAHEA NSs, meaning that methanol is more readily oxidized to formate and further to CO_2_, further supporting the outstanding MOR performances on Mo_1_-PdPtNiCuZn SAHEA NSs. Combining CO stripping and in-situ FTIR studies, the Mo_1_-PdPtNiCuZn SAHEA NSs can avoid the formation of CO_ads_ and switch the MOR to the formate-dominated pathway (Fig. 5d), while the MOR process of Pt/C involves both the CO pathway and the formate pathway, resulting in the catalyst deactivation.

**Fig. 6:**
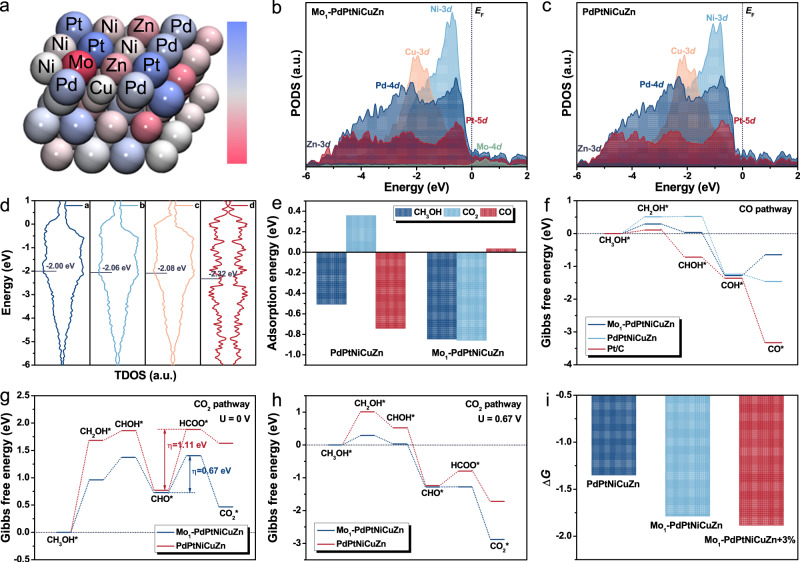
DFT calculations for the structural configuration and energetic reaction pathways. **a** The Bader charge simulation for Mo_1_-PtPdFeCoNi. The PDOSs of (**b**) Mo_1_-PdPtNiCuZn and **c** PdPtNiCuZn. **d** The TDOS of *d*-band in (**a**) strained Mo_1_-PdPtNiCuZn, (**b**) Mo_1_-PdPtNiCuZn, (**c**) PdPtNiCuZn, and (**d**) Pt/C models. **e** The adsorption energy comparison of CH_3_OH, CO_2_, and CO on Mo_1_-PdPtNiCuZn and PdPtNiCuZn. The reaction energy comparison of (**f**) CO pathway and (**g**, **h**) CO_2_ pathway for MOR electrocatalysis on Mo_1_-PdPtNiCuZn, PdPtNiCuZn, and Pt/C. **i** The ∆*G* of RDS on strained Mo_1_-PdPtNiCuZn, Mo_1_-PdPtNiCuZn, and PdPtNiCuZn.

Density functional theory (DFT) calculations were conducted to further verify the mechanism for the enhanced MOR performance over Mo_1_-PdPtNiCuZn SAHEA NSs. Considering the incorporation of Mo single atoms and the introduction of tensile strain, the strained Mo_1_-PdPtNiCuZn, Mo_1_-PdPtNiCuZn, and PdPtNiCuZn surfaces were constructed as calculation models (Supplementary Fig. 30). Bader charge simulation reveals that the charge state of Pt atoms becomes more negative with high electron density, indicating that the electrons are more localized around the Pt atoms. However, the Mo atoms are electron deficient, meaning the efficient electron transfer, in agreement with the XPS analysis (Fig. 6a and Supplementary Fig. 31). The results illustrate that introducing single-atom Mo can efficaciously optimize the electronic density around Pt sites. To further understand the electronic structures of Mo_1_-PdPtNiCuZn SAHEA and PdPtNiCuZn HEA, the partial projected density of states (PDOSs) of each element in HEA has been illustrated. As depicted in Fig. 6b, c, the Ni and Mo elements serve as electron depletion centers during the MOR process, promoting electron transfer for the HEA surfaces. Meanwhile, the efficient *d*-*d* orbital coupling between Pt, Pd, Cu, and Zn not only lowers the energy barrier of electron transfer during oxidation but also can stabilize the valence state of Pt sites, facilitating the stabilization of intermediates for MOR^19^. Such element combination can provide the optimal surface configuration, suitable electronic microenvironment, and ideal adsorption/desorption sites for MOR, enabling proper adsorption of key intermediates to enhance the formate-dominated MOR. Compared with the PdPtNiCuZn, introducing Mo single atoms near the Pt sites further enhances the electroactivity. Moreover, the total *d*-density of states (TDOS) plots of different models were calculated to visualize the *d*-band centers, whose positions can be directly associated with enhanced MOR activity (Fig. 6d). Comparison with the electronic structure of PdPtNiCuZn reveals that the overall *d*-band center of strained Mo_1_-PdPtNiCuZn has significantly shifted upward after introducing Mo single atoms and tensile strain, further suggesting that the improved electron transfer efficiency of the electrocatalysts. Due to the reduced occupancy of the anti-bonding orbitals, the upward shift of the *d*-band center can contribute to the strong adsorption of intermediates^35^. Therefore, strained Mo_1_-PdPtNiCuZn with the up-shifted *d*-band center displays the stronger binding energy with key reaction intermediates and *OH, thus increasing the chance of these intermediates being further oxidized to CO_2_.

Furthermore, the performances of Mo_1_-PdPtNiCuZn and PdPtNiCuZn are compared from the energetic perspective. As shown in Fig. 6e, the more powerful adsorption of CH_3_OH and CO_2_ on single-atom Mo-modified PdPtNiCuZn leads to enhanced electroactivity toward MOR. As expected, the Mo_1_-PdPtNiCuZn exhibits an unpreferred adsorption CO. We deduce that CO molecules barely adsorb on Mo_1_-PdPtNiCuZn SAHEA NSs in alkaline electrolytes. CO_ads_ may not be a preferable intermediate, which can be further demonstrated by the weaker preference of Mo_1_-PdPtNiCuZn for the CO pathway (Fig. 6f). These calculations suggested that the formation of HEA significantly diluted the continuous Pt-Pt sites, and the planting of Mo single atoms as promoters effectively adjusted the electronic structure of HEA hosts, kinetically and thermodynamically prohibiting the formation of CO_ads_, thus circumventing the CO-poisoning effect and synchronously switching the reaction to the formate dominated pathway. For the CO_2_ pathway, the rate-determining step (RDS) occurs at [CHO* + 3*OH + 3H_2_O] to [HCOO* + *OH + 5H_2_O]^36^. At a potential of U = 0 V, Mo_1_-PdPtNiCuZn exhibits a more energetically favorable pathway and lower overpotential than PdPtNiCuZn (Fig. 6g). At U = 0.67 V, the minimum applied voltage enabling to reach an ideal reaction equilibrium state at the RDS of CO_2_ pathway without extra external energy on Mo_1_-PdPtNiCuZn (111) surface^37^, PdPtNiCuZn shows an uphill slope at the RDS, indicating that the reaction step is an endothermic reaction at this voltage and requires additional energy. In contrast, the ideal reaction equilibrium state is reached at the RDS without extra external energy on Mo_1_-PdPtNiCuZn. The more negative reaction energies further reveal that deep CHO* and HCOO* oxidation on Mo_1_-PdPtNiCuZn becomes more feasible (Fig. 6h). In addition, the influence of tensile strength on reaction tendency at the RDS was also studied (Fig. 6i). The ∆*G* of the RDS on strained Mo_1_-PdPtNiCuZn shows a more negative value due to the combination of modification of Mo single atoms and intrinsic tensile strain, revealing the acceleration in kinetics and thermodynamics consistent with the experimental observations.

## Discussion

In summary, we report a general method for synthesizing a class of single-atom Mo-tailored HEA ultrathin NSs with intrinsic tensile strain (including but not limited to quinary Mo_1_-PdPtNiCuZn, senary Mo_1_-PdPtCoNiCuZn, and septenary Mo_1_-PdPtFeCoNiCuZn SAHEA NSs) as high-performance electrocatalysts toward MOR in alkaline conditions. The representative Mo_1_-PdPtNiCuZn SAHEA NSs exhibit an extraordinary mass activity of 24.55 A mg_Pt_^−1^ and 11.62 A mg_Pd+Pt_^−1^ at the peak potential, respectively, 18.13 and 8.58 times higher than commercial Pt/C catalysts, as well as display impressive durability. In-situ FTIR analysis and DFT calculations unveil that planting oxophilic Mo single atoms can provide a suitable electronic microenvironment for adjacent dilute Pt sites, selectively switch MOR to the formate-dominated pathway, and combine with tensile strain to optimize intermediate adsorption behavior, which logically manipulated the excellent activity and long-term stability. This work demonstrates an effective and practical strategy for the design of engineering-strained single atom-modified HEA catalysts to facilitate the exploration of advanced catalysts with superior catalytic performance for a wide range of applications.

## Methods

### Chemicals

Palladium (II) acetylacetonate (Pd(acac)_2_, 99%), iron (III) acetylacetonate (Fe(acac)_3_, 98%), nickel (II) acetylacetonate (Ni(acac)_2_, 95%), zinc (II) acetylacetonate (Zn(acac)_2_, 97%), cobalt (III) acetylacetonate (Co(acac)_3_, 98%), copper (II) acetylacetonate (Cu(acac)_2_, 97%), molybdenum hexacarbonyl (Mo(CO)_6_, 98%), potassium hydroxide (KOH, 95%) and oleylamine (C_18_H_35_NH_2_, OAm, 80%) were all purchased from Aladdin. Platinum (II) acetylacetonate (Pt(acac)_2_, 97%) was provided by Innochem. L-ascorbic acid (AA, 98%) and Nafion (5 wt.%) were obtained from Adamas. Isopropanol (C_3_H_8_O, GR.), toluene (C_7_H_8_, AR.), methanol (CH_3_OH, AR.), ethanol (C_2_H_5_OH, AR.), and cyclohexane (C_6_H_12_O_6_, AR.) were supplied by Sinopharm Chemical Reagent Co. Ltd. (Shanghai, China). Commercial carbon supported Pt catalyst (Pt/C, 20 wt.% of 3 nm-Pt nanoparticles on carbon black) was provided by Johnson-Matthey Corp. All regents were used without further purification, and all solutions were freshly prepared with ultrapure water (18.2 MΩ cm^−1^).

### Preparation of SAHEA ultrathin NSs

In a typical preparation of quinary Mo_1_-PdPtNiCuZn SAHEA NSs, Pt(acac)_2_ (7.9 mg), Pd(acac)_2_ (12.2 mg), Ni(acac)_2_ (10.3 mg) and Zn(acac)_2_ (10.6 mg) were added into a four-neck flask containing 12 mL of OAm. This mixture was then heated to 80 °C in vacuum and maintained for 15 min under magnetic stirring to remove air and water. Afterward, the flask was purged with nitrogen (N_2_) and cooled to room temperature. After Mo(CO)_6_ (20.0 mg) and AA (30.0 mg) were added into the mixture, the flask was evacuated again for 10 min. Then, the flask was filled with N_2_ and heated to 200 °C under magnetic stirring. Cu(acac)_2_ (7.8 mg) dissolved in 0.5 mL OAm and 0.5 mL toluene was then added dropwise to the above mixture under magnetic stirring. After the injection, the mixed solution was heated at 200 °C for 2 h. At last, the reaction mixture was rapidly cooled down to room temperature and collected by centrifugation. Finally, the products were washed two or three times with a cyclohexane/ethanol mixture.

The preparation of binary Mo_1_-PdPt single-atom low-entropy-alloy (SALEA) NSs was similar to that of quinary Mo_1_-PdPtNiCuZn SAHEA NSs except for the absence of Ni(acac)_2_, Cu(acac)_2_, and Zn(acac)_2_ in the reactants. The preparation of ternary Mo_1_-PdPtNi single-atom medium-entropy-alloy (SAMEA) NSs was similar to that of quinary Mo_1_-PdPtNiCuZn SAHEA NSs except for the absence of Cu(acac)_2_ and Zn(acac)_2_ in the reactants. The preparation of senary Mo_1_-PdPtCoNiCuZn SAHEA NSs was similar to that of quinary Mo_1_-PdPtNiCuZn SAHEA NSs except for adding Co(acac)_2_ (10.3 mg) into reactants. The preparation of septenary Mo_1_-PdPtFeCoNiCuZn SAHEA NSs was similar to that of quinary Mo_1_-PdPtNiCuZn SAHEA NSs except for adding Fe(acac)_3_ (14.1 mg) and Co(acac)_2_ (10.3 mg) into reactants.

### Preparation of Mo_1_-PdPtNiCuZn SAHEA NPs

The synthesis for Mo_1_-PdPtNiCuZn SAHEA NPs was similar to those of Mo_1_-PdPtNiCuZn SAHEA NSs except that the Cu precursor was added into the reaction solution at the beginning.

### Preparation of PdPtNiCuZn HEA NPs

The synthesis for PdPtNiCuZn HEA NPs was similar to those of Mo_1_-PdPtNiCuZn SAHEA NPs except for the absence of Mo(CO)_6_ in the reactants.

### Characterizations

The Transmission electron microscopy (TEM) characterization was collected by JEM-1400 operating at 100 kV (JEOL Ltd). High-resolution TEM (HRTEM), high-angle annular dark-field scanning TEM (HAADF-STEM) and HAADF-STEM energy dispersive X-ray spectroscopy (HAADF-STEM-EDS) were characterized by an FEI Tecnai-G2 F30 at an accelerating voltage of 300 KV. X-ray diffraction spectroscopy (XRD) was performed on a PANanalytical X’Pert Powder with Cu Kα (*λ* = 1.5418 Å). The compositions were analyzed by inductively coupled plasma optical emission spectroscopy (ICP-OES, Optima 8300) and inductively coupled plasma mass spectrometry (ICP-MS, Agilent 7800). X-ray photoelectron spectroscopy (XPS) spectra were recorded on Thermo Scientific (ESCALAB 250 XI). AFM image of the sample was conducted on tapping mode with a Multimode Nanoscope IIIa SPA (Veeco Instruments, Bruker) under ambient conditions. XAS experiments were conducted at the Mo K-edge on the beamline BL1W1B in the Beijing Synchrotron Radiation Facility (BSRF). XAS data were processed and analyzed using the Demeter software package. A linear function was subtracted from the pre-edge region, then the edge jump was normalized using Athena software^38^.

### Electrocatalytic measurements

Before the electrocatalytic measurements, as-synthesized Mo_1_-PdPtNiCuZn SAHEA NSs, Mo_1_-PdPtNiCuZn SAHEA NPs and PdPtNiCuZn HEA NPs were deposited onto the commercial carbon supports (Ketjen Black-300) by sonication for 3 h. The final products were collected via centrifugation and washed with ethanol for three times to obtain the tested carbon-supported catalysts. Subsequently, 1.0 mg of dry catalysts were dispersed in a mixed solvent containing 0.4 mL isopropanol, 0.6 mL ultrapure water, and 10 μL Nafion by ultrasound for 30 min to obtain homogeneous catalyst inks. For the electrochemical tests, the 20 µL of ink was dropped onto a clean glassy-carbon electrode (GCE, diameter: 4 mm) to prepare the working electrode, and the saturated calomel electrode (SCE) and Pt foil (1 cm × 1 cm) were served as reference and counter electrodes, respectively. All the electrochemical measurements were performed on a CHI 660E (Chenhua, Shanghai) electrochemical workstation with a typical three-electrode configuration, and all the recorded potentials were converted to the reversible hydrogen electrode (RHE). The cyclic voltammograms (CVs) were collected in N_2_-saturated 0.1 M HClO_4_ with a scan rate of 50 mV s^−1^. The electrochemical active surface areas (ECSAs) were estimated according to the underpotentially deposited H (H_upd_) methods. From the charge of Hupd desorption peak in the recorded CVs, the ECSAs of catalysts were determined from one monolayer of hydrogen desorption on Pt with a criterion of 0.21 mC cm^−2^.

The methanol oxidation reaction (MOR) polarization curves were obtained at the scan rate of 50 mV s^−1^ in N_2_-saturated 1.0 M KOH + 1.0 M methanol solution. Before MOR tests, the catalysts were activated by conducting the CVs to be stable in N_2_-saturated 1 M KOH at a sweep rate of 500 mV s^−1^. The chronoamperometry (CA) measurements of MOR were conducted in 1.0 M KOH + 1.0 M methanol solution at 0.77 V *vs*. RHE. For the CO stripping measurement, the working electrode was held at a constant potential of −0.88 V *vs*. SCE under a flow of CO bubbled into the N_2_-saturated 1.0 M KOH electrolyte for 15 min. Afterward, the working electrode was quickly moved into a fresh N_2_-saturated 1.0 M KOH electrolyte and recorded the two cycles at a scan rate of 50 mV s^−1^.

### Electrochemical in-situ FTIR reflection spectroscopy

In-situ FTIR was carried out to trace the signals of the intermediates using a Nicolet iS50 Spectroscopy equipped with a liquid nitrogen-cooled mercury-cadmium-telluride (MCT) detector. An ECIR-II cell equipped with a Pike Veemax III ATR in a three-electrode system was provided from Shanghai Linglu Instrument & Equipment Co. 10 µL of catalyst ink was dropped onto glass carbon electrode equipped with specially-made three-electrode thin-layer IR cell configuration, using CaF_2_ as the window. The in-situ FTIR spectroscopy was collected from 0.2 V to 1.3 V (*vs*. RHE) every 50 mV in N_2_-saturated 1.0 M KOH + 1.0 M methanol.

### Calculation setup

The First-Principles calculations were implemented in the Vienna ab initio Simulation Package (VASP) with the Projector-Augmented-Wave (PAW) pseudopotentials within the density functional theory (DFT) framework^39,40^. The electron exchange-correlation energy was described by the Perdew-Burke-Ernzerhof (PBE) functional within the generalized gradient approximation (GGA)^41^. The special quasi-random structure (SQS) of high-entropy alloys was generated using the Monte Carlo SQS (MCSQS) tool within the Alloy Theory Automation Toolkit (ATAT)^42^. An *fcc* cell consisting of 32 atoms was constructed to perform the calculations. The cut-off energy of the plane-wave basis was set to 500 eV. The Brillouin zone integration was sampled using a Monkhorst-Pack (4 × 4 × 1) k-point mesh. Spin polarization was considered in all calculations. The top two layers of atoms in the model were allowed to be adjusted until the residual forces per atom were less than 0.02 eV Å^−1^, and the maximum energy difference was less than 10^−5^ eV.

### Supplementary information

Supplementary Information

Peer Review File

### Source data

Source Data

## Data Availability

The experiment data which support the findings of this study are presented in this article and the Supplementary Information, and are available from the corresponding authors upon request. The source data underlying Figs. 1–6 are provided as a Source Data file. Source data are provided with this paper.
