# Supplementary material for: Single-atom Mo-tailored high-entropy-alloy ultrathin nanosheets with intrinsic tensile strain enhance electrocatalysis
Source: Nat Commun. 2024 Mar 13;15:2290. doi: 10.1038/s41467-024-45874-z (PMC10937678; doi:10.1038/s41467-024-45874-z)
Supplement: Supplementary file 1 — Supplementary Information [file 41467_2024_45874_MOESM1_ESM.pdf]

Supplementary Information

Single-atom Mo-tailored high-entropy-alloy ultrathin nanosheets with intrinsic tensile strain enhance electrocatalysis

Lin He[1,6], Menggang Li[1,2,6], Longyu Qiu[1,6], Shuo Geng[3], Yequn Liu[4], Fenyang Tian[1], Mingchuan Luo[2], Hu Liu[5], Yongsheng Yu[1]*, Weiwei Yang[1]* & Shaojun Guo[2]*

[1] State Key Laboratory of Urban Water Resource and Environment, School of Chemistry and Chemical Engineering, Harbin Institute of Technology, Harbin, Heilongjiang 150001, China.

[2] School of Materials Science and Engineering, Peking University, Beijing 100871, China.

[3] Department of Chemical Engineering, School of Chemistry and Chemical Engineering, Guizhou University, Guiyang 550025, China.

[4] Analytical Instrumentation Center, State Key Laboratory of Coal Conversion, Institute of Coal Chemistry, Chinese Academy of Sciences, Taiyuan, Shanxi 030001, China.

[5] School of Chemistry and Chemical Engineering, Xi'an University of Architecture and Technology, Xi'an, 710055 China.

[6] These authors contributed equally: Lin He, Menggang Li, and Longyu Qiu.

* Corresponding author: ysyu@hit.edu.cn (Y. Yu); yangww@hit.edu.cn (W. Yang); guosj@pku.edu.cn (S. Guo).

Supplementary Figures and Tables:

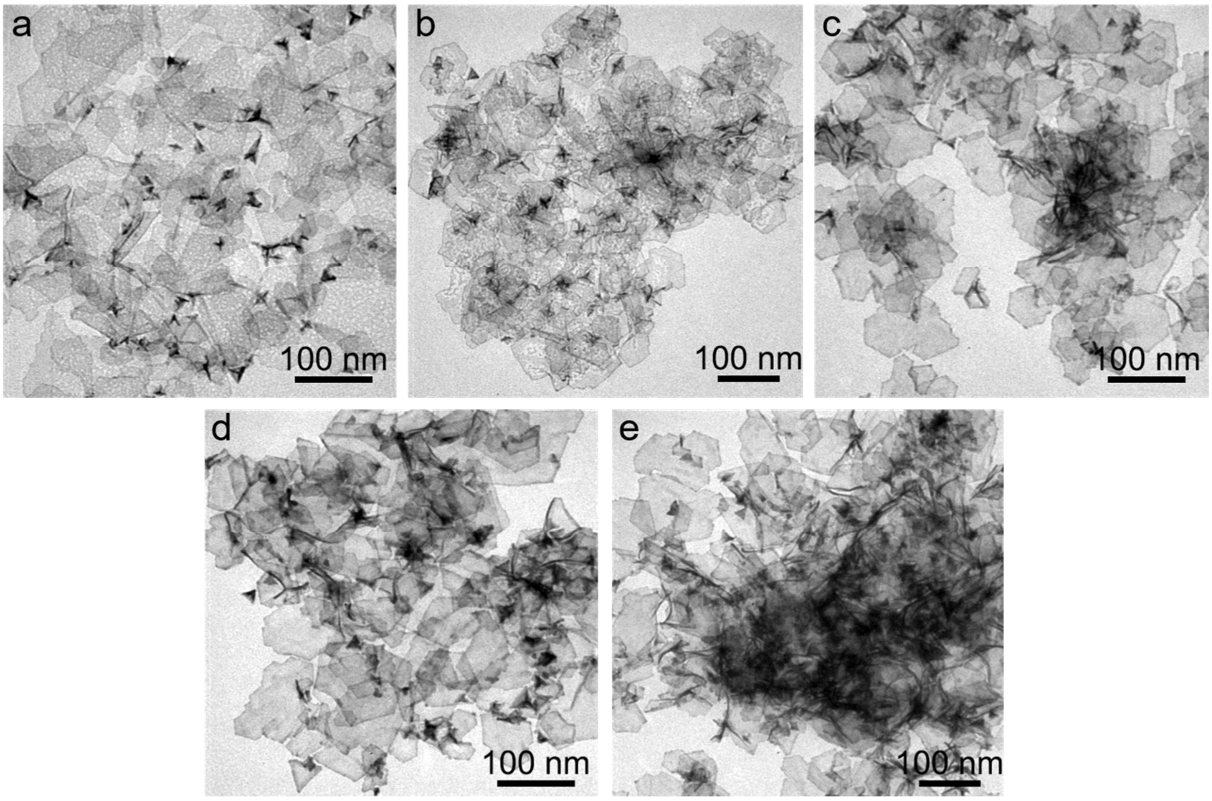

Supplementary Fig. 1 TEM images of Mo_1-PdPtNiCuZn SAHEA NSs intermediates collected at (a) 85 ºC, (b) 140 ºC, (c) 200 ºC with the reaction time of 0 min, (d) 200 ºC with the reaction time of 5 min, and (e) 200 ºC with the reaction time of 60 min.

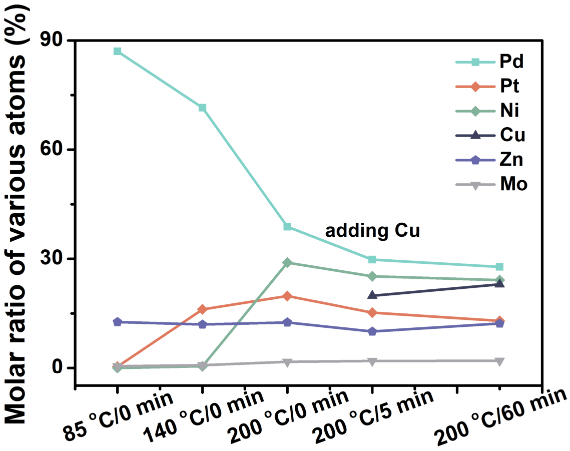

Supplementary Fig. 2 The changes in the composition ratio of various atoms for Mo_1-PdPtNiCuZn SAHEA NSs intermediates, as determined by ICP-OES measurements.

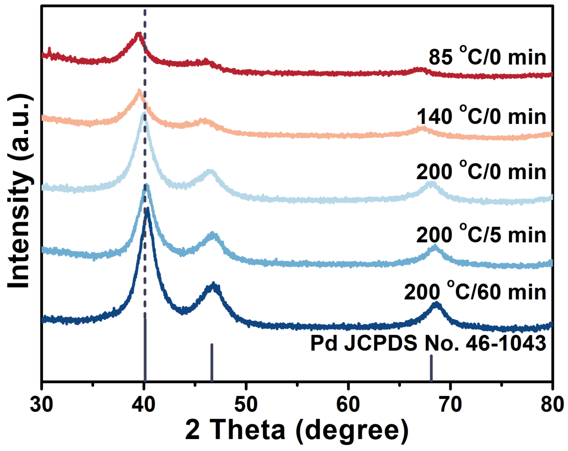

Supplementary Fig. 3 XRD patterns of Mo_1-PdPtNiCuZn SAHEA NSs intermediates.

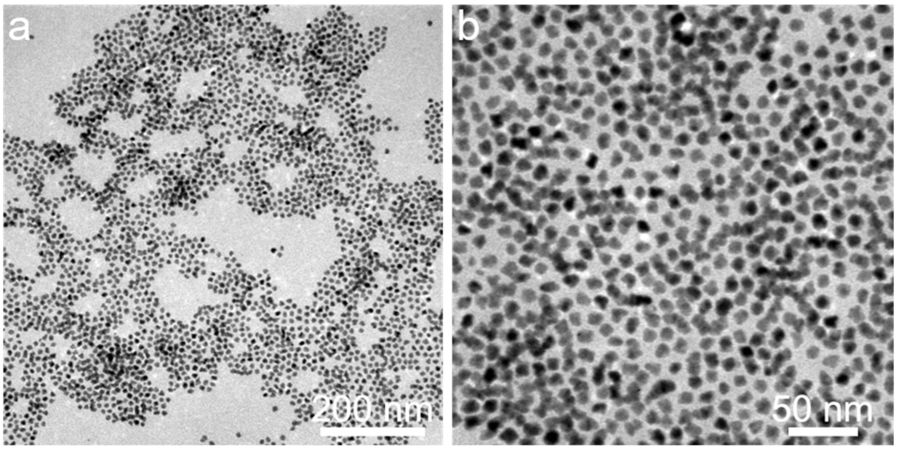

Supplementary Fig. 4 Representative (a) low- and (b) high-magnification TEM images of the products with the same reaction conditions as those of Mo_1-PdPtNiCuZn SAHEA NSs except using 0 mg of $Pd(acac)_2$.

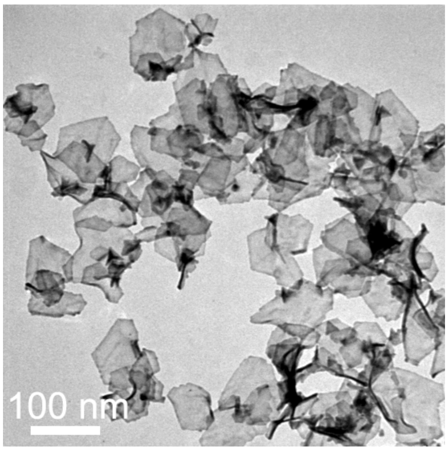

Supplementary Fig. 5 TEM image of Mo_1-PdPtNiCuZn SAHEA NSs.

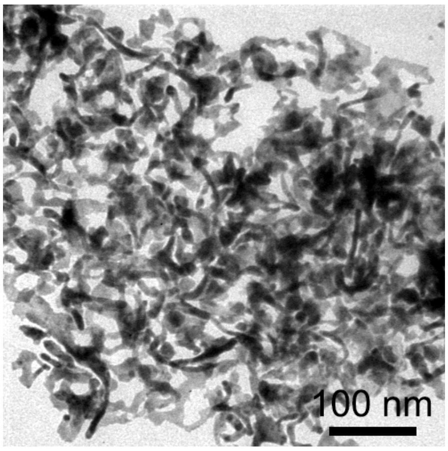

Supplementary Fig. 6 TEM image of the products with the same reaction conditions as those of Mo_1-PdPtNiCuZn SAHEA NSs except introducing more O_2 into the Cu precursor solution.

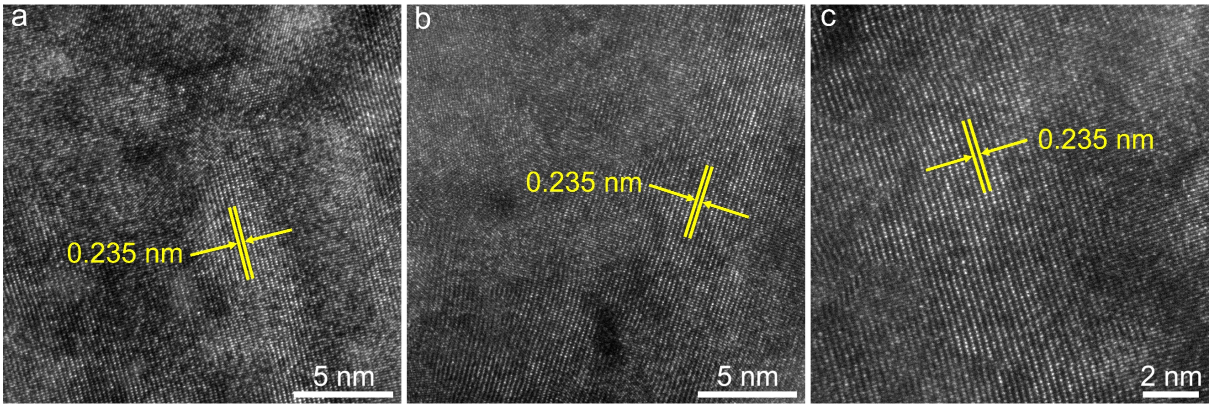

Supplementary Fig. 7 Additional HAADF-STEM analyses of Mo_1-PdPtNiCuZn SAHEA NSs. (a-c) Aberration-corrected HAADF-STEM images of the surface atomic arrangement on different Mo_1-PdPtNiCuZn SAHEA NSs.

The calculation of localized intrinsic tensile strain follows the equation below: $s(\text{SAHEA}) = (d_{\text{SAHEA}} - d_{\text{bulk}})/d_{\text{bulk}}$, where d_{SAHEA} and d_{bulk} represent the lattice constant of Mo_1-PdPtNiCuZn SAHEA NSs (measured to be 0.235 nm *via* the aberration-corrected HAADF-STEM) and PdPt bulk (0.225 nm), respectively. In addition, the overall tensile strain can be determined by Vegard's law and XRD pattern. Specifically, the lattice constant of MoPdPtNiCuZn solid solution bulk (S-bulk) with the same composition as Mo_1-PdPtNiCuZn SAHEA NSs (Pd/Pt/Ni/Cu/Zn/Mo = 26.9/13.3/24.8/21.9/11.0/2.1) can be calculated to be 0.2144 nm according to Vegard's law, and the lattice constant of the overall Mo_1-PdPtNiCuZn SAHEA nanosheets can be determined to be 0.2238 nm according to the XRD pattern of Mo1-PdPtNiCuZn SAHEA nanosheets. Therefore, the tensile strain of the overall Mo_1-PdPtNiCuZn SAHEA nanosheets can be calculated to be ~4.4%, according to the equation: $s(\text{O-SAHEA}) = (d_{\text{O-SAHEA}} - d_{\text{S-bulk}})/d_{\text{S-bulk}}$. These results reveal that the 2D wavy suprathin nanosheet structure can induce the accommodated intrinsic tensile strain of ~4.4%.

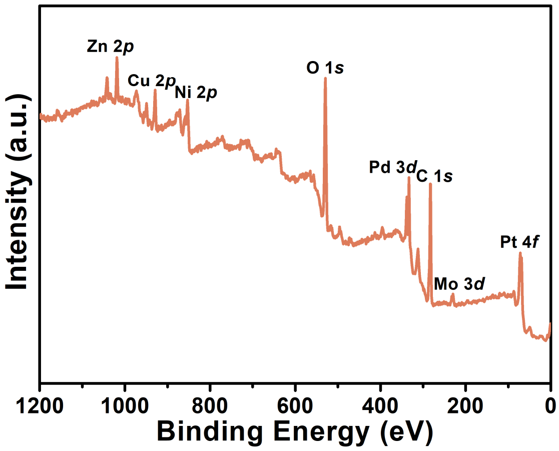

Supplementary Fig. 8 XPS survey spectrum of Mo_1-PdPtNiCuZn SAHEA NSs.

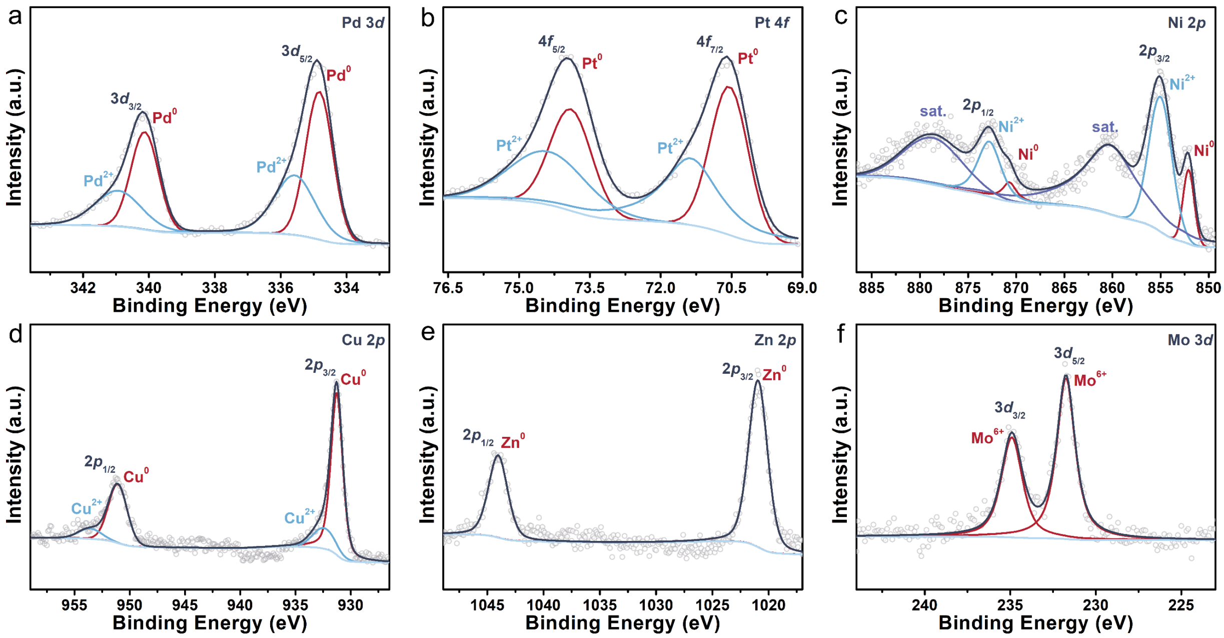

Supplementary Fig. 9 XPS compositional analysis of Mo_1-PdPtNiCuZn SAHEA NSs. (a) Pd 3*d* XPS spectrum. (b) Pt 4*f* XPS spectrum. (c) Ni 2*p* XPS spectrum. (d) Cu 2*p* XPS spectrum. (e) Zn 2*p* XPS spectrum. (f) Mo 3*d* XPS spectrum.

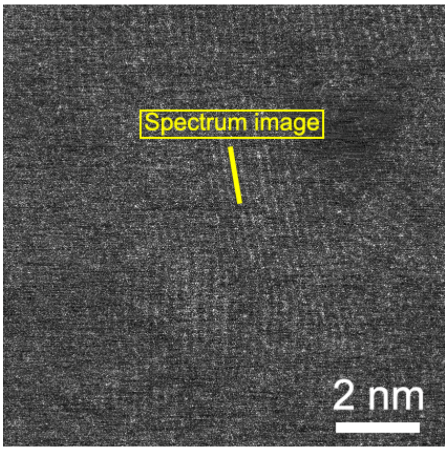

Supplementary Fig. 10 HAADF-STEM image of Mo_1-PdPtNiCuZn SAHEA NSs.

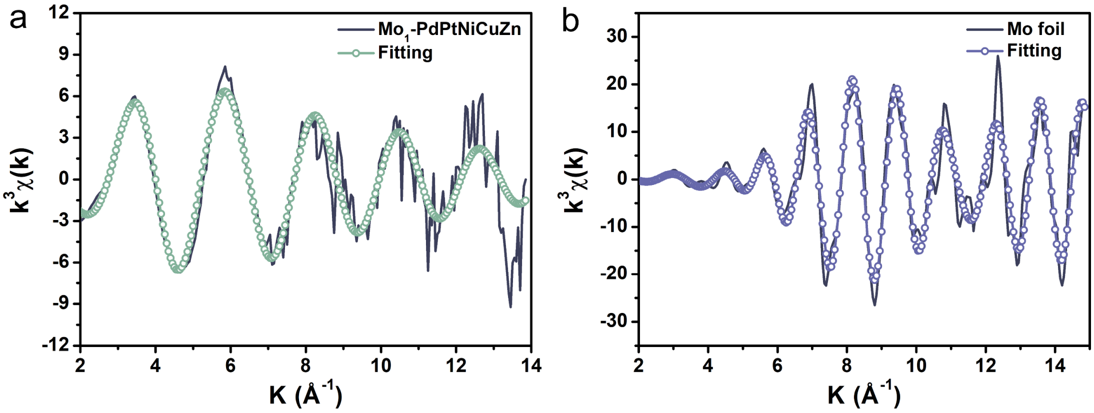

Supplementary Fig. 11 k space fitting curves of (a) Mo_1-PdPtNiCuZn SAHEA NSs and (b) Mo foil at Mo K-edge.

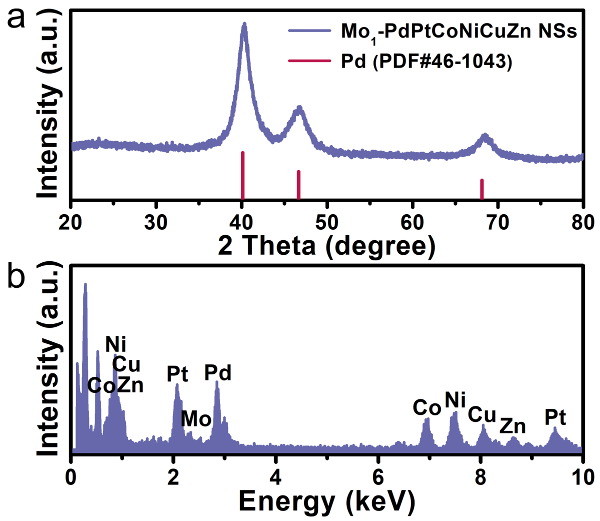

Supplementary Fig. 12 Structural and compositional characterizations of senary Mo_1-PdPtCoNiCuZn SAHEA NSs. (a) XRD pattern. (b) STEM-EDS spectra.

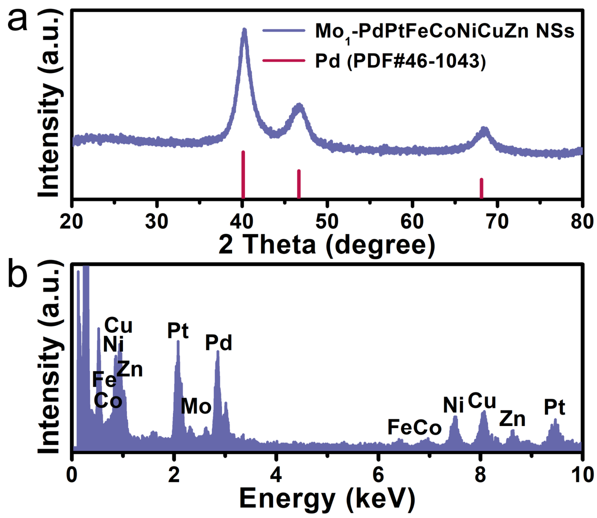

Supplementary Fig. 13 Structural and compositional characterizations of septenary Mo_1-PdPtFeCoNiCuZn SAHEA NSs. (a) XRD pattern. (b) STEM-EDS spectra.

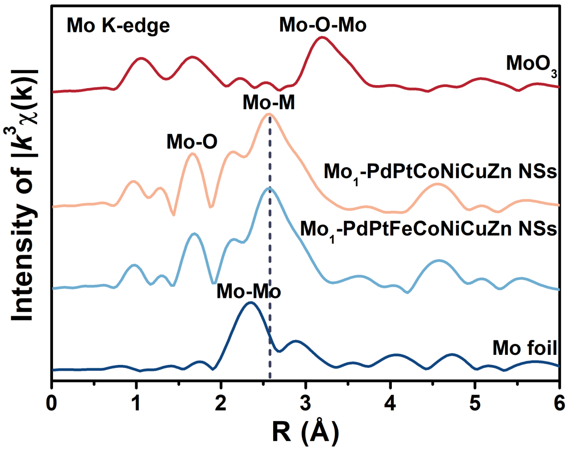

Supplementary Fig. 14 k^3-weighted Fourier transformation of the Mo K-edge EXAFS spectra.

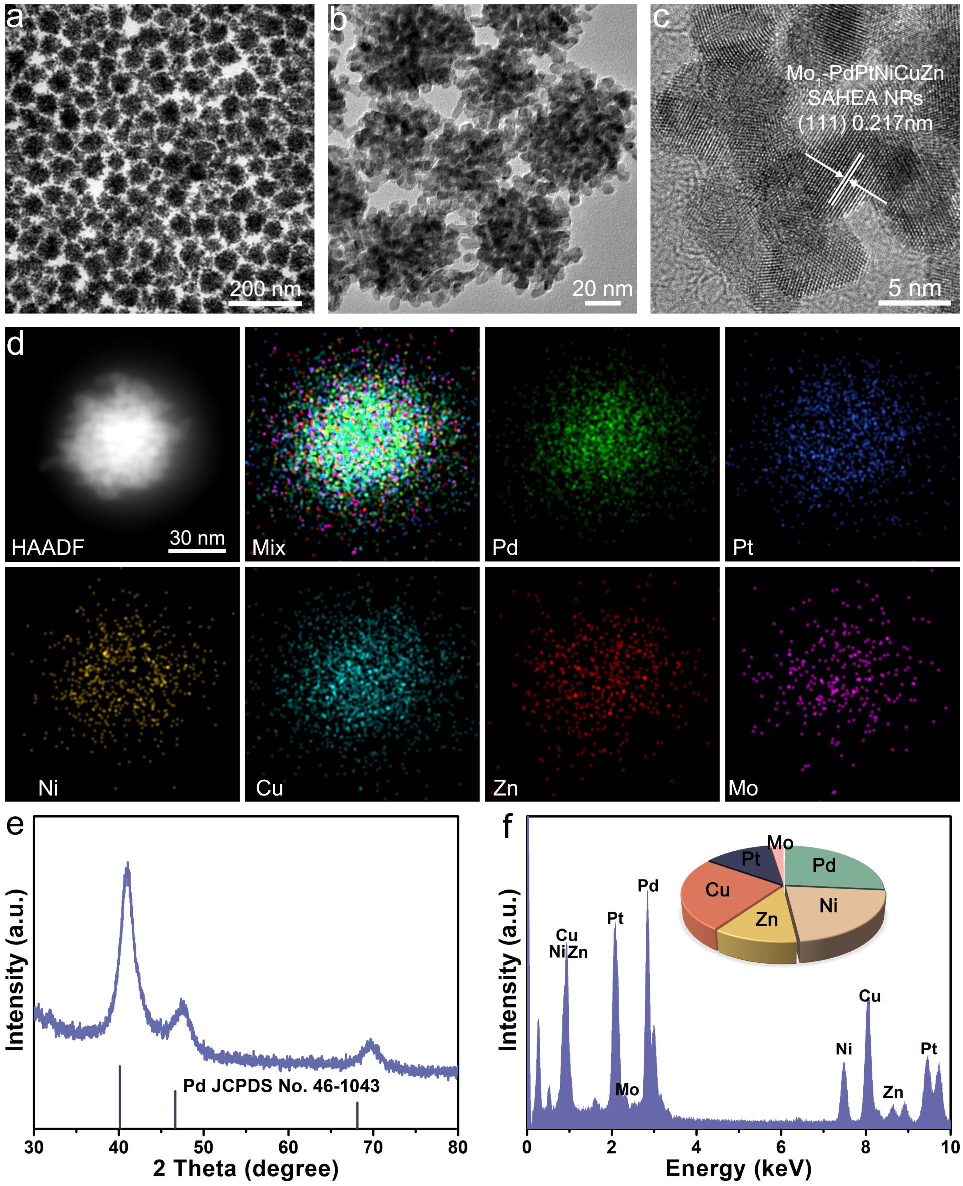

Supplementary Fig. 15 Morphological, structural, and compositional characterizations of Mo_1-PdPtNiCuZn SAHEA NPs. (a, b) TEM images. (c) HRTEM image. (d) HAADF-STEM image and elemental EDS mapping. (e) XRD pattern. (f) SEM-EDS spectra (*inset* is the corresponding ICP-OES result).

94
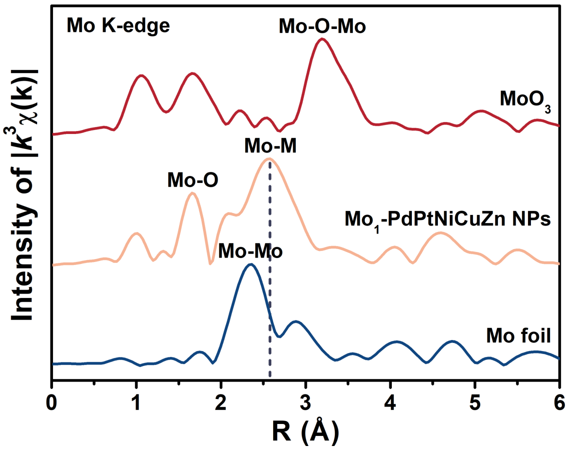

95 **Supplementary Fig. 16** k^3-weighted Fourier transformation of the Mo K-edge EXAFS spectra.

96

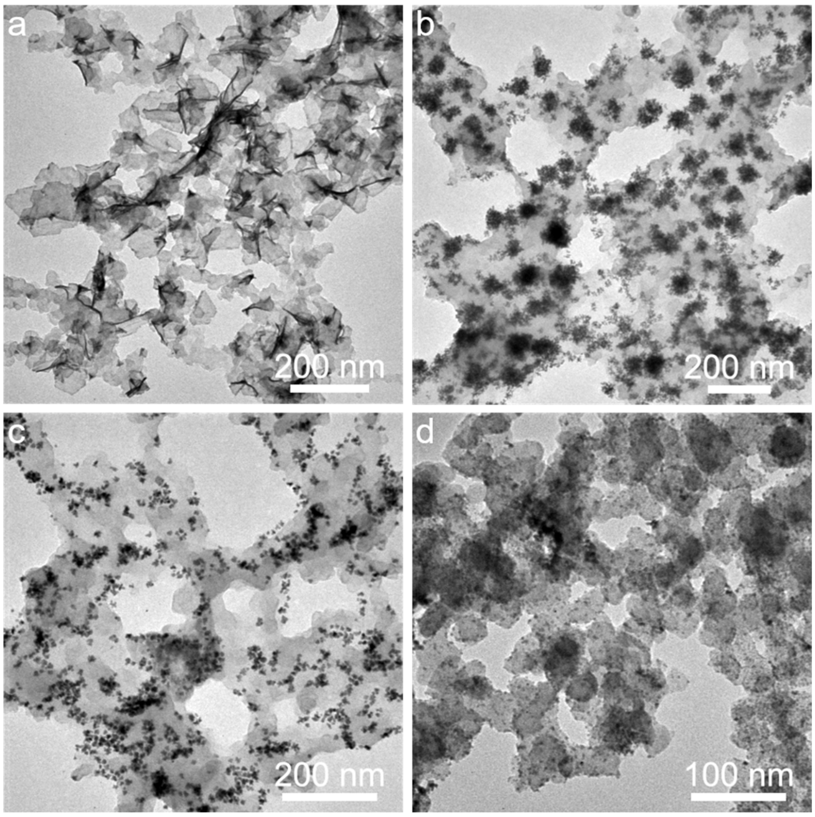

Supplementary Fig. 17 TEM images of carbon-supported (a) Mo_1-PdPtNiCuZn SAHEA NSs, (b) Mo_1-PdPtNiCuZn SAHEA NPs, (c) PdPtNiCuZn HEA NPs, and (d) commercial Pt/C.

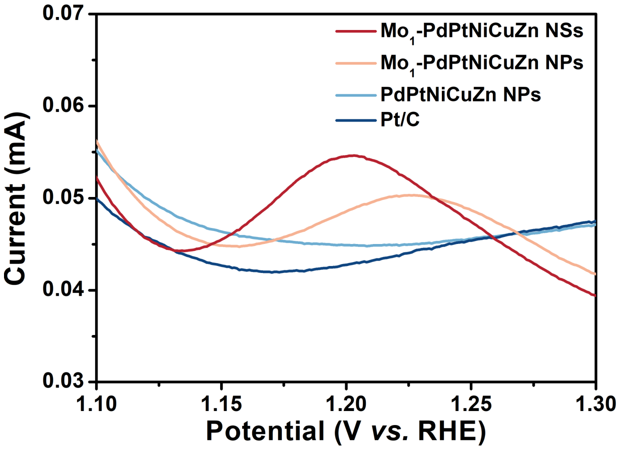

Supplementary Fig. 18 Magnified CV curves of Mo_1-PdPtNiCuZn SAHEA NSs, Mo_1-PdPtNiCuZn SAHEA NPs, PdPtNiCuZn HEA NPs and Pt/C in N_2-saturated 0.1 M $HClO_4$ solution.

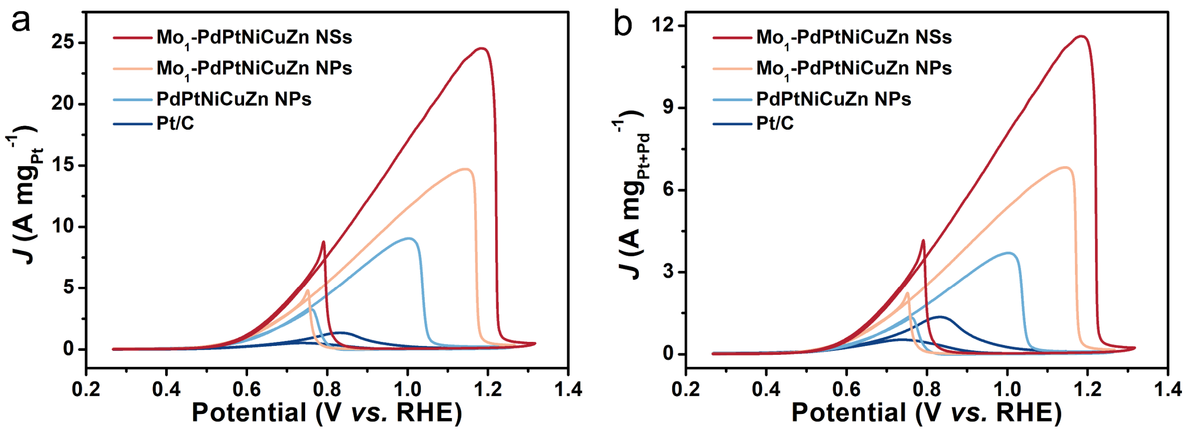

Supplementary Fig. 19 MOR profiles of Mo_1-PdPtNiCuZn SAHEA NSs, Mo_1-PdPtNiCuZn SAHEA NPs, PdPtNiCuZn HEA NPs, and Pt/C in 1.0 M KOH containing 1.0 M methanol. (a) Pt mass-normalized MOR CV profiles. (b) Pt + Pd mass-normalized MOR profiles.

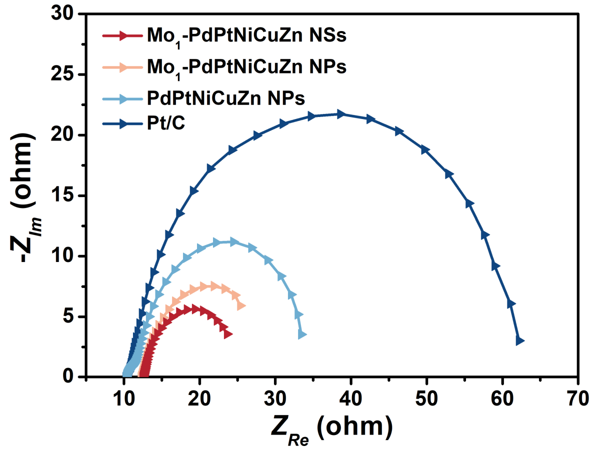

Supplementary Fig. 20 EIS plots of Mo_1-PdPtNiCuZn SAHEA NSs, Mo_1-PdPtNiCuZn SAHEA NPs, PdPtNiCuZn HEA NPs, and Pt/C under 0.75 V *vs.* RHE.

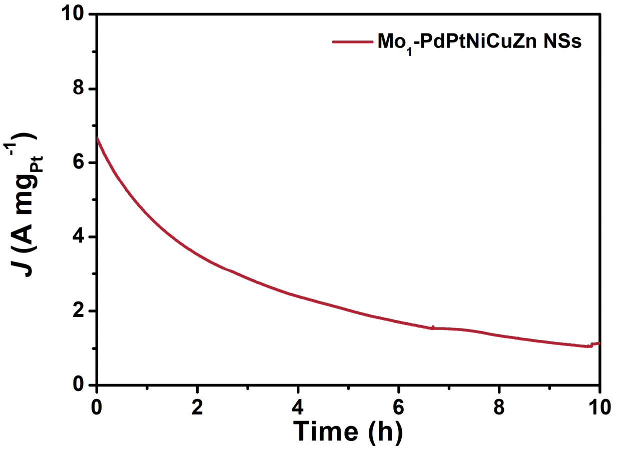

Supplementary Fig. 21 Chronoamperometric tests of Mo_1-PdPtNiCuZn SAHEA NSs for MOR at 0.77 V *vs.* RHE.

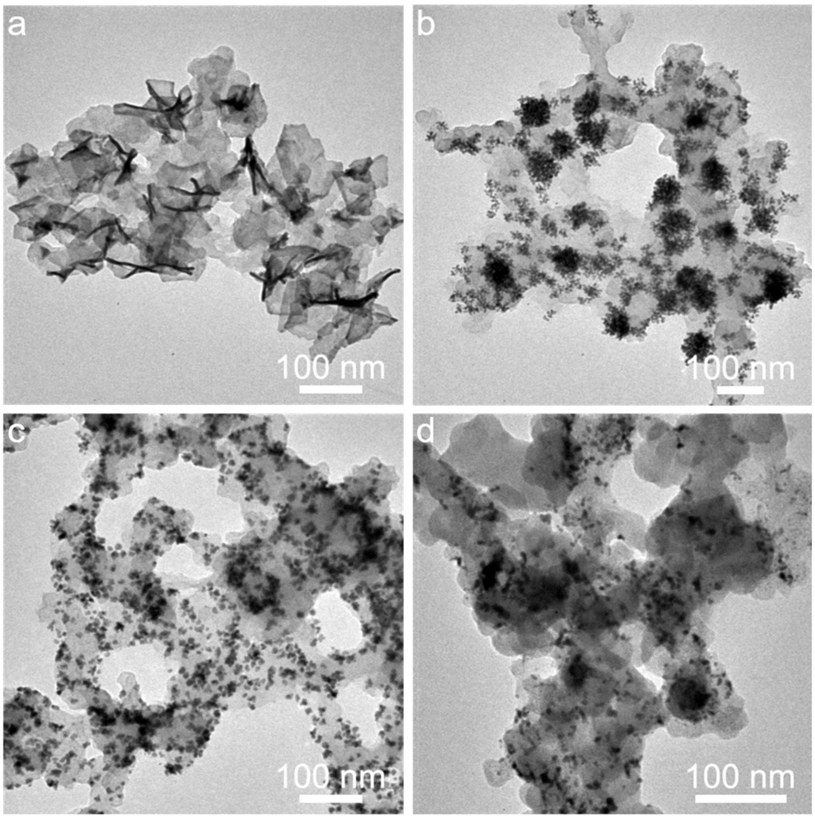

Supplementary Fig. 22 TEM images of carbon-supported (a) Mo_1-PdPtNiCuZn SAHEA NSs, (b) Mo_1-PdPtNiCuZn SAHEA NPs, (c) PdPtNiCuZn HEA NPs, and (d) commercial Pt/C after MOR stability tests.

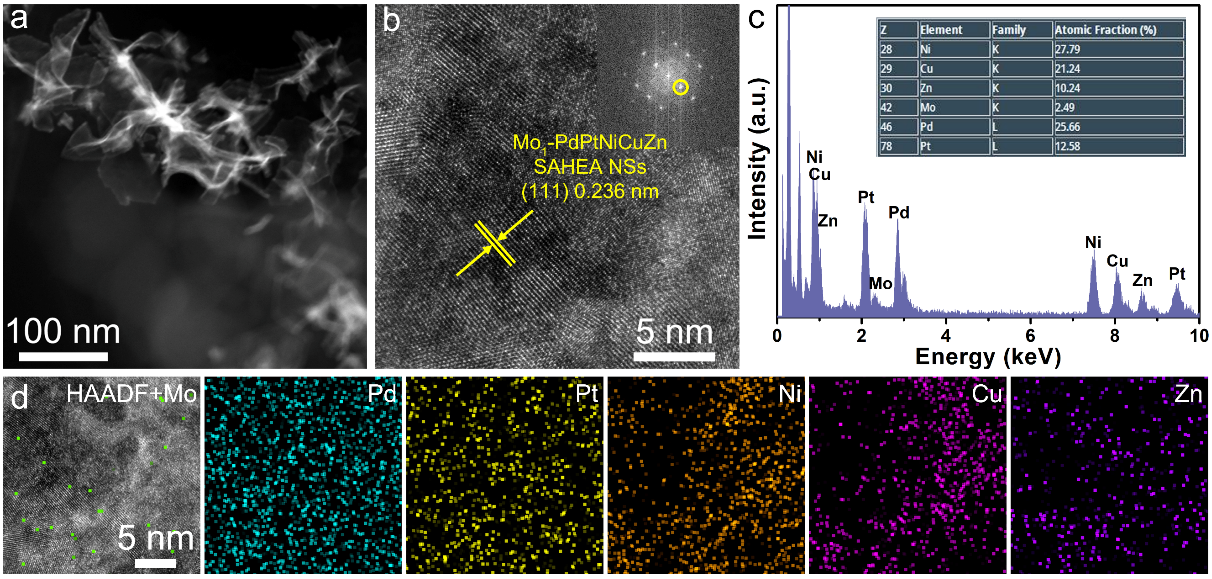

Supplementary Fig. 23 Morphological, structural, and compositional characterizations of Mo_1-PdPtNiCuZn SAHEA NSs after MOR stability tests. (a) Representative HAADF-STEM image. (b) High-resolution HAADF-STEM image (inset is the FFT pattern). (c) STEM-EDS and atomic ratio of different elements. (d) High-resolution HAADF-STEM-EDS elemental mapping.

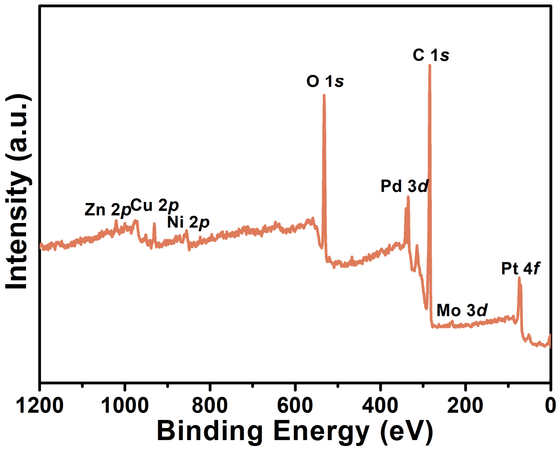

Supplementary Fig. 24 XPS survey spectrum of Mo_1-PdPtNiCuZn SAHEA NSs/C after MOR stability tests.

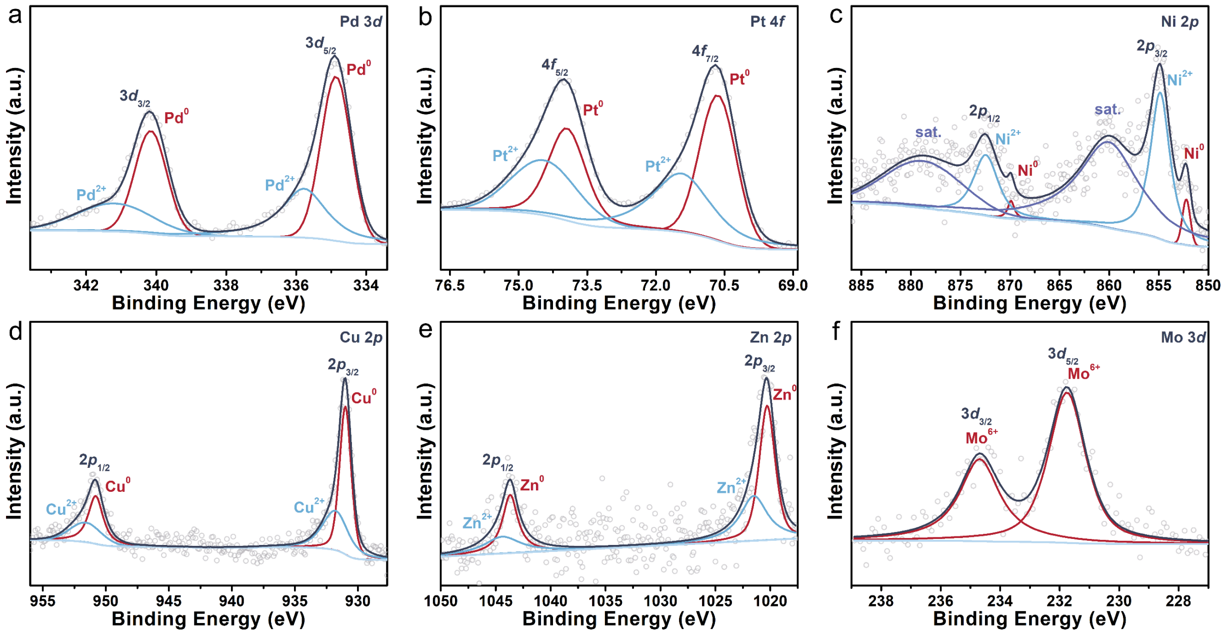

Supplementary Fig. 25 XPS compositional analysis of Mo_1-PdPtNiCuZn SAHEA NSs after MOR stability tests. (a) Pd 3*d* XPS spectrum. (b) Pt 4*f* XPS spectrum. (c) Ni 2*p* XPS spectrum. (d) Cu 2*p* XPS spectrum. (e) Zn 2*p* XPS spectrum. (f) Mo 3*d* XPS spectrum.

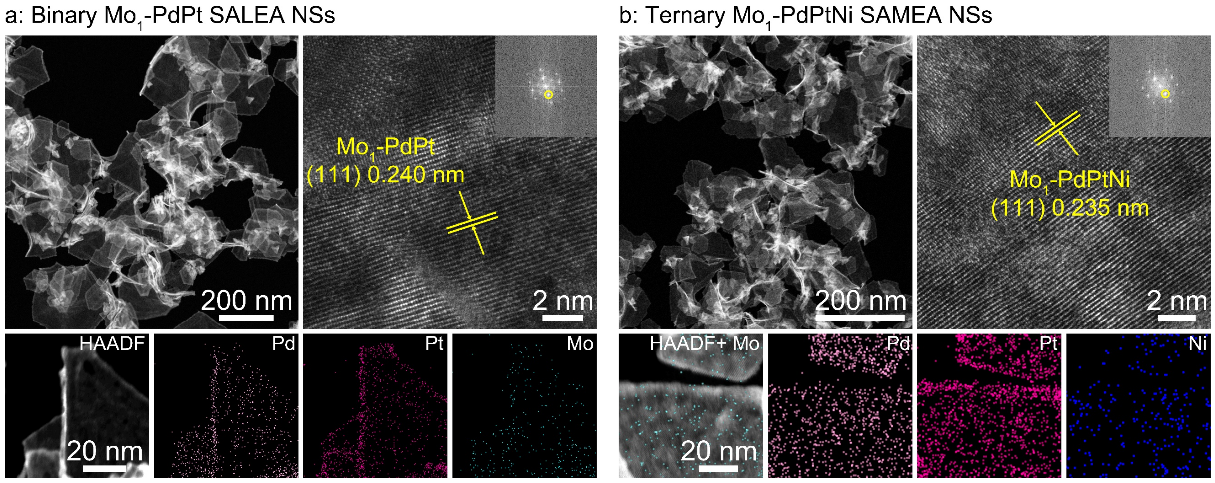

Supplementary Fig. 26 Morphological, compositional, and structural characterizations of Mo-tailored LEA and MEA NSs. (a) HAADF-STEM image, high-resolution HAADF-STEM image, FFT pattern, and HAADF-STEM-EDS elemental mapping of binary Mo_1-PdPt SALEA NSs. (b) HAADF-STEM image, high-resolution HAADF-STEM image, FFT pattern, and HAADF-STEM-EDS elemental mapping of ternary Mo_1-PdPtNi SAMEA NSs.

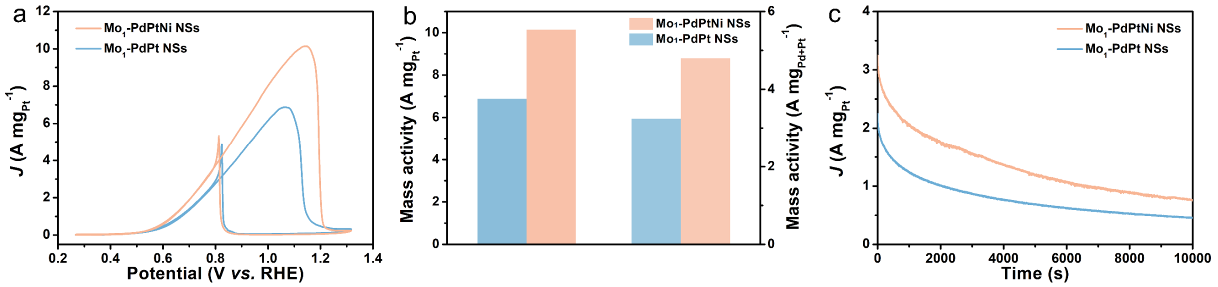

Supplementary Fig. 27 MOR performances of Mo-tailored LEA and MEA NSs in alkaline electrolytes. (a) Pt mass-normalized MOR profiles. (b) MOR mass activity of Mo_1-PdPt SALEA NSs and Mo_1-PdPtNi SAMEA NSs in 1.0 M KOH containing 1.0 M methanol. (c) Chronoamperometric tests for MOR at 0.77 V *vs*. RHE of Mo_1-PdPt SALEA NSs and Mo_1-PdPtNi SAMEANSs.

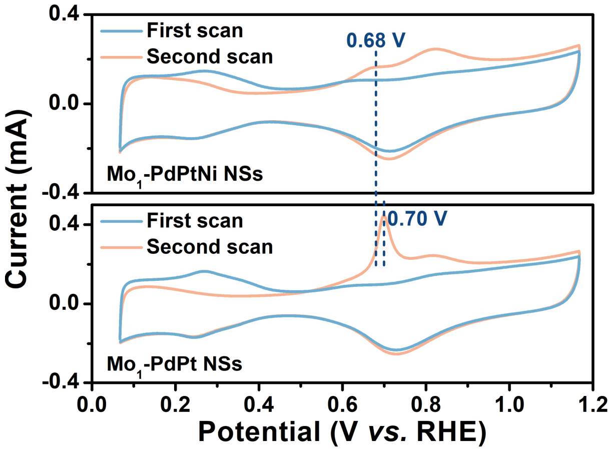

Supplementary Fig. 28 CO-stripping curves of Mo_1-PdPt SALEA NSs and Mo_1-PdPtNi SAMEA NSs catalysts recorded in N_2-saturated 1.0 M KOH.

The results reveal that methanol oxidation to CO_2 on these Mo-tailored LEA and MEA NSs remains through a dual-path mechanism. The oxidation peak of the absorbed CO on the Mo-tailored PdPt (Mo_1-PdPt) low-entropy-alloy (LEA) NSs is 0.70 V *vs.* RHE, 50 mV negative shift relative to that of Pt/C (0.75 V *vs.* RHE), further indicating that the introduction of single-atom Mo promoter and intrinsic tensile strain can accelerate the further oxidation of CO intermediate. Of note, different from the massive formation of CO_{ads} on Mo_1-PdPt SALEA NSs and commercial Pt/C, CO_{ads} on Mo-tailored PdPtNi (Mo_1-PdPtNi) medium-entropy-alloy (MEA) NSs dramatically decreased. Meanwhile, the oxidation peak of the absorbed CO on Mo_1-PdPtNi SAMEA NSs negatively shifts to 0.68 V *vs.* RHE relative to that of Mo_1-PdPt SALEA NSs, confirming that the introduction of additional elements can dilute Pt-Pt ensembles, significantly reducing the formation of CO_{ads} and concurrently further accelerating the removal of CO_{ads}.

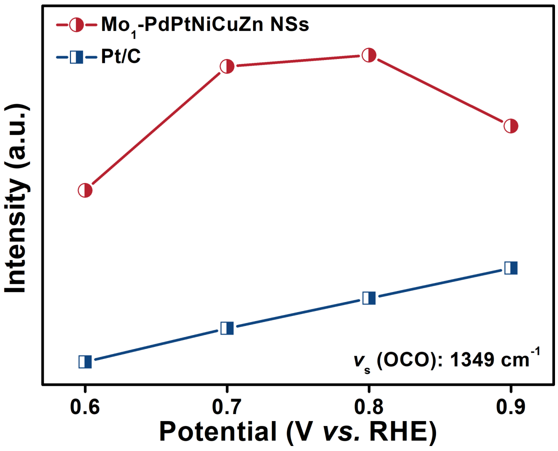

Supplementary Fig. 29 Dependence of IR intensity of v_s (OCO) (1349 cm^{-1}) of formate on the electrode potential.

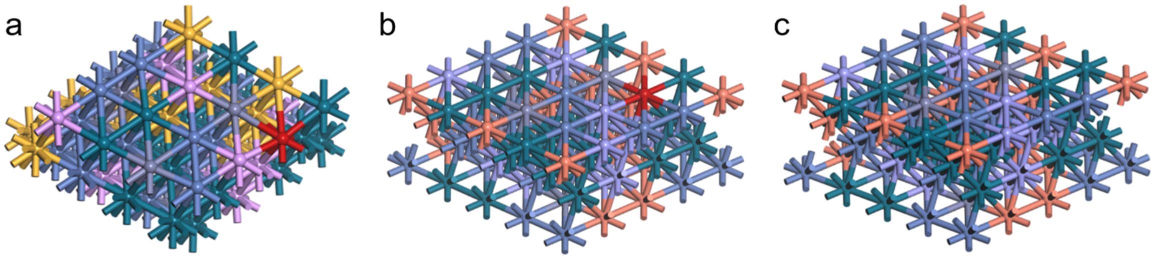

Supplementary Fig. 30 The structural configuration of (a) strained Mo_1-PdPtNiCuZn, (b) Mo_1-PdPtNiCuZn, and (c) PdPtNiCuZn.

We constructed the corresponding computation model based on the experimentally obtained structural and component information of Mo_1-PdPtNiCuZn. Specifically, we selected an appropriate supercell with a sufficient size capable of accommodating a wide variety of atoms and producing the desired disorder. Different atoms were randomly placed in the supercell to generate an initial idealized random structure. Energy calculations were performed to find the most stable structure of Mo_1-PdPtNiCuZn and subsequently to investigate the mechanism of methanol oxidation reaction on its surface.

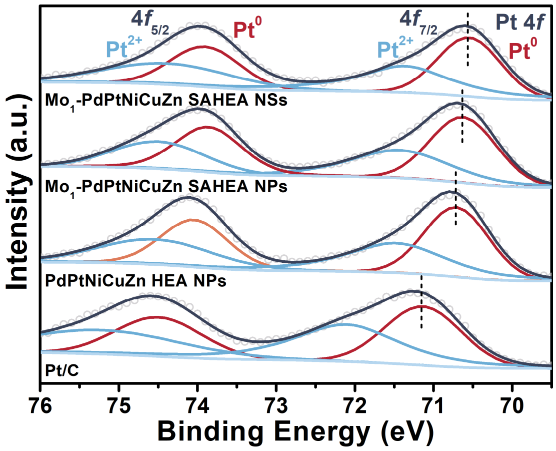

Supplementary Fig. 31 The Pt 4*f* XPS spectra of the Mo_1-PdPtNiCuZn SAHEA NSs, Mo_1-PdPtNiCuZn SAHEA NPs, PdPtNiCuZn HEA NPs and Pt/C.

Supplementary Table 1 Atomic ratio of Pd/Pt/Ni/Cu/Zn/Mo in Mo_1-PdPtNiCuZn SAHEA nanosheets detected by ICP-MS, ICP-OES, and STEM-EDS tests.

**Detected methods**	**Pd**	**Pt**	**Ni**	**Cu**	**Zn**	**Mo**
ICP-MS	24.1	14.6	26.3	22.1	11.1	1.8
ICP-OES	26.9	13.3	24.8	21.9	11.0	2.1
STEM-EDS	25.8	12.3	24.7	22.3	12.2	2.7

Supplementary Table 2 Mo K-edge EXAFS fitting parameters for various samples (S_0^2 = 0.914).

**Sample**	**Scattering path**	N^a	R(Å)^b	σ^2(Å^2)^c	ΔE_0(eV)^d	***R* factor**
Mo foil	Mo-Mo	8	2.72	0.0037	-5.773	0.0030
	Mo-Mo	6	3.13	0.0038		
Mo_1-PdPtNiCuZn	Mo-Mo1	1.459	1.81	-0.0001	8.338	0.0087
	Mo-Mo2	1.459	1.71	0.0010		

$^a N$: coordination numbers; $^b R$: bond distance; $^c \sigma^2$: Debye-Waller factors; $^d \Delta E_0$: the inner potential correction; R factor: goodness of fit. S_0^2 was set to 0.914, according to the experimental EXAFS fit of Mo foil reference by fixing coordination numbers as the known crystallographic value.

Error bounds obtained by EXAFS data were approximately estimated as N ± 10~30%; R ± 0.5~1%; σ^2 ± 10~30%; ΔE_0 ± 10~30%.

Supplementary Table 3 A summary of the performances of MOR electrocatalysts in alkaline electrolytes in literature.

**Catalysts**	**Pt-mass-normalized activity (A mg_{Pt}^{-1})**	**Noble-metal-mass-normalized activity (A mg_{PGM}^{-1})**	**ECSA-normalized activity (mA cm^{-2})**	**Electrolytes**	**References**
**Mo_1-PdPtNiCuZn SAHEA nanosheets**	**24.55**	**11.62**	**16.55**	**1.0 M KOH + 1.0 M methanol**	**This work**
PtNiGaSnMoRe HEA senary nanowires	6.20	-	12.31	1.0 M KOH + 1.0 M methanol	1
$Pt_{18}Ni_{26}Fe_{15}Co_{14}Cu_{27}$ HEA nanoparticles	15.04	-	-	1.0 M KOH + 1.0 M methanol	2
PdAgSn/PtBi HEA nanoparticles	-	2.87	4.67	1.0 M KOH + 1.0 M methanol	3
PdPt-based HEA-nanoparticles-(14)	12.60	-	-	1.0 M KOH + 1.0 M methanol	4
Pt clusters/$Ti_3C_2T_x$	7.32	-	-	1.0 M KOH + 1.0 M methanol	5
Au@PdPt	-	4.83	6.90	1.0 M KOH + 1.0 M methanol	6
Au@Pt_1-Pd_1 hemispherical nanostructures	-	4.38	5.04	1.0 M KOH + 1.0 M methanol	7
o-$PdH_{0.43}$@Pt	32.51	3.68	-	1.0 M KOH + 1.0 M methanol	8
Pt_5Ce/C	9.13	-	32.74	1.0 M KOH + 1.0 M methanol	9
PtCo nanoframes	4.28	-	8.56	1.0 M KOH + 1.0 M methanol	10
Pt/N-doped graphdiyne (Pt^0	1.45	-	-	1.0 M KOH + 1.0 M methanol	11

atoms)					
SA Ni-Pt nanowires	7.93	-	7.48	1.0 M KOH + 1.0 M methanol	12
PtRhBiSnSb HEA nanoplates	-	19.53	-	1.0 M KOH + 1.0 M methanol	13
$Pd_3Pb/Pt_{2.37}Pb$ nanocubes	8.40	-	13.39	1.0 M KOH + 1.0 M methanol	14
NiPtSAA/graphdiyne	4.38	-	-	1.0 M KOH + 1.0 M methanol	15
$PdSn_{0.5}/Se\text{-}Ti_3C_2$	-	4.76	6.25	1.0 M KOH + 1.0 M methanol	16
$Pd_{(20\%)}\text{-}CeO_{2(20\%)}$/nitrogen-doped mesoporous carbon sphere	-	1.50	-	1.0 M KOH + 1.0 M methanol	17
Pt_1/RuO_2	6.77	-	-	1.0 M KOH + 1.0 M methanol	18
PtCuPd@Ru yolk-cage nanostructures	-	2.48	-	1.0 M KOH + 1.0 M methanol	19
PtZn intermetallic nanoparticles	0.58	-	1.14	1.0 M KOH + 1.0 M methanol	20
$Pt/Ni(OH)_2$/reduced graphene oxide	1.23	-	1.93	1.0 M KOH + 1.0 M methanol	21

References

1. Yang, F. et al. Ultrathin PtNiGaSnMoRe senary nanowires with partial amorphous structure enable remarkable methanol oxidation electrocatalysis. *Adv. Energy Mater.* **13**, 2301408 (2023).

2. Li, H. et al. Fast site-to-site electron transfer of high-entropy alloy nanocatalyst driving redox electrocatalysis. *Nat. Commun.* **11**, 5437 (2020).

3. Lao, X. et al. Pd-enriched-core/Pt-enriched-shell high-entropy alloy with face-centred cubic structure for C_1 and C_2 alcohol oxidation. *Angew. Chem. Int. Ed.* **62**, e202304510 (2023).

4. Wang, Y. et al. Synthesis of high-entropy-alloy nanoparticles by a step-alloying strategy as a superior multifunctional electrocatalyst. *Adv. Mater.* **35**, 2302499 (2023).

5. Zhu, J. et al. Ultrahigh stable methanol oxidation enabled by a high hydroxyl concentration on Pt clusters/MXene interfaces. *J. Am. Chem. Soc.* **144**, 15529-15538 (2022).

6. Yang, X. et al. Modulating electronic structure of an Au-nanorod-core-PdPt-alloy-shell catalyst for efficient alcohol electro-oxidation. *Adv. Energy Mater.* **11**, 2100812 (2021).

7. Liang, W. et al. 3D anisotropic Au@Pt-Pd hemispherical nanostructures as efficient electrocatalysts for methanol, ethanol, and formic acid oxidation reaction. *Adv. Mater.* **33**, 2100713 (2021).

8. Liu, G. et al. Hydrogen-intercalation-induced lattice expansion of Pd@Pt core-shell nanoparticles for highly efficient electrocatalytic alcohol oxidation. *J. Am. Chem. Soc.* **143**, 11262-11270 (2021).

9. Zhang, S. et al. Lanthanide electronic perturbation in Pt-Ln (La, Ce, Pr and Nd) alloys for enhanced methanol oxidation reaction activity. *Energ. Environ. Sci.* **14**, 5911-5918 (2021).

10. Chen, S. et al. High-performance Pt-Co nanoframes for fuel-cell electrocatalysis. *Nano Lett.* **20**, 1974-1979 (2020).

11. Hui, L. et al. Highly loaded independent Pt^0 atoms on graphdiyne for pH-general methanol oxidation reaction. *Adv. Sci.* **9**, 2104991 (2022).

12. Li, M. et al. Single-atom tailoring of platinum nanocatalysts for high-performance multifunctional electrocatalysis. *Nat. Catal.* **2**, 495-503 (2019).

13. Chen, W. et al. High-entropy intermetallic PtRhBiSnSb nanoplates for highly efficient alcohol oxidation electrocatalysis. *Adv. Mater.* **34**, 2206276 (2022).

14. Wu, X. et al. Tuning surface structure of Pd_3Pb/Pt_nPb nanocrystals for boosting the methanol oxidation reaction. *Adv. Sci.* **6**, 1902249 (2019).

15. Hui, L. et al. Atomic alloys of nickel-platinum on carbon network for methanol oxidation. *Nano Energy* **95**, 106984 (2022).

16. Chen, S. et al. Engineering support and distribution of palladium and tin on MXene with modulation of the d-band center for CO-resilient methanol oxidation. *Angew. Chem. Int. Ed.* **61**, e202209693 (2022).

17. Tan, Q. et al. Highly dispersed Pd-CeO_2 nanoparticles supported on N-doped core-shell structured mesoporous carbon for methanol oxidation in alkaline media. *ACS Catal.* **9**, 6362-6371 (2019).

18. Zhang, Z. et al. Single-atom catalyst for high-performance methanol oxidation. *Nat. Commun.* **12**, 5235 (2021).

19. Saleem, F. et al. Elemental segregation in multimetallic core-shell nanoplates. *J. Am. Chem. Soc.* **141**, 14496-14500 (2019).

20. Qi, Z. et al. Sub-4 nm PtZn intermetallic nanoparticles for enhanced mass and specific activities in catalytic electrooxidation reaction. *J. Am. Chem. Soc.* **139**, 4762-4768 (2017).

21. Huang, W. et al. Highly active and durable methanol oxidation electrocatalyst based on the synergy of platinum-nickel hydroxide-graphene. *Nat. Commun.* **6**, 10035 (2015).
