# Supplementary material for: Single-atom Mo-tailored high-entropy-alloy ultrathin nanosheets with intrinsic tensile strain enhance electrocatalysis
Source: Nat Commun. 2024 Mar 13;15:2290. doi: 10.1038/s41467-024-45874-z (PMC10937678; doi:10.1038/s41467-024-45874-z)
Supplement: Supplementary file 2 — Peer Review File [file 41467_2024_45874_MOESM2_ESM.pdf]

nature portfolio

Peer Review File

Single-atom Mo-tailored high-entropy-alloy ultrathin nanosheets with intrinsic tensile strain enhance electrocatalysis

Open Access This file is licensed under a Creative Commons Attribution 4.0 International License, which permits use, sharing, adaptation, distribution and reproduction in any medium or format, as long as you give appropriate credit to the original author(s) and the source, provide a link to the Creative Commons license, and indicate if changes were made. In the cases where the authors are anonymous, such as is the case for the reports of anonymous peer reviewers, author attribution should be to 'Anonymous Referee' followed by a clear attribution to the source work. The images or other third party material in this file are included in the article's Creative Commons license, unless indicated otherwise in a credit line to the material. If material is not included in the article's Creative Commons license and your intended use is not permitted by statutory regulation or exceeds the permitted use, you will need to obtain permission directly from the copyright holder. To view a copy of this license, visit http://creativecommons.org/licenses/by/4.0/.

REVIEWER COMMENTS

Reviewer #1 (Remarks to the Author):

The manuscript presents a novel approach in which single-atom Mo was introduced into PdPtNiCuZn nanosheets to enhance the electrocatalytic performance of the methanol oxidation reaction (MOR). The goal was to integrate single-atom and high-entropy-alloy (HEA) features to induce intrinsic tensile strain and dilute Pt-Pt ensembles, thereby improving electrocatalysis. The HEA nanosheets exhibited exceptional electrocatalytic behavior, demonstrating high mass activity and long-term durability. The Mo single atoms served as promoters by modifying the electronic structure of isolated Pt sites in the HEA host, effectively inhibiting the formation of CO adsorbates and promoting the methanol-to-formate pathway. Moreover, the synergistic effects of Mo promoters and tensile strain optimized the adsorption behavior of intermediates, leading to a more energetically favorable pathway and minimizing the barrier for the MOR. I recommend this paper for publication in your journal after addressing the following concerns.

1. The authors suggest that tensile strain due to the lattice distortion by Mo doping is the key factor for enhancing the electrocatalytic activity, but the evidence for the claim is insufficient. For example, it should be analyzed whether the lattice distortion does not occur in the PdPtNiCuZn without Mo doping.

2. The J-V curves for the methanol oxidation reaction, which is not normalized by Pt mass, should be provided in Fig 4 for comparison.

3. More detailed explanation is needed of which part of Fig 5b and 5c the sentence below corresponds to.

“The onset potentials, corresponding to the formate characteristic bands of Mo1-PdPtNiCuZn SAHEA NSs, are 100 mV lower than those of commercial Pt/C.”

4. In Fig 5c, the Pt/C also shows the vibration peak corresponding to formate, and the intensity of peaks is comparable with those of Mo-PdPtNiCuZn. Doesn't this mean that Pt/C can also convert methanol to formate? An additional explanation for this should be provided.

5. In Fig. 5d, the modeling of the reactant and product molecules needs to be clarified. They are hard to distinguish from the catalyst in the background. The Pt and Mo atoms also seem to be hard to find.

6. In Fig 6h, explain specifically for choosing U=0.67 V.

7. Explanation for the abbreviation SAHEA is absent.

Reviewer #2 (Remarks to the Author):

The manuscript presents the design of a novel class of efficient electrocatalysts: single-atom Mo-tailored PdPtNiCuZn high-entropy-alloy nanosheets. These nanosheets are specifically engineered to enhance the catalysis of the methanol oxidation reaction by promoting the formate pathway. While the authors have conducted experimental tests to investigate the reaction mechanism, certain aspects require further clarification.

1. The authors have mentioned that their synthetic method for single-atom Mo-tailored HEA NSs with tensile-strained allows for the expansion of the compositional space of HEA hosts. However, it is important to provide a deeper explanation and supporting evidence for this claim. The essential reasons behind how this synthetic method enables the expansion of the compositional space of HEA hosts should be clearly stated.

2. The authors have highlighted the superiority of using PdPtNiCuZn high-entropy-alloy nanosheets as carriers for Mo single atoms compared to conventional carrier materials. It would be beneficial if the authors could supplement their claims by including data of conventional carrier materials tested under the same experimental conditions. This would provide a direct comparison and support their argument.

3. The authors have proposed that dilute Pt-Pt ensembles and intrinsic tensile strain (Mo1-PdPtNiCuZn) enhance the methanol oxidation reaction. However, it is important to provide relevant evidence to support this claim.

4. The manuscript does not clearly address the stability of the single-atom Mo-tailored PdPtNiCuZn high-entropy-alloy nanosheets. It is crucial to discuss the stability of these nanosheets and provide evidence, such as long-term stability tests or characterization data.

Reviewer #3 (Remarks to the Author):

Dear Editor,

I have reviewed the submitted manuscript thoroughly and, regrettably, I must recommend against its publication.

This paper reported a class of single-atom Mo-tailored PdPtNiCuZn high-entropy-alloy (HEA) nanosheets for enhancing the methanol oxidation reaction (MOR) electrocatalysis. EDS mapping is used multiple times throughout the whole manuscript to demonstrate the single atom Mo on the HEAs. However, due to the poor resolution of the results, all these EDS results cannot prove that Mo exists as a single atom at all. Therefore, the experimental results cannot support the statements at all. Moreover, HEA electrocatalysts have been extensively studied and the study lacks novelty. Since the lack of novelty, substandard visual presentation, and unconvincing experimental results, I am constrained to recommend the publication of this manuscript.

My evaluation is based on several notable issues that are outlined below.

1. The noble metals chosen in the alloy turn out the studied material less competitive in comparison with those noble less high-entropy alloys.

2. ICP-OES shouldn't be used to detect single atom Mo accurately since ICP-OES is not sensitive to low concentration.

3. Provide evidence that concaves defects mainly arise from oxidative etching by a trace amount of O2 introduced during the addition of Cu precursors (Fig. 1g).

4. How 4.4% tensile strain is calculated? Is this the strain of the overall sample? Localized d-spacing can't prove strain for the whole sample since it is well known that high-entropy alloys are not that uniform in terms of the composition from region to region. Provide evidence for the strain.

5. EDS mappings through the whole manuscript can't prove that Mo atoms exist as single atoms given its high noise ratio and poor resolution. Again, given the highly noisy nature of EDS, it can't be used to demonstrate that Mo exists as single atoms on other HEA NSs. For example, in Figure S17, if Mo exists as single atoms, then Zn should also exist as single atoms given their similar results.

6. Z contrast can not show that Mo exists in single atoms since its atomic number is between other elements. Although atomic radius also can't prove single-atom Mo due to the low resolution, the circled atom in fact has a smaller atomic radius and therefore shouldn't be Mo that has a much larger atomic radius.

7. Evidence that ~ 1.33 Å can be attributed to Mo-O scattering from MoOx in SAHEAs.

8. There are grammar mistakes in the sentence "When Cu(acac)2 and other metal precursors were introduced simultaneously and kept other 5 / 10 conditions unchanged".

9. The following statement cannot be supported by the experimental results "As-obtained NPs exhibit the single-atom Mo-tailored HEA structures and the similar Pd/Pt/Ni/Cu/Zn/Mo compositions to that of SAHEA NSs."

10. Based on Figure S17, the Zn becomes obviously less after durability tests.

I hope the authors find these comments useful in improving their manuscript. However, Due to the extent of the issues, I maintain my recommendation against its publication in Nature Communication.

Response to reviewers

To Reviewer 1:

The manuscript presents a novel approach in which single-atom Mo was introduced into PdPtNiCuZn nanosheets to enhance the electrocatalytic performance of the methanol oxidation reaction (MOR). The goal was to integrate single-atom and high-entropy-alloy (HEA) features to induce intrinsic tensile strain and dilute Pt-Pt ensembles, thereby improving electrocatalysis. The HEA nanosheets exhibited exceptional electrocatalytic behavior, demonstrating high mass activity and long-term durability. The Mo single atoms served as promoters by modifying the electronic structure of isolated Pt sites in the HEA host, effectively inhibiting the formation of CO adsorbates and promoting the methanol-to-formate pathway. Moreover, the synergistic effects of Mo promoters and tensile strain optimized the adsorption behavior of intermediates, leading to a more energetically favorable pathway and minimizing the barrier for the MOR. I recommend this paper for publication in your journal after addressing the following concerns.

Response: We also appreciate your valuable comments on our manuscript. To address your concerns, we have improved our manuscript along with a point-by-point response as follows.

1. The authors suggest that tensile strain due to the lattice distortion by Mo doping is the key factor for enhancing the electrocatalytic activity, but the evidence for the claim is insufficient. For example, it should be analyzed whether the lattice distortion does not occur in the PdPtNiCuZn without Mo doping.

Response: Thank you so much for your valuable comment. In our work, the intrinsic tensile strain mainly arises from the 2D wavy nanosheet structure and the ultrathin thickness of Mo_1-PdPtNiCuZn single-atom high-entropy-alloy (SAHEA) nanosheets [R1], while the tensile strain due to doping of single-atom Mo is negligible. The lattice distortion is the intrinsic characteristic of high-entropy alloys (HEAs), mainly arising from the random occupation of metallic atoms with different sizes in the crystalline lattices [R2]. Therefore, the lattice distortion also occurs in PdPtNiCuZn HEAs without single-atom Mo doping.

References:

[R1] Lin, F. *et al.* Local coordination regulation through tuning atomic-scale cavities of Pd metallene toward efficient oxygen reduction electrocatalysis. *Adv. Mater.* **34**, 2202084 (2022).
[R2] Xin, Y. et al. High-entropy alloys as a platform for catalysis: progress, challenges, and opportunities. *ACS Catal.* **10**, 11280-11306 (2020).

2. The *J-V* curves for the methanol oxidation reaction, which is not normalized by Pt mass, should be provided in Fig 4 for comparison.

Response: Thank you so much for your constructive suggestion. In addition to Pt mass-normalized MOR curves, we further normalized the MOR curves of Mo_1-PdPtNiCuZn SAHEA nanosheets, Mo_1-PdPtNiCuZn SAHEA nanoparticles, PdPtNiCuZn HEA nanoparticles, and commercial Pt/C to the electrochemically active surface areas (ECSAs) (Fig. R1). We have included the normalized curves in the Fig. 4 and the corresponding discussion in the revised manuscript.

"*Moreover, the constructed Mo_1-PdPtNiCuZn SAHEA NSs with intrinsic tensile strain deliver the highest MOR specific activity of 16.55 mA cm^{-2}, obviously higher than those of Mo_1-PdPtNiCuZn SAHEA NPs and most of reported Pd/Pt-based electrocatalysts, and also 7.13 times higher than that*

of commercial Pt/C (Supplementary Table 3)."

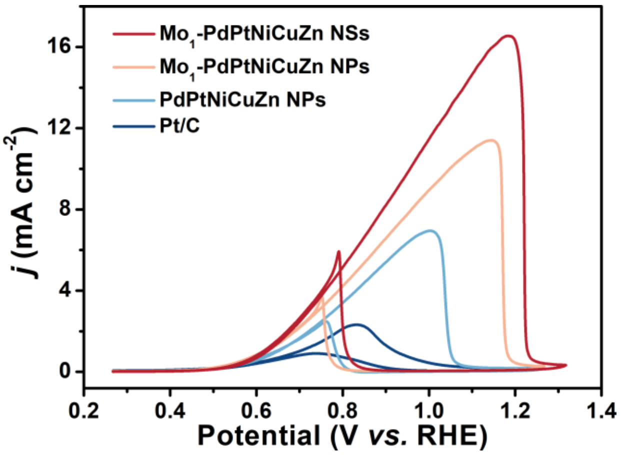

Figure R1. ECSA-normalized MOR curves of Mo_1-PdPtNiCuZn SAHEA nanosheets, Mo_1-PdPtNiCuZn SAHEA nanoparticles, PdPtNiCuZn HEA nanoparticles, and commercial Pt/C in 1.0 M KOH containing 1.0 M methanol.

3. More detailed explanation is needed of which part of Fig 5b and 5c the sentence below corresponds to.

"The onset potentials, corresponding to the formate characteristic bands of Mo_1-PdPtNiCuZn SAHEA NSs, are 100 mV lower than those of commercial Pt/C."

Response: Thank you so much for your constructive comment. We have highlighted the curves corresponded to the onset potentials and added explanations (the values of onset potentials) in Fig. 5b and 5c (Fig. R2). We have also included the detailed instructions about onset potentials corresponding to formate characteristic bands in the revised manuscript.

"*The onset potential for producing the formate (characteristic bands of 1585, 1378, 1349, and 1313 cm^{-1}) over Mo_1-PdPtNiCuZn SAHEA NSs is 0.45 V, 100 mV lower than that of commercial Pt/C (0.55 V).*"

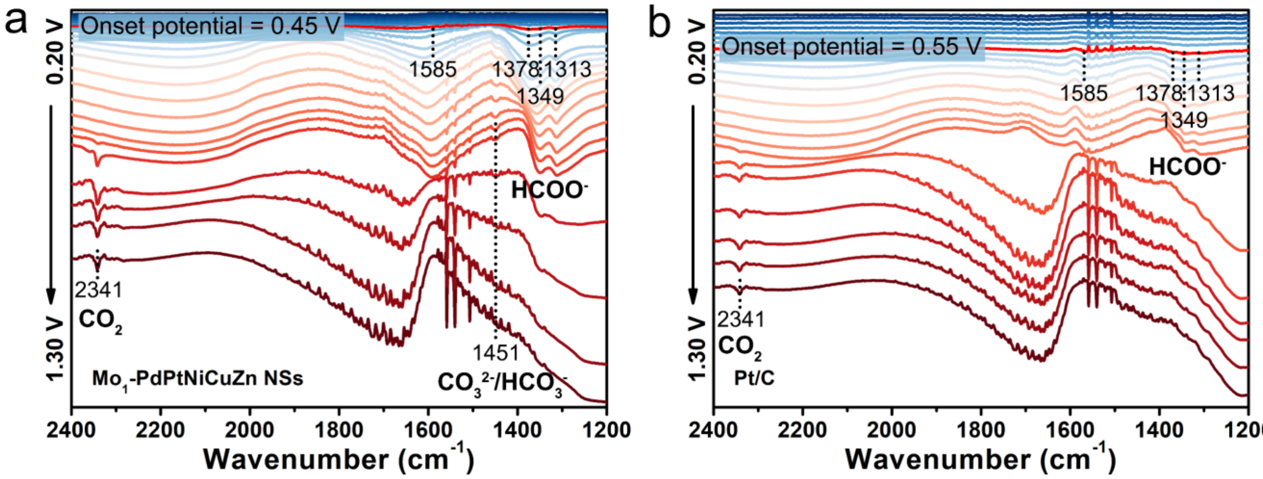

Figure R2. *In-situ* FTIR spectrum of MOR on (a) Mo_1-PdPtNiCuZn SAHEA nanosheets/C and (b) Pt/C at different potentials varying from 0.20 to 1.30 V at an interval of 0.05 V in 1.0 M KOH + 1.0 M methanol solution.

4. In Fig 5c, the Pt/C also shows the vibration peak corresponding to formate, and the intensity of peaks is comparable with those of Mo-PdPtNiCuZn. Doesn't this mean that Pt/C can also convert methanol to formate? An additional explanation for this should be provided.

Response: Thank you so much for your valuable comments. According to previous report [R3], methanol can be oxidized to CO_2 on pure Pt catalysts *via* a dual-path mechanism, including the CO pathway (CO_{ads} as the intermediate) and the formate pathway (formate as the intermediate), depending on the electrode potential. Therefore, commercial Pt/C can also partially oxidize methanol to formate. Unfortunately, the formation of strongly adsorbed CO_{ads} in the low overpotential leads to the self-poisoning of catalysts, resulting in deactivation. Herein, the planting of oxophilic Mo single atoms, the dilution of Pt-Pt ensembles *via* constructing high-entropy alloys, and intrinsic tensile strain enable Mo_1-PdPtNiCuZn SAHEA nanosheets to be an efficient MOR electrocatalyst to suppress the formation of CO_{ads} and promote the formate pathway.

In light of the reviewer's concern about the intensity of formate vibration peak on commercial Pt/C, we further provided the intensity changes of bands at 1349 cm^{-1} (ν_s (OCO) of formate) of Mo_1-PdPtNiCuZn SAHEA nanosheets and Pt/C with applied potentials (Fig. R3). Obviously, the formate characteristic band intensity of Mo_1-PdPtNiCuZn SAHEA nanosheets is much stronger than that of Pt/C, indicating that methanol can be more easily oxidized to formate on Mo_1-PdPtNiCuZn SAHEA nanosheets. Notably, the IR intensity of formate starts decreasing as early as 0.8 V on Mo_1-PdPtNiCuZn SAHEA nanosheets relative to Pt/C, implying the superior capacity to the deep oxidation of formate. In contrast, Pt/C severely suffers from the poisoning effect of CO_{ads}, resulting in poor MOR performance.

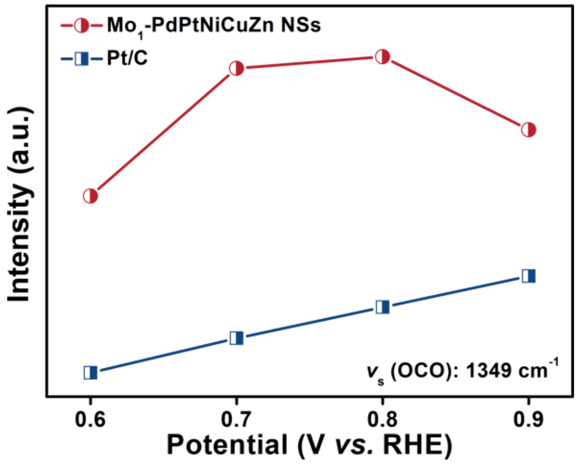

Figure R3. Dependence of IR intensity of ν_s (OCO) (1349 cm^{-1}) of formate on the electrode potential.

Reference:

[R3] Katayama, Yu. et al. Direct observation of surface-bound intermediates during methanol oxidation on platinum under alkaline conditions. *J. Phys. Chem. C* **125**, 26321-26331 (2021).

We have included the corresponding details in the revised manuscript.

"*The much stronger formate characteristic band (ν_s (OCO) of formate) on Mo_1-PdPtNiCuZn SAHEA NSs indicates that methanol can be more easily oxidized to formate on Mo_1-PdPtNiCuZn SAHEA NSs (Supplementary Fig. 29).*"

5. In Fig. 5d, the modeling of the reactant and product molecules needs to be clarified. They are hard to distinguish from the catalyst in the background. The Pt and Mo atoms also seem to be hard to find.

Response: Thank you so much for your thoughtful suggestions. We have redrawn this conceptual diagram in the revised manuscript. Particularly, we have clarified the distinction between species of reactant, product, and catalyst and replaced the colors of the individual atom models to facilitate identifying the Pt and Mo atoms (Fig. R4). Moreover, we have included a legend to improve the

identification of various species.

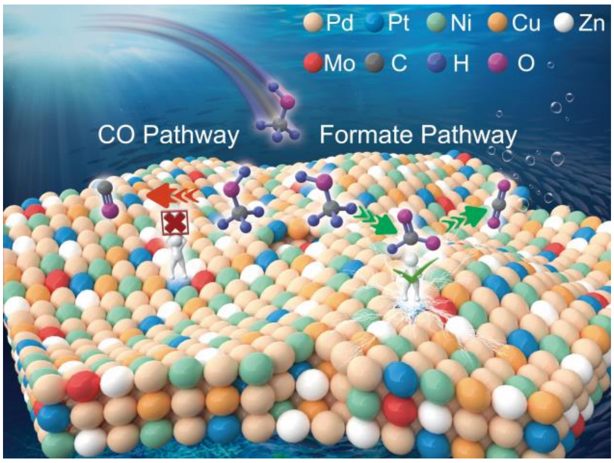

Figure R4. Schematic diagram of possible pathways for MOR on Mo_1-PdPtNiCuZn SAHEA nanosheets.

6. In Fig 6h, explain specifically for choosing U = 0.67 V.

Response: Thank you so much for your constructive comment. Theoretical calculations have verified that the rate-determining step (RDS) for the CO_2 pathway occurs at CHO* → HCOO*. At a potential of U = 0 V, Mo_1-PdPtNiCuZn exhibits a more energetically favorable pathway and lower overpotential (0.67 V) than PdPtNiCuZn. To make this result be more intuitive, an applied bias voltage of U = 0.67 V was applied for two curves to change the free energy reaction pathways, **which is the minimum applied voltage enabling to reach an ideal reaction equilibrium state at the RDS of CO_2 pathway without extra external energy on Mo_1-PdPtNiCuZn (111) surface**. In contrast, the PdPtNiCuZn (111) surface still manifests an uphill slope at the RDS at this voltage, indicating that additional energy is required, thus enabling a more visualized clarification of the superior MOR electrocatalytic performances on Mo_1-PdPtNiCuZn SAHEA nanosheets. In addition, we checked the theoretically calculated raw data (Table R1) and have corrected the graphical mistake in Fig. 6g, *i.e.*, the overpotentials of Mo_1-PdPtNiCuZn and PdPtNiCuZn at the RDS at U = 0 V are 0.67 and 1.11 V, respectively (Fig. R5).

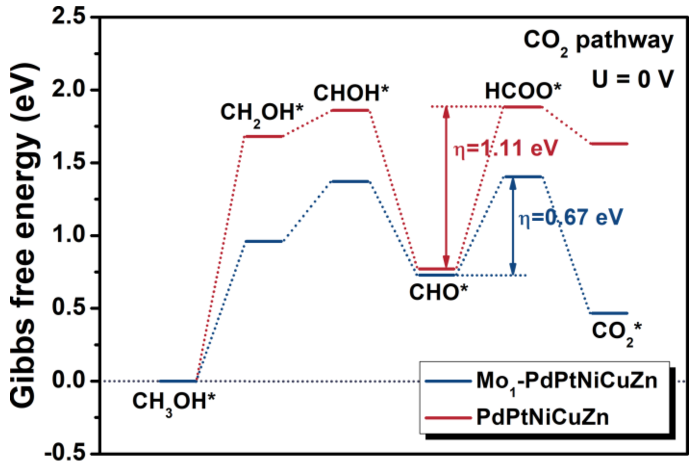

Figure R5. The reaction energy comparison of CO_2 pathway for MOR electrocatalysis on Mo_1-PdPtNiCuZn and PdPtNiCuZn.

Table R1. The theoretically calculated Gibbs free energy of CO_2 pathway for MOR electrocatalysis on Mo_1-PdPtNiCuZn and PdPtNiCuZn.

Adsorbed species	Gibbs free energy (eV)	
	Mo_1-PdPtNiCuZn	PdPtNiCuZn

CH_3OH*	0	0
CH_2OH*	0.96064	1.68172
CHOH*	1.37162	1.86049
**CHO***	**0.7284**	**0.77094**
**HCOO***	**1.40395**	**1.88336**
CO_2*	0.46702	1.63127

We have included the corresponding details in the revised manuscript.

"*At U = 0.67 V, the minimum applied voltage enabling to reach an ideal reaction equilibrium state at the RDS of CO_2 pathway without extra external energy on Mo_1-PdPtNiCuZn (111) surface [35], PdPtNiCuZn shows an uphill slope at the RDS, indicating that the reaction step is an endothermic reaction at this voltage and requires additional energy. In contrast, the ideal reaction equilibrium state is reached at the RDS without extra external energy on Mo_1-PdPtNiCuZn.*"

7. Explanation for the abbreviation SAHEA is absent.

Response: Thank you so much for bringing this to our attention. SAHEA is the abbreviation of single-atom high-entropy-alloy. We have included this full name in the revised manuscript.

"*An optimized well-defined Mo_1-PdPtNiCuZn single-atom high-entropy-alloy (SAHEA) NSs delivered an ultrahigh mass activity of 24.55 A mg_{Pt}^{-1} and 11.62 A mg_{Pd+Pt}^{-1}, respectively for MOR, 18.13 and 8.58 folds higher than that of Pt/C catalysts, with the impressive durability.*"

To Reviewer 2:

The manuscript presents the design of a novel class of efficient electrocatalysts: single-atom Mo-tailored PdPtNiCuZn high-entropy-alloy nanosheets. These nanosheets are specifically engineered to enhance the catalysis of the methanol oxidation reaction by promoting the formate pathway. While the authors have conducted experimental tests to investigate the reaction mechanism, certain aspects require further clarification.

Response: Thank you so much for professionally reviewing our manuscript and constructive comments to improve our manuscript. In line with your thoughtful suggestions, we supplemented extra data to make our results convincing. The point-by-point responses are listed below.

1. The authors have mentioned that their synthetic method for single-atom Mo-tailored HEA NSs with tensile-strained allows for the expansion of the compositional space of HEA hosts. However, it is important to provide a deeper explanation and supporting evidence for this claim. The essential reasons behind how this synthetic method enables the expansion of the compositional space of HEA hosts should be clearly stated.

Response: Thank you so much for your valuable comments. To expand the compositional space of HEA hosts, we chose some elements (M) that have the potential to react with Pd and Pt precursors. We found that these elements could be divided into two categories: ones that reduce faster (*e.g.*, Cu, Ag, Au) and others that reduce slower (*e.g.*, Rh, Fe, Co, Ni, Mn, Cr, Ir, Ru, Mo) (Fig. R6) [R4]. The elements with rapid nucleation rates need to be introduced into the PdPt nanosheet host *via* a template-directed strategy (*i.e.*, subsequent addition). However, other elements with slow reduction rates (*e.g.*, Rh, Fe, Co, Ni, Mn, Cr) could be gradually reduced at 170 °C under the effect of reductant (glucose) and then diffused into the PdPt host while reducing Ir, Ru, Mo, *etc.* always required a higher temperature (> 220 °C) [R4]. Given this, in our synthesis (the reaction temperature was 200 °C), the compositional space of HEA hosts was permitted to expand to include but not limited to senary (PdPtCoNiCuZn), septenary (PdPtFeCoNiCuZn) under the effect of reductant (L-ascorbic acid), where the pre-reduced Pt also had a certain catalytic effect on reducing other elements. However, a few reduced Mo atoms could be only planted into PdPt-based HEA hosts in the form of atomically dispersed sites, resulting in the single-atom Mo-tailored PdPt-based SAHEAs. In short, the growth for Mo-tailored SAHEA nanosheets can be divided into three major steps, involving the initial formation of PdPt-based nanosheet template, reduction and diffusion of other reducible atoms on the nanosheet template, and subsequent planting of atomically dispersed Mo sites.

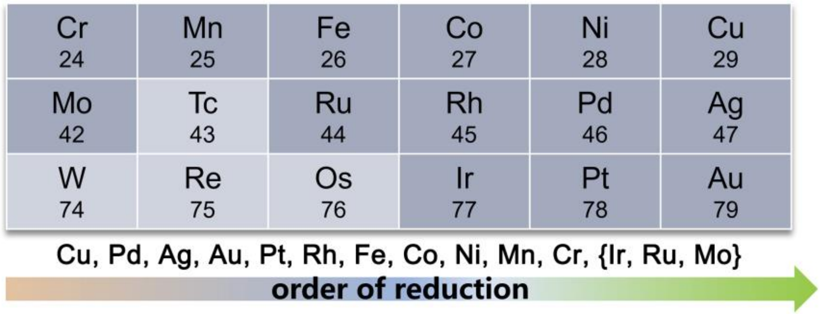

Figure R6. The choice of elements that have potential catalytic activity for HEAs [R8].

Reference:

[R4] Sun, Y. *et al.* A general approach to high-entropy metallic nanowire electrocatalysts. *Matter*, **6**, 193-205 (2023).

We have included the corresponding mechanism in the original manuscript. In addition, we also included a new indicative sentence to elicit the generality for the synthesis of SAHEA nanosheets.

"*Considering that other reducible elements (e.g., Fe, Co, Mn) can be alloyed with Pt in the presence of reductant in the OAm system at 200 °C [16], the present synthetic method for single-atom Mo-tailored HEA NSs with tensile-strained allows for the expansion of the compositional space of HEA hosts, such as senary Mo_1-PdPtCoNiCuZn SAHEA NSs (Fig. 3a and Supplementary Fig. 12) and septenary Mo_1-PdPtFeCoNiCuZn SAHEA NSs (Fig. 3b and Supplementary Fig. 13), etc.*"

2. The authors have highlighted the superiority of using PdPtNiCuZn high-entropy-alloy nanosheets as carriers for Mo single atoms compared to conventional carrier materials. It would be beneficial if the authors could supplement their claims by including data of conventional carrier materials tested under the same experimental conditions. This would provide a direct comparison and support their argument.

Response: Thank you so much for your thoughtful suggestion. In order to exclude your concerns, we have supplemented the additional experiments to confirm the merits of high-entropy alloy carriers. Specifically, the referenced single-atom Mo-tailored binary PdPt low-entropy-alloy nanosheets (Mo_1-PdPt) and single-atom Mo-tailored ternary PdPtNi medium-entropy-alloy nanosheets (Mo_1-PdPtNi) were synthesized *via* the similar method to that of quinary Mo_1-PdPtNiCuZn SAHEA nanosheets except for replacing the reactants with the corresponding metal precursors. The corresponding morphological, compositional, and structural characterizations demonstrate the successful construction of single-atom Mo-tailored low- and medium-entropy-alloy nanosheets featuring intrinsic tensile strain, lattice deformation, and defects (Fig. R7).

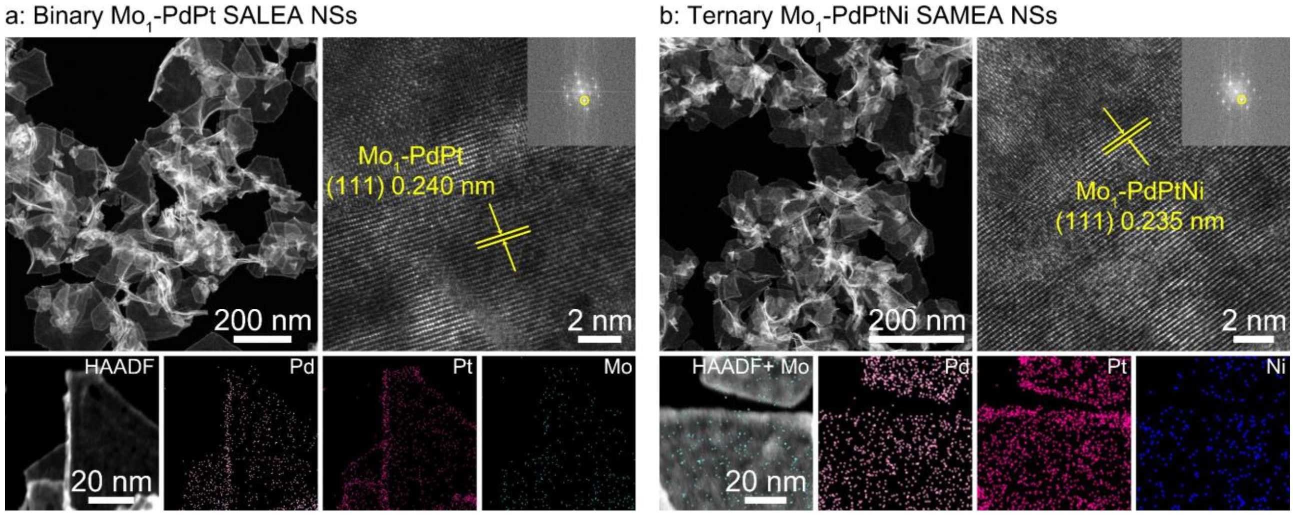

Figure R7. Morphological, compositional, and structural characterizations of Mo-tailored binary PdPt low-entropy-alloy and ternary PdPtNi medium-entropy-alloy nanosheets. (a) HAADF-STEM image, high-resolution HAADF-STEM image, FFT pattern, and HAADF-STEM-EDS elemental mapping of binary Mo_1-PdPt SALEA nanosheets. (b) HAADF-STEM image, high-resolution HAADF-STEM image, FFT pattern, and HAADF-STEM-EDS elemental mapping of ternary Mo_1-PdPtNi SAMEA nanosheets.

In addition, we further evaluated the MOR performances of the well-designed Mo_1-PdPt and Mo_1-PdPtNi nanosheets, which are further compared with the Mo_1-PdPtNiCuZn nanosheets and commercial Pt/C (Fig. R8). Although both Mo_1-PdPt and Mo_1-PdPtNi nanosheets show enhanced MOR activities compared to Pt/C, their catalytic performance is hardly comparable to Mo_1-PdPtNiCuZn SAHEA nanosheets due to the restricted electronic modulation and the inevitable formation of strong CO_{ads}. This directly demonstrates the superiority of using PdPtNiCuZn HEA nanosheets as carriers for Mo single-atom promoters, enabling the flexible and efficient modulation

of electrons and dilution of Pt-Pt ensembles to achieve superior MOR performances.

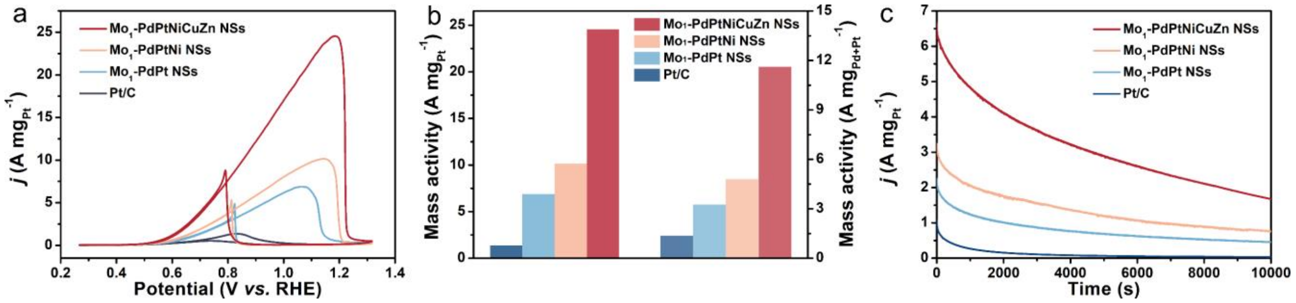

Figure R8. MOR performances of Mo_1-PdPtNiCuZn, Mo_1-PdPtNi, Mo_1-PdPt nanosheets, and Pt/C in alkaline electrolytes. (a) Pt mass-normalized MOR CV profiles. (b) MOR mass activity of Mo_1-PdPtNiCuZn SAHEA NSs, Mo_1-PdPtNi SAMEA NSs, Mo_1-PdPt SALEA NSs, and Pt/C in 1.0 M KOH containing 1.0 M methanol. (c) Chronoamperometric tests for MOR at 0.77 V *vs.* RHE.

We have included the corresponding details in the revised manuscript.

"*Moreover, the enhanced MOR electrocatalytic performance caused by the diluted Pt-Pt ensembles can be further confirmed by the fact that the Mo_1-PdPtNiCuZn SAHEA NSs catalysts exhibit significantly enhanced MOR performances over the single-atom Mo-tailored low- and medium-entropy-alloy NSs (Supplementary Fig. 26, 27).*"

3. The authors have proposed that dilute Pt-Pt ensembles and intrinsic tensile strain (Mo_1-PdPtNiCuZn) enhance the methanol oxidation reaction. However, it is important to provide relevant evidence to support this claim.

Response: We sincerely appreciate you for this constructive comment. In light of this well-intentioned suggestion, we have provided additional experiment evidence. Specifically, we employed CO stripping tests to investigate the adsorption behaviors of CO on the single-atom Mo-tailored low- and medium-entropy-alloy nanosheets with abundant interlinked Pt-Pt ensembles. The results reveal that methanol oxidation to CO_2 on these conventional alloy nanosheets remains *via* a dual-path mechanism (Fig. R9). The dramatically decreased CO_{ads} on Mo_1-PdPtNi SAMEA nanosheets relative to the massive formation of CO_{ads} on Mo_1-PdPt SALEA nanosheets and Pt/C proves that diluting Pt-Pt ensembles *via* introducing additional metal atoms can effectively reduce the formation of CO_{ads}. As expected, the oxidation peak of CO_{ads} is significantly suppressed on the Mo_1-PdPtNiCuZn SAHEA nanosheets, arising from the formation of isolated Pt sites on the surface without contiguous Pt atoms *via* constructing HEA to dilute Pt-Pt ensembles, logically leading to the reduction and even the elimination of CO adsorption and steer the MOR towards the formate dominated pathway.

It is noteworthy that the oxidation peaks of CO_{ads} on the single-atom Mo-tailored low- and medium-entropy-alloy nanosheets indicate apparent negative shifts, also verifying that the modification of oxophilic single-atom Mo promoters and ligand effect driven by the additional metal atoms can mitigate the CO poisoning effect *via* lowering the CO adsorption energy and efficiently providing OH_{ads} species to boost the oxidation of CO_{ads} to CO_2.

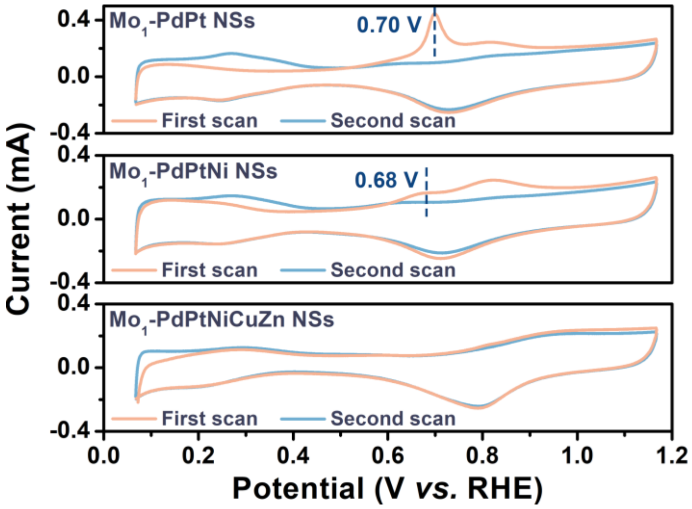

Figure R9. CO-stripping curves of Mo_1-PdPtNiCuZn SAHEA, Mo_1-PdPtNi SAMEA, Mo_1-PdPt SALEA nanosheets, and Pt/C catalysts recorded in N_2-saturated 1.0 M KOH.

We have included the corresponding details in the revised manuscript and Supplementary Information.

"*Specifically, the construction of Mo-tailored SAHEA NSs can significantly dilute Pt-Pt ensembles to achieve the slightest formation of CO_{ads} and optimize electronic structures to enhance the formate pathway (Supplementary Fig. 28).*"

"*The results reveal that methanol oxidation to CO_2 on these Mo-tailored LEA and MEA NSs remains through a dual-path mechanism. The oxidation peak of the absorbed CO on the Mo-tailored PdPt (Mo_1-PdPt) low-entropy-alloy (LEA) NSs is 0.70 V vs. RHE, negative shift of 50 mV relative to that of Pt/C (0.75 V vs. RHE), further indicating that the modification of single-atom Mo promoters and the introduction of intrinsic tensile strain can accelerate the further oxidation of CO intermediate. Of note, different from the massive formation of CO_{ads} on Mo_1-PdPt SALEA NSs and commercial Pt/C, CO_{ads} on Mo-tailored PdPtNi (Mo_1-PdPtNi) medium-entropy-alloy (MEA) NSs dramatically decreased. Meanwhile, the oxidation peak of the absorbed CO on Mo_1-PdPtNi SAMEA NSs negatively shifts to 0.68 V vs. RHE relative to that of Mo_1-PdPt SALEA NSs, confirming that the introduction of additional elements can dilute Pt-Pt ensembles, significantly reducing the formation of CO_{ads} and concurrently further accelerating the removal of CO_{ads}.*"

In addition, DFT calculations were performed to ascertain the tensile strain effect on MOR performance. The total *d*-density of states (TDOS) plots of different models were calculated to visualize the *d*-band centers, whose positions can be directly associated with enhanced MOR activity (Fig. R10a). Comparison with the electronic structure of Mo_1-PdPtNiCuZn reveals that the overall *d*-band center of strained Mo_1-PdPtNiCuZn has significantly shifted upward after introducing tensile strain, suggesting that the improved electron transfer efficiency of the electrocatalysts. Meanwhile, the upward shift of the *d*-band center enables stronger binding energy of strained Mo_1-PdPtNiCuZn to key reaction intermediates and *OH, thus increasing the chances of these intermediates being further oxidized to CO_2. Furthermore, we further studied the influence of tensile strain on reaction tendency at the rate-determining step (RDS). The more negative ΔG value of the RDS on strained Mo_1-PdPtNiCuZn confirmed that the intrinsic tensile strain can achieve a more energetically favorable pathway and minimize the MOR barrier, further improving the MOR performance (Fig. R10b).

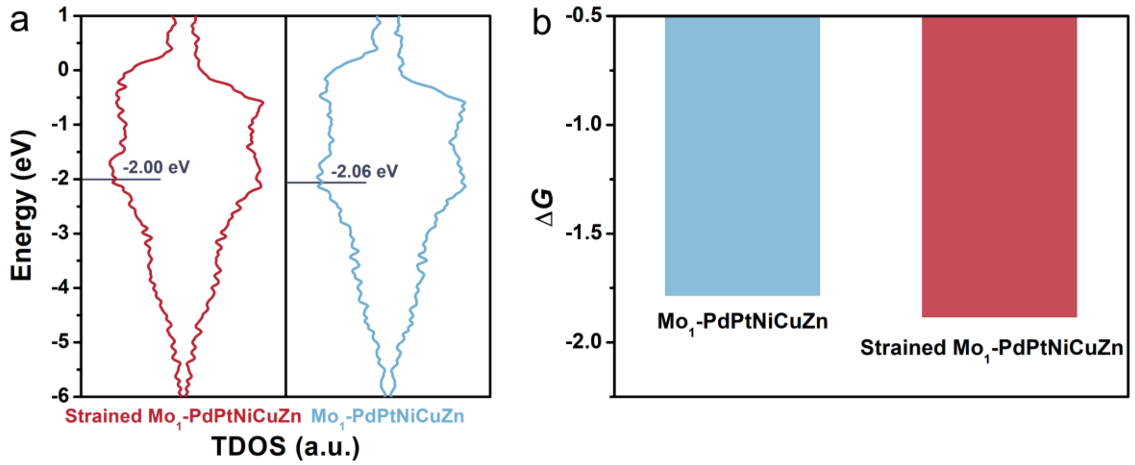

Figure R10. (a) The TDOS of *d*-band in strained Mo_1-PdPtNiCuZn and Mo_1-PdPtNiCuZn models. (b) The ΔG of RDS on strained Mo_1-PdPtNiCuZn and Mo_1-PdPtNiCuZn.

All in all, significantly diluted Pt-Pt ensembles, along with the single-atom Mo-tailored configuration and intrinsic tensile strain, coherently endow the well-organized Mo_1-PdPtNiCuZn SAHEA nanosheets with satisfactory MOR kinetics, logically manipulating their excellent activity and long-term durability.

4. The manuscript does not clearly address the stability of the single-atom Mo-tailored PdPtNiCuZn high-entropy-alloy nanosheets. It is crucial to discuss the stability of these nanosheets and provide evidence, such as long-term stability tests or characterization data.

Response: Thank you so much for your constructive suggestion. We have additionally tested the long-term stability of Mo_1-PdPtNiCuZn SAHEA nanosheets towards MOR electrocatalysis by chronoamperometric tests (Fig. R11), specifically highlighting the impressive long-term durability. We also found that the well-designed Mo_1-PdPtNiCuZn SAHEA nanosheets exhibit excellent long-term durability towards MOR electrocatalysis. We have included the corresponding data in the revised Supporting Information and the corresponding explanation in the revised manuscript.

"*Additionally, CA tests of the Mo_1-PdPtNiCuZn SAHEA NSs for MOR have been further prolonged to 10 h (Supplementary Fig. 21). The current densities remain 1.15 A mg_{Pt}^{-1} for MOR after the long-term durability tests, further confirming the superior stability of Mo_1-PdPtNiCuZn SAHEA NSs.*"

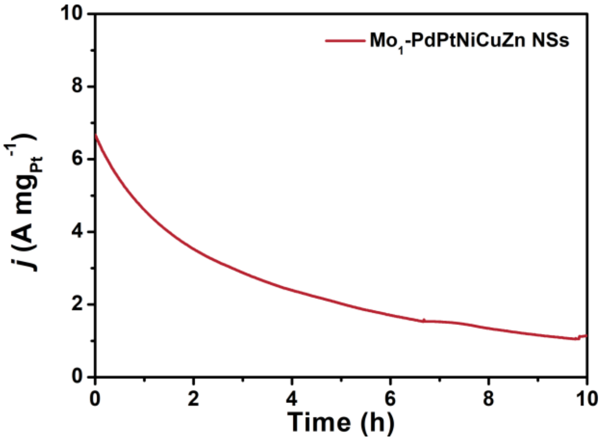

Figure R11. Chronoamperometric tests of Mo_1-PdPtNiCuZn SAHEA nanosheets for MOR at 0.77 V *vs.* RHE.

To Reviewer 3:

I have reviewed the submitted manuscript thoroughly and, regrettably, I must recommend against its publication. This paper reported a class of single-atom Mo-tailored PdPtNiCuZn high-entropy-alloy (HEA) nanosheets for enhancing the methanol oxidation reaction (MOR) electrocatalysis. EDS mapping is used multiple times throughout the whole manuscript to demonstrate the single atom Mo on the HEAs. However, due to the poor resolution of the results, all these EDS results cannot prove that Mo exists as a single atom at all. Therefore, the experimental results cannot support the statements at all. Moreover, HEA electrocatalysts have been extensively studied and the study lacks novelty. Since the lack of novelty, substandard visual presentation, and unconvincing experimental results, I am constrained to recommend the publication of this manuscript. My evaluation is based on several notable issues that are outlined below.

Response: Thank you so much for your great efforts and valuable time in reviewing our manuscript. We sincerely appreciate your professional comments, which greatly help further improve the quality of our manuscript.

In light of your concern that EDS with poor resolution is insufficient to prove that Mo exists in monatomic form, we have performed extensive characterizations and analyses, such as XAFS, HAADF-STEM, atomic-level EELS, and ICP-MS, to confirm that the Mo species are immobilized on all the Mo-tailored HEAs in the form of atomically dispersed sites. The specific changes are as follows:

1. High-resolution HAADF-STEM-EDS were utilized to verify the impressive structural and compositional stability of these tailored Mo_1-PdPtNiCuZn SAHEA nanosheets.

2. Atomic-level EELS spectra further prove that the Mo species in Mo_1-PdPtNiCuZn SAHEA nanosheets mainly exist in the form of single atoms.

3. XAFS confirms that the Mo species are immobilized on these Mo-tailored HEA nanosheets and nanoparticles in the form of atomically dispersed sites without long-range coordination to other Mo metal centres (absences of Mo-Mo and Mo-O-Mo scatterings).

4. Employing the ICP-MS to demonstrate the accuracy and usability of ICP-OES results in this study.

In addition, based on the concerns about the novelty of our work from the reviewer, we would like to further emphasize the innovation and scientific breakthrough in details in the revised manuscript. The novelty of this work has become more distinct, as depicted in the following:

• While tuning the electronic structure of Pt *via* alloying can thermodynamically alleviate the CO poisoning of conventional Pt-based catalysts in direct methanol fuel cells, the effective modulation of the electronic structure is impeded by the limited compositional space, inevitably suffering from the poisoning effect of CO_{ads}. HEAs are promising materials for the freedom from CO poisoning and improving MOR performance because of the expansive and modulable compositional space, flexible and efficient electronic structure modulation, and significant dilution of Pt-Pt ensembles. However, precise design and optimization of HEAs at the atomic level for greatly enhancing the activity, CO tolerance, and stability for MOR is still a grand challenge. This study aims to fill this knowledge gap.

• Single-atom promoters can serve as co-catalysts to effectively modulate the *d*-band center of their adjacent sites, further accelerating catalytic reaction kinetics. However, owing to the different reduction kinetics of various metal precursors and the complexity of crystal growth, achieving precise structural integration of single-atom and HEA features in a system to promote energy electrocatalysis remains a grand challenge. In this study, we precisely constructed a class of

single-atom Mo-tailored HEA nanosheets with dilute Pt-Pt ensembles and intrinsic tensile strain as efficient electrocatalysts for enhancing the MOR catalysis for the first time *via* selective reduction-diffusion engineering. We also demonstrated the preparation method is highly general for the various single-atom Mo-tailored HEA nanosheets.

• The complex atomic structure and elemental composition of HEAs make it hard to dig out the real structure-activity relationship. In this study, we successfully revealed the origin of the high MOR performance of Mo_1-PdPtNiCuZn SAHEA nanosheets through in situ spectroscopy and theoretical calculations. We found that the significantly diluted Pt-Pt ensembles can effectively reduce the formation of CO_{ads}, the electronic structure of isolated Pt sites and the adsorption behavior of intermediates can be further optimized by oxophilic single-atom Mo promoters, enabling the inhibition of the CO pathway and concurrent enhancement of the formate pathway. In addition, we further demonstrate that the intrinsic tensile strain is also beneficial for minimizing the MOR barrier and accelerating MOR kinetics.

Additionally, the well-designed Mo_1-PdPtNiCuZn SAHEA nanosheets catalysts deliver the extraordinary MOR mass activities of 24.55 A mg_{P}^{t-1} and 11.62 A mg_{Pd+Pt}^{-1} and specific activity of 16.55 mA cm^{-2}, 18.13, 8.58, and 7.13 times higher than those of commercial Pt/C, respectively, along with impressive long-term durability. The comparison with state-of-the-art Pd- and Pt-based catalysts was supplemented in Supplementary Table 3 with a further discussion in the revised manuscript.

We have emphasized the innovation and scientific breakthrough of this study in the revised manuscript and undertaken extensive revisions to make our results more convincing. We sincerely hope that the revised manuscript can address your concerns and will satisfy your stringent criteria for the publication of ***Nature Communications.*** Our point-by-point responses to the comments are as follows.

1. The noble metals chosen in the alloy turn out the studied material less competitive in comparison with those noble less high-entropy alloys.

Response: Thank you so much for your professional comment. It is well known that Pt/Pd-based materials are the most intensively studied MOR electrocatalysts with low overpotentials for initiating reactions and superior catalytic performance. However, their high dosage and poor tolerance to CO are inevitable barriers to large-scale commercial applications. Alloying Pt/Pd with one or more oxophilic elements, such as Ru, Ni, Co, and Cu, is one of the most conventional and effective method to improve their MOR activity. However, the effective modulation of the electronic structure is impeded by the limited compositional space, making it challenging to maximize the catalytic performance. Therefore, PtPd-based HEA nanosheets enabling flexible and efficient electronic structure modulation have been chosen as the basic catalysts for undertaking study in this system.

Although the HEA system in this study contains two noble metals (Pd and Pt), they can be diluted by other transition metals and dispersed uniformly throughout the ultrathin nanosheets, enabling the maximum exposure of the noble metals. In addition, the introduction of oxophilic single-atom Mo promoters can drive the further optimization of the electronic structure of these dilute active sites and improvement of the adsorption behaviors of intermediates, enabling the inhibition of the CO pathway and concurrent enhancement of the formate pathway. The intrinsic tensile strain induced by the wavy suprathin structure facilitates minimizing the MOR barrier and accelerating MOR kinetics. These advantageous structures confer Mo_1-PdPtNiCuZn SAHEA nanosheets ultra-high specific and mass activities. In order to highlight the advantages of this Mo_1-PdPtNiCuZn SAHEA system, we further compared the MOR performance with previous state-of-the-art Pd- and Pt-based electrocatalysts in alkaline conditions (Table R2). The optimized Mo_1-PdPtNiCuZn SAHEA nanosheets deliver

extraordinary specific and mass activities of 16.55 mA cm^{-2}, 24.55 A mg_{Pt}^{-1}, and 11.62 A mg_{Pd+Pt}^{-1}, obviously higher than most of reported state-of-the-art Pd- and Pt-based electrocatalysts, reflecting the stronger competition of Mo_1-PdPtNiCuZn SAHEA nanosheets and their potential applications towards the next-generation low-cost and high-performance fuel cells.

Table R2. The performance comparison of the Mo_1-PdPtNiCuZn SAHEA nanosheets with previous state-of-the-art Pd- and Pt-based electrocatalysts towards MOR electrocatalysis.

Catalysts	Pt-mass-normalized activity (A mg_{Pt}^{-1})	Noble-metal-mass-normalized activity (A mg_{PGM}^{-1})	ECSA-normalized activity (mA cm^{-2})	Electrolytes	References
**Mo_1-PdPtNiCuZn SAHEA nanosheets**	**24.55**	**11.62**	**16.55**	**1.0 M KOH + 1.0 M methanol**	**This work**
PtNiGaSnMoRe HEA senary nanowires	6.20	-	12.31	1.0 M KOH + 1.0 M methanol	R5
$Pt_{18}Ni_{26}Fe_{15}Co_{14}Cu_{27}$ HEA nanoparticles	15.04	-	-	1.0 M KOH + 1.0 M methanol	R6
PdAgSn/PtBi HEA nanoparticles	-	2.87	4.67	1.0 M KOH + 1.0 M methanol	R7
PdPt-based HEA-nanoparticles-(14)	12.6	-	-	1.0 M KOH + 1.0 M methanol	R8
Pt clusters/$Ti_3C_2T_x$	7.32	-	-	1.0 M KOH + 1.0 M methanol	R9
Au@PdPt	-	4.83	6.90	1.0 M KOH + 1.0 M methanol	R10
Au@Pt_1-Pd_1 hemispherical nanostructures	-	4.38	5.04	1.0 M KOH + 1.0 M methanol	R11
o-$PdH_{0.43}$@Pt	32.51	3.68	-	1.0 M KOH + 1.0 M methanol	R12
Pt_5Ce/C	9.13	-	32.74	1.0 M KOH + 1.0 M methanol	R13
PtCo nanoframes	4.28	-	8.56	1.0 M KOH + 1.0 M methanol	R14
Pt/N-doped graphdiyne (Pt^0 atoms)	1.45	-	-	1.0 M KOH + 1.0 M methanol	R15
SA Ni-Pt nanowires	7.93	-	7.48	1.0 M KOH + 1.0 M methanol	R16
PtRhBiSnSb HEA nanoplates	-	19.53	-	1.0 M KOH + 1.0 M methanol	R17
$Pd_3Pb/Pt_{2.37}Pb$ nanocubes	8.4	-	13.39	1.0 M KOH + 1.0 M methanol	R18
NiPtSAA/graphdiyne	4.38	-	-	1.0 M KOH + 1.0 M methanol	R19
$PdSn_{0.5}$/Se-Ti_3C_2	-	4.76	6.25	1.0 M NaOH + 1.0 M methanol	R20
$Pd_{(20\%)}$-$CeO_{2(20\%)}$/nitrogen-doped mesoporous carbon sphere	-	1.50	-	1.0 M KOH + 1.0 M methanol	R21
Pt_1/RuO_2	6.77	-	-	0.1 M KOH + 1.0 M methanol	R22
PtCuPd@Ru yolk-cage nanostructures	-	2.48	-	1.0 M KOH + 2.0 M methanol	R23

PtZn intermetallic nanoparticles	0.58	-	1.14	0.1 M KOH + 0.5 M methanol	R24
Pt/Ni$(OH)_2$/reduced graphene oxide	1.23	-	1.93	1.0 M KOH + 1.0 M methanol	R25

We have included this Table in the revised Supplementary Information.

References:

[R5] Yang, F. *et al.* Ultrathin PtNiGaSnMoRe senary nanowires with partial amorphous structure enable remarkable methanol oxidation electrocatalysis. *Adv. Energy Mater.* **13**, 2301408 (2023).
[R6] Li, H. *et al.* Fast site-to-site electron transfer of high-entropy alloy nanocatalyst driving redox electrocatalysis. *Nat. Commun.* **11**, 5437 (2020).
[R7] Lao, X. *et al.* Pd-enriched-core/Pt-enriched-shell high-entropy alloy with face-centred cubic structure for C_1 and C_2 alcohol oxidation. *Angew. Chem. Int. Ed.* **62**, e202304510 (2023).
[R8] Wang, Y. *et al.* Synthesis of high-entropy-alloy nanoparticles by a step-alloying strategy as a superior multifunctional electrocatalyst. *Adv. Mater.* **35**, 2302499 (2023).
[R9] Zhu, J. *et al.* Ultrahigh stable methanol oxidation enabled by a high hydroxyl concentration on Pt clusters/MXene interfaces. *J. Am. Chem. Soc.* **144**, 15529-15538 (2022).
[R10] Yang, X. *et al.* Modulating electronic structure of an Au-nanorod-core-PdPt-alloy-shell catalyst for efficient alcohol electro-oxidation. *Adv. Energy Mater.* **11**, 2100812 (2021).
[R11] Liang, W. *et al.* 3D anisotropic Au@Pt-Pd hemispherical nanostructures as efficient electrocatalysts for methanol, ethanol, and formic acid oxidation reaction. *Adv. Mater.* **33**, 2100713 (2021).
[R12] Liu, G. *et al.* Hydrogen-intercalation-induced lattice expansion of Pd@Pt core-shell nanoparticles for highly efficient electrocatalytic alcohol oxidation. *J. Am. Chem. Soc.* **143**, 11262-11270 (2021).
[R13] Zhang, S. *et al.* Lanthanide electronic perturbation in Pt-Ln (La, Ce, Pr and Nd) alloys for enhanced methanol oxidation reaction activity. *Energ. Environ. Sci.* 14, 5911-5918 (2021).
[R14] Chen, S. *et al.* High-performance Pt-Co nanoframes for fuel-cell electrocatalysis. *Nano Lett.* **20**, 1974-1979 (2020).
[R15] Hui, L. *et al.* Highly loaded independent Pt^0 atoms on graphdiyne for pH-general methanol oxidation reaction. *Adv. Sci.* **9**, 2104991 (2022).
[R16] Li, M. *et al.* Single-atom tailoring of platinum nanocatalysts for high-performance multifunctional electrocatalysis. *Nat. Catal.* **2**, 495-503 (2019).
[R17] Chen, W. *et al.* High-entropy intermetallic PtRhBiSnSb nanoplates for highly efficient alcohol oxidation electrocatalysis. *Adv. Mater.* 34, 2206276 (2022).
[R18] Wu, X. *et al.* Tuning surface structure of Pd_3Pb/Pt_nPb nanocrystals for boosting the methanol oxidation reaction. *Adv. Sci.* **6**, 1902249 (2019).
[R19] Hui, L. *et al.* Atomic alloys of nickel-platinum on carbon network for methanol oxidation. *Nano Energy* **95**, 106984 (2022).
[R20] Chen, S. *et al.* Engineering support and distribution of Palladium and Tin on MXene with modulation of the *d*-band center for CO-resilient methanol oxidation. *Angew. Chem. Int. Ed.* **61**, e202209693 (2022).
[R21] Tan, Q. *et al.* Highly dispersed Pd-CeO_2 nanoparticles supported on N-doped core-shell structured mesoporous carbon for methanol oxidation in alkaline media. *ACS Catal.* **9**, 6362-6371 (2019).
[R22] Zhang, Z. *et al.* Single-atom catalyst for high-performance methanol oxidation. *Nat. Commun.* **12**, 5235 (2021).
[R23] Saleem, F. *et al.* Elemental segregation in multimetallic core-shell nanoplates. *J. Am. Chem. Soc.* **141**, 14496-14500 (2019).

[R24] Qi, Z. *et al.* Sub-4 nm PtZn intermetallic nanoparticles for enhanced mass and specific activities in catalytic electrooxidation reaction. *J. Am. Chem. Soc.* **139**, 4762-4768 (2017).
[R25] Huang, W. *et al.* Highly active and durable methanol oxidation electrocatalyst based on the synergy of platinum-nickel hydroxide-graphene. *Nat. Commun.* **6**, 10035 (2015).

2. ICP-OES shouldn't be used to detect single atom Mo accurately since ICP-OES is not sensitive to low concentration.

Response: Thank you so much for pointing this out. We agree that the sensitivity of ICP-OES is not enough to detect the existence of low-concentration Mo atoms. However, by comparison, inductively coupled plasma mass spectrometry (ICP-MS) with an impressive detection limit (mostly at part *per* trillion (ppt) scale) can be used to detect extremely low concentrations of metals. In response to this comment, we employed ICP-MS to re-detect the concentration of each atom in Mo_1-PdPtNiCuZn SAHEA nanosheets (Table R3). The atomic ratio of Pd/Pt/Ni/Cu/Zn/Mo is determined to be 24.1/14.6/26.3/22.1/11.1/1.8, in well agreement with the ICP-OES result, further implying the accuracy and usability of ICP-OES results in our work.

We have included the corresponding details in the revised manuscript.

"*The atomic ratio of Pd/Pt/Ni/Cu/Zn/Mo in Mo_1-PdPtNiCuZn SAHEA NSs is determined to be 24.1/14.6/26.3/22.1/11.1/1.8 by inductively coupled plasma mass spectrometry (ICP-MS), in well agreement with the STEM energy-dispersive X-ray spectroscopy (STEM-EDS) and inductively coupled plasma optical emission spectroscopy (ICP-OES) results (Fig. 1d and Supplementary Table 1).*"

In addition, we have included the instrument model of ICP-MS in the Experimental Sections in the revised Supplementary Information.

"*The compositions were analyzed by inductively coupled plasma optical emission spectroscopy (ICP-OES, Optima 8300) and inductively coupled plasma mass spectrometry (ICP-MS, Agilent 7800).*"

Table R3. Atomic ratio of Pd/Pt/Ni/Cu/Zn/Mo in Mo_1-PdPtNiCuZn SAHEA nanosheets detected by ICP-MS, ICP-OES, and STEM-EDS tests.

Detected methods	Pd	Pt	Ni	Cu	Zn	Mo
ICP-MS	24.1	14.6	26.3	22.1	11.1	1.8
ICP-OES	26.9	13.3	24.8	21.9	11.0	2.1
STEM-EDS	25.8	12.3	24.7	22.3	12.2	2.7

3. Provide evidence that concaves defects mainly arise from oxidative etching by a trace amount of O_2 introduced during the addition of Cu precursors (Fig. 1g).

Response: Thank you so much for your constructive comment. In response to this issue, we have supplemented an additional controlled experiment. Specifically, we dissolved the $Cu(acac)_2$ into O_2-saturated 1.0 mL OAm and then added it dropwise to the reaction solution under magnetic stirring at 200 °C. The result revealed that only pierced Mo_1-PdPtNiCuZn SAHEA nanosheets could be obtained when more O_2 was introduced into the Cu precursor solution, indicating that excessive O_2 could generate enhanced oxidative etching, further implying that concave defects mainly arose from oxidative etching by a trace amount of O_2 introduced during the addition of Cu precursors (Fig. R12).

We have included this content in the revised manuscript.

“This can be confirmed by the fact that only pierced Mo_1-PdPtNiCuZn SAHEA NSs were obtained when introducing more O_2 into the Cu precursor solution (Supplementary Fig. 6).”

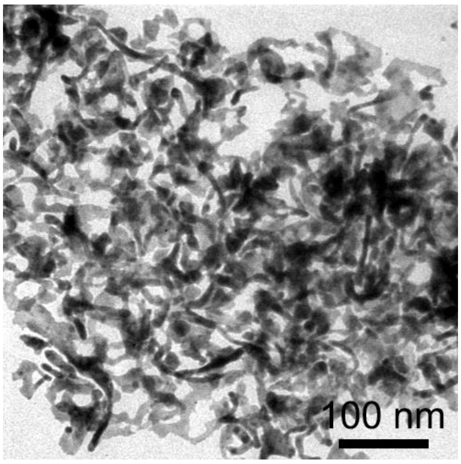

Figure R12. TEM image of the products with the same reaction conditions as those of Mo_1-PdPtNiCuZn SAHEA nanosheets except for introducing more O_2 into the Cu precursor solution.

4. How 4.4% tensile strain is calculated? Is this the strain of the overall sample? Localized d-spacing can’t prove strain for the whole sample since it is well known that high-entropy alloys are not that uniform in terms of the composition from region to region. Provide evidence for the strain.

Response: Thank you so much for your professional comments. In the original manuscript, the ~4.4% intrinsic tensile strain induced by the 2D wavy suprathin nanosheet structure was calculated from the following equation: $s(\mathrm{SAHEA}) = (d_{\mathrm{SAHEA}} - d_{\mathrm{bulk}})/d_{\mathrm{bulk}}$, where d_{SAHEA} and d_{bulk} represent the lattice constants of Mo_1-PdPtNiCuZn SAHEA nanosheets (measured to be 0.235 nm *via* the aberration-corrected HAADF-STEM of single Mo_1-PdPtNiCuZn SAHEA nanosheet (Fig. R13a)) and PdPt bulk (0.225 nm), respectively. To make this value statistically significant, we have determined the average lattice spacing of multiple Mo_1-PdPtNiCuZn SAHEA nanosheets to be 0.235 nm (Fig. R13b-d).

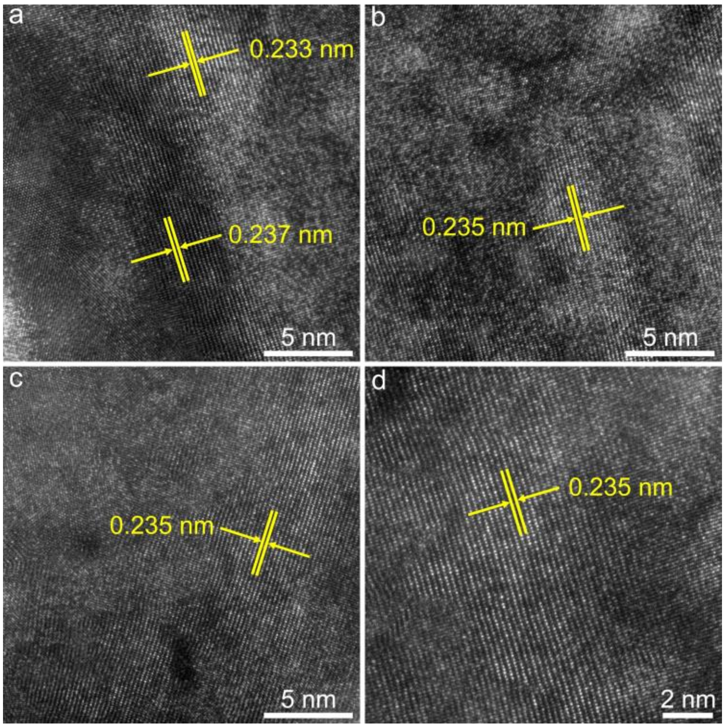

Figure R13. High-resolution HAADF-STEM images of the surface atomic arrangement on multiple

Mo_1-PdPtNiCuZn SAHEA nanosheets.

We agree that this tensile strain is localized rather than the overall sample. Therefore, in order to precisely ascertain the strain of the overall Mo_1-PdPtNiCuZn SAHEA nanosheets, we have calculated the lattice constant of MoPdPtNiCuZn solid solution bulk (S-bulk) with the same composition as Mo_1-PdPtNiCuZn SAHEA NSs (Pd/Pt/Ni/Cu/Zn/Mo = 26.9/13.3/24.8/21.9/11.0/2.1) *via* Vegard's law to further calculate the strain of the overall sample [R26]. Specifically, the lattice constant of $Mo_{2.1}Pd_{26.9}Pt_{13.3}Ni_{24.8}Cu_{21.9}Zn_{11.0}$ bulk can be calculated to be 0.2144 nm by the following equation: $d_{S\text{-}bulk} = 0.269d_{Pd} + 0.133d_{Pt} + 0.248d_{Ni} + 0.219d_{Cu} + 0.110d_{Zn} + 0.021d_{Mo}$, where $d_{S\text{-}bulk}$, d_{Pd}, d_{Pt}, d_{Ni}, d_{Cu}, d_{Zn}, and d_{Mo} represent the lattice constants of $Mo_{2.1}Pd_{26.9}Pt_{13.3}Ni_{24.8}Cu_{21.9}Zn_{11.0}$ S-bulk, Pd bulk, Pt bulk, Ni bulk, Cu bulk, Zn bulk, and Mo bulk, respectively (Table R4). Furthermore, the overall lattice constant of Mo_1-PdPtNiCuZn SAHEA nanosheets can be determined to be 0.2238 nm according to the X-ray diffraction (XRD) pattern of Mo_1-PdPtNiCuZn SAHEA nanosheets (Fig. R14). Therefore, the tensile strain of the overall Mo_1-PdPtNiCuZn SAHEA nanosheets can be calculated to be ~4.4%, according to equation: $s(\text{O-SAHEA}) = (d_{O\text{-}SAHEA} - d_{S\text{-}bulk})/d_{S\text{-}bulk}$, further demonstrating that the validity and usability of the tensile strain measurements in our work.

Table R4. Lattice constants of various metal bulks.

Bulk	Pd	Pt	Ni	Cu	Zn	Mo
Lattice constant (*d*, Å)	2.246	2.265	2.034	2.088	2.091	2.225

Notably, XRD patterns also showed that the diffraction peaks of Mo_1-PdPtNiCuZn SAHEA nanosheets were significantly shifted to smaller angles (Fig. R14), indicating their enlarged lattices, thus delivering a more serious tensile strain compared to Mo_1-PdPtNiCuZn SAHEA nanoparticles.

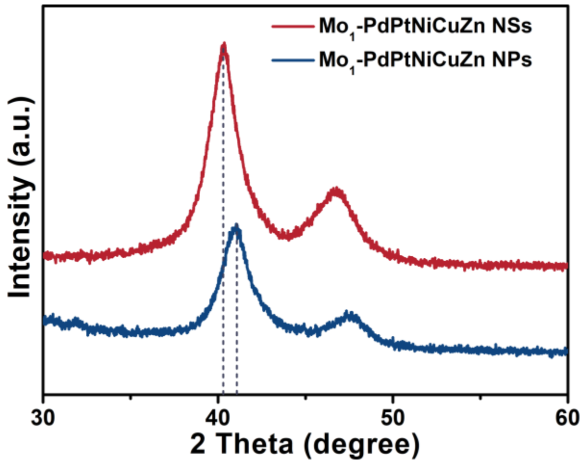

Figure R14. XRD patterns of Mo_1-PdPtNiCuZn SAHEA nanosheets and Mo_1-PdPtNiCuZn SAHEA nanoparticles.

We have included the corresponding details in the revised Supplementary Information.

"*The calculation of localized intrinsic tensile strain follows the equation below: s(SAHEA) = (d_{SAHEA} - d_{bulk})/d_{bulk}, where d_{SAHEA} and d_{bulk} represent the lattice constant of Mo_1-PdPtNiCuZn SAHEA NSs (measured to be 0.235 nm via the aberration-corrected HAADF-STEM) and PdPt bulk (0.225 nm), respectively. In addition, the overall tensile strain can be determined by Vegard's law and XRD pattern. Specifically, the lattice constant of MoPdPtNiCuZn solid solution bulk (S-bulk) with the same composition as Mo_1-PdPtNiCuZn SAHEA NSs (Pd/Pt/Ni/Cu/Zn/Mo = 26.9/13.3/24.8/21.9/11.0/2.1) can be calculated to be 0.2144 nm according to Vegard's law and the lattice constant of the overall Mo_1-PdPtNiCuZn SAHEA nanosheets can be determined to be 0.2238 nm according to the XRD pattern of Mo_1-PdPtNiCuZn SAHEA nanosheets. Therefore, the tensile strain of the overall Mo_1-PdPtNiCuZn SAHEA nanosheets can be calculated to be ~4.4%, according*

to the equation: s(O-SAHEA) = ($d_{O\text{-}SAHEA}$ - $d_{S\text{-}bulk}$)/$d_{S\text{-}bulk}$. These results reveal that the 2D wavy suprathin nanosheet structure can induce the accommodated intrinsic tensile strain of ~4.4%."

Reference:

[R26] Denton, A. & Ashcroft, N. Vegard's law. *Phys. Rev. A* **43**, 3161 (1991).

5. EDS mappings through the whole manuscript can't prove that Mo atoms exist as single atoms given its high noise ratio and poor resolution. Again, given the highly noisy nature of EDS, it can't be used to demonstrate that Mo exists as single atoms on other HEA NSs. For example, in Figure S17, if Mo exists as single atoms, then Zn should also exist as single atoms given their similar results.

Response: Thank you so much for your constructive comments. We agree that the poor resolution of EDS mapping is not sufficient to depict that Mo atoms exist as single atoms. Therefore, we employed the Fourier transforms of the extended X-ray absorption fine structure (FT-EXAFS) spectroscopy in the original manuscript to further confirm the existence of single Mo atoms in Mo_1-PdPtNiCuZn SAHEA nanosheets (Fig. 2e).

In order to further confirm the existence of single Mo atoms in other Mo-tailored HEA nanosheets (senary Mo_1-PdPtCoNiCuZn and septenary Mo_1-PdPtFeCoNiCuZn), the FT-EXAFS were additionally employed. The k^3-weight FT-EXAFS spectra reveal that both Mo_1-PdPtCoNiCuZn and Mo_1-PdPtFeCoNiCuZn SAHEA nanosheets exhibit two characteristic peaks at ~ 1.66 Å and ~ 2.58 Å, attributed to Mo-O and Mo-M (M = Pd/Pt/(Fe)/Co/Ni/Cu/Zn) scatterings, respectively (Fig. R15). Remarkably, the characteristic peaks of Mo-Mo scattering (~ 2.34 and Å for the Mo foil) and Mo-O-Mo scattering (~ 3.19 Å for the MoO_3) are almost absent, strongly confirming that the Mo species are immobilized on these Mo-tailored HEA nanosheets in the form of atomically dispersed sites without long-range coordination to other Mo metal centres. These results demonstrate the successful construction of single-atom Mo-tailored PdPt-based SAHEA nanosheets and the generality of the present synthetic method for single-atom Mo-tailored SAHEA nanosheets.

We have included the corresponding details in the revised manuscript.

"*The k^3-weight FT-EXAFS spectra further verify the single-atom feature of Mo species (Supplementary Fig. 14).*"

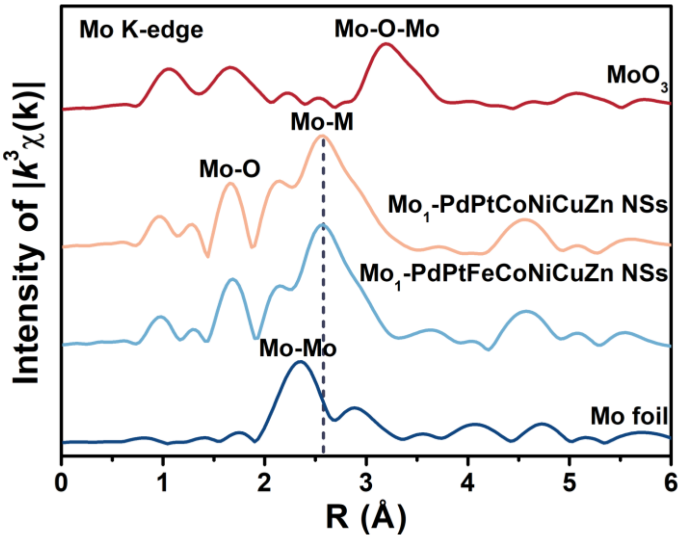

Figure R15. k^3-weighted Fourier transformation of the Mo K-edge EXAFS spectra.

In addition, Supplementary Fig. 17 depicted the morphological, structural, and compositional information of Mo_1-PdPtNiCuZn SAHEA nanosheets after MOR durability tests. However, the poor resolution of ordinary TEM-EDS mapping has raised your misgivings about Mo exists as single atoms. Therefore, we have reanalyzed the composition of Mo_1-PdPtNiCuZn SAHEA nanosheets after MOR durability tests by employing the high-resolution HAADF-STEM-EDS (Fig. R16). The very similar compositional distributions before and after durability tests confirm the impressive structural and compositional stability of Mo_1-PdPtNiCuZn SAHEA nanosheets, enabling the retention of single-atom Mo-tailored HEA structures after durability tests. We have updated the figures in the revised Supplementary Information.

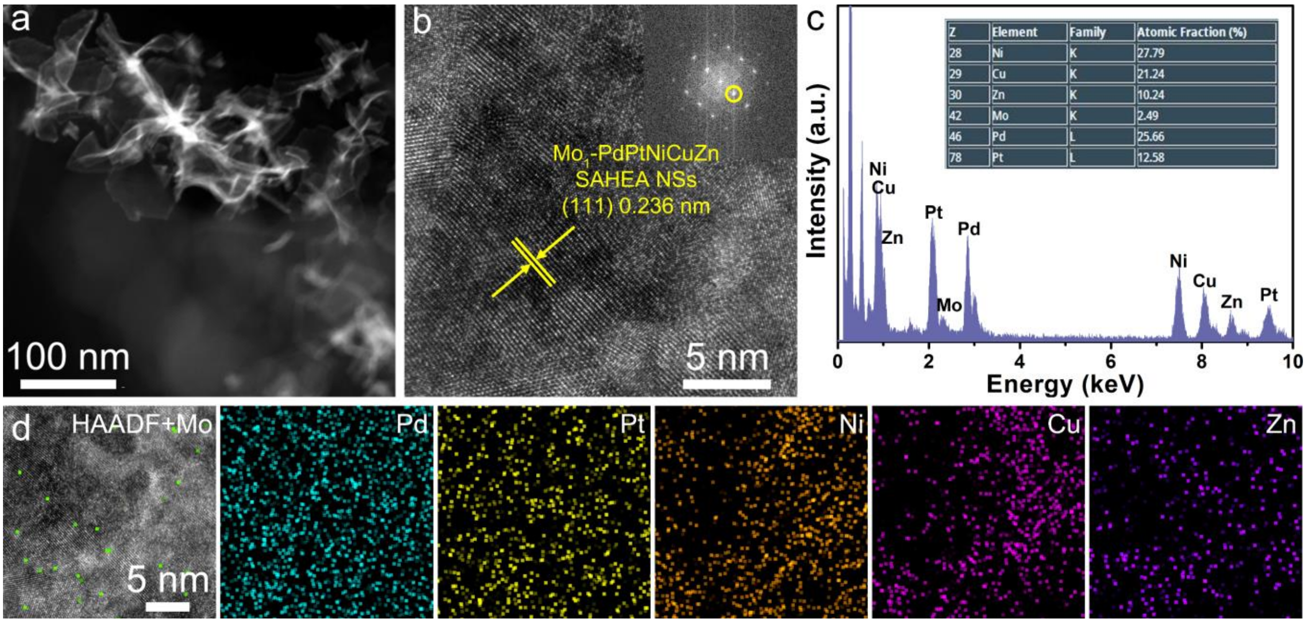

Figure R16. Morphological, structural, and compositional characterizations of Mo_1-PdPtNiCuZn SAHEA nanosheets after MOR stability tests. (a) Representative HAADF-STEM image. (b) High-resolution HAADF-STEM image (*inset* is the FFT pattern). (c) STEM-EDS and atomic ratio of different elements. (d) High-resolution HAADF-STEM-EDS elemental mapping.

6. Z contrast can not show that Mo exists in single atoms since its atomic number is between other elements. Although atomic radius also can't prove single-atom Mo due to the low resolution, the circled atom in fact has a smaller atomic radius and therefore shouldn't be Mo that has a much larger atomic radius.

Response: We sincerely appreciate you for this valuable comment. In order to confirm the presence of single Mo atoms, we have additionally performed the atomic-level electron energy loss spectroscopy (EELS) line-scanning acquisition. Fig. R17b depicts EELS spectra extracted from four adjacent atomic columns, demonstrating the presence of a Mo atom in these atomic columns, with the signature $M_{4,5}$ edge occurring at the expected energy levels [R27]. These features are not present in the EELS of other atomic positions adjacent to this atomic site, further proving that the Mo species in Mo_1-PdPtNiCuZn SAHEA nanosheets mainly exist as single atoms.

We have included the corresponding discussion in the revised manuscript.

"*Atomic-level electron energy loss spectroscopy (EELS) line-scanning acquisition was further performed to confirm that Mo atoms existed as single atoms (Fig. 2c and Supplementary Fig. 10). The spectra demonstrate the presence of a Mo atom in these atomic columns, with the signature* $M_{4,5}$ *edge occurring at the expected energy levels [28]. These features are not present in the EELS of other atomic positions adjacent to this atomic site, proving that the Mo species in* Mo_1*-PdPtNiCuZn*

SAHEA NSs mainly exist as single atoms.”

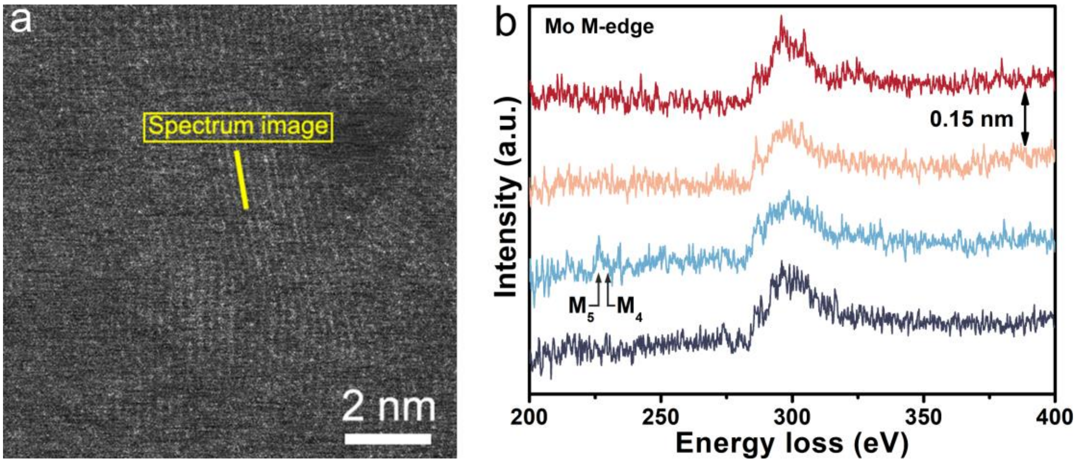

Figure R17. (a) HAADF-STEM image of Mo_1-PdPtNiCuZn SAHEA nanosheets. (b) Background-subtracted EELS line-scanning spectra of four adjacent atomic columns in Mo_1-PdPtNiCuZn SAHEA nanosheets acquired along the line in a (the $M_{4,5}$ edges for Mo are indicated).

Reference:

[R27] Zhu, P. *et al.* Regulating the FeN_4 moiety by constructing Fe-Mo dual-metal atom sites for efficient electrochemical oxygen reduction. *Nano Lett.* **22**, 9507-9515 (2022).

7. Evidence that ~ 1.33 Å can be attributed to Mo-O scattering from MoO_x in SAHEAs.

Response: Thank you so much for your constructive comment. In the Mo K-edge XANES spectra (Fig. 18a), Mo_1-PdPtNiCuZn SAHEA nanosheets exhibit a shoulder structure around 20010 eV in the pre-edge region, which is well-known as a formally dipole forbidden excitation from 1*s* to 4*d* antibonding orbitals directed principally along the Mo=O bond [R28], indicating that the existence of MoO_x with distorted [MoO_6] octahedron structure in Mo_1-PdPtNiCuZn SAHEA nanosheets [R29]. In addition, the least-squares EXAFS curve fit was performed to simulate the quantitative structural parameters of Mo atoms in Mo_1-PdPtNiCuZn SAHEA nanosheets, in which the standard MoO_2 and MoO_3 were chosen to generate the scattering paths for the EXAFS fitting (Fig. R18b). The result reveals that the peak at ~ 1.33 Å in Mo_1-PdPtNiCuZn SAHEA nanosheets agrees well with the Mo-O scattering. The corresponding fitting parameters can be inspected in Table R5. Taken cumulatively, the above results demonstrate the major peak at ~1.33 Å in the FT-EXAFS spectrum of Mo_1-PdPtNiCuZn SAHEA nanosheets can be attributed to Mo-O scattering from MoO_x.

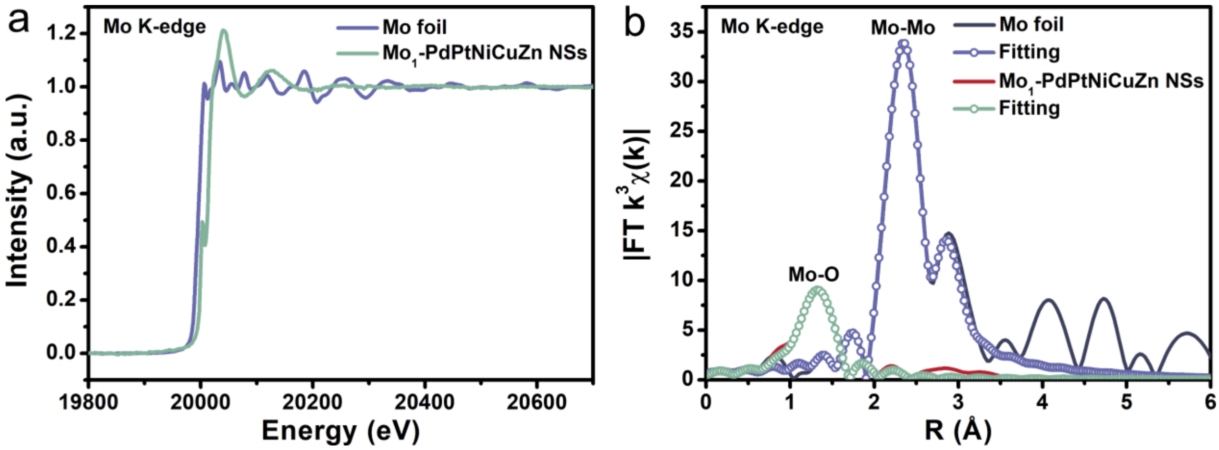

Figure R18. (a) Mo K-edge XANES spectra of Mo_1-PdPtNiCuZn SAHEA nanosheets and Mo foil. (b) k^3-weighted

Fourier transformation of the Mo K-edge EXAFS spectra.

Table R5. Mo K-edge EXAFS fitting parameters for Mo_1-PdPtNiCuZn SAHEA nanosheets.

Sample	Scattering path	*N*	*R*(Å)	σ^2(Å^2)	□ΔE_0(eV)	*R* factor
Mo_1-PdPtNiCuZn	Mo-O1	1.459	1.81	-0.0001	8.338	0.0087
	Mo-O2	1.459	1.71	0.0010		

We have included the corresponding details in the revised manuscript.

"*Notably, the XANES spectrum of Mo_1-PdPtNiCuZn SAHEA NSs indicates a shoulder around 20010 eV in the pre-edge region, indicating that the existence of MoOx with distorted [MoO_6] octahedron structure [29, 30].*"

References:

[R28] Wang, H. *et al. In operando* X-ray absorption fine structure studies of polyoxometalate molecular cluster batteries: Polyoxometalates as electron sponges. *J. Am. Chem. Soc.* **134**, 4918-4924 (2012).
[R29] Yang, Y. *et al.* O-coordinated W-Mo dual-atom catalyst for pH-universal electrocatalytic hydrogen evolution. *Sci. Adv.* **6**, eaba6586 (2020).

8. There are grammar mistakes in the sentence "When Cu(acac)$_2$ and other metal precursors were introduced simultaneously and kept other conditions unchanged".

Response: Thank you so much for pointing out this mistake. We have revised this sentence, and we also carefully checked throughout the manuscript.

"*The Mo_1-PdPtNiCuZn SAHEA nanoparticle assemblies (NPs) can be obtained by the simultaneous introductions of Cu(acac)$_2$ and other metal precursors before the reaction, while other conditions are kept unchanged.*"

9. The following statement cannot be supported by the experimental results "As-obtained NPs exhibit the single-atom Mo-tailored HEA structures and the similar Pd/Pt/Ni/Cu/Zn/Mo compositions to that of SAHEA NSs."

Response: Thank you so much for your valuable comment. We have additionally employed ICP-OES and Fourier transforms of the extended X-ray absorption fine structure (FT-EXAFS) spectroscopy to confirm that Mo_1-PdPtNiCuZn HEA nanoparticles have a similar composition and single-atom Mo-tailored structure to those of Mo_1-PdPtNiCuZn SAHEA nanosheets. The atomic ratio of Pd/Pt/Ni/Cu/Zn/Mo in Mo_1-PdPtNiCuZn SAHEA nanoparticles is determined to be 26.4/12.8/21.7/25.1/11.6/2.4 by ICP-OES, similar to that of Mo_1-PdPtNiCuZn SAHEA nanosheets (26.9/13.3/24.8/21.9/11.0/2.1) (Fig. R19a). In addition, the k^3-weight FT-EXAFS spectra reveal that Mo_1-PdPtNiCuZn SAHEA nanoparticles manifest two characteristic peaks at ~ 1.66 Å and ~ 2.58 Å, attributed to Mo-O and Mo-M (M = Pd/Pt/Ni/Cu/Zn) scatterings, respectively (Fig. R19b). The characteristic peaks of Mo-Mo scattering (~ 2.34 Å for the Mo foil) and Mo-O-Mo scattering (~ 3.19 Å for the MoO_3) are almost absent, proving that the Mo species are immobilized on the Mo-tailored HEA nanoparticles in the form of atomically dispersed sites without long-range coordination to other Mo metal centres. Therefore, we conclude that the obtained Mo_1-PdPtNiCuZn HEA nanoparticles possess similar compositions and single-atom Mo-tailored structures to those of SAHEA nanosheets.

We have included the corresponding details in the revised manuscript and updated the figures in the

revised Supplementary Information.

"*As-obtained NPs exhibit compositions and single-atom Mo-tailored structures similar to SAHEA NSs (Supplementary Fig. 15, 16).*"

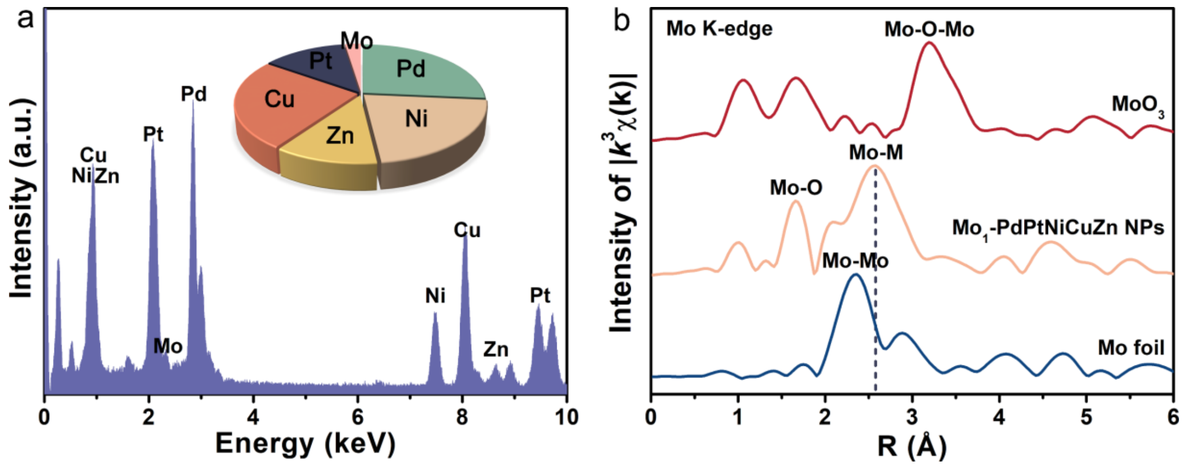

Figure R19. (a) SEM-EDS spectra (*inset* is the corresponding ICP-OES result) of Mo_1-PdPtNiCuZn SAHEA nanoparticles. (b) k^3-weighted Fourier transformation of the Mo K-edge EXAFS spectra.

10. Based on Figure S17, the Zn becomes obviously less after durability tests.

Response: We sincerely appreciate you for your constructive comment. The poor resolution of ordinary TEM-EDS mapping has raised your concern about the Zn content after stability tests. We have additionally employed the aberration-corrected (AC) HAADF-STEM-EDS test with a high resolution to further compare the compositional changes of Mo_1-PdPtNiCuZn SAHEA nanosheets before and after the durability tests. As revealed in Fig. R20, high-resolution HAADF-STEM-EDS elemental mapping reveals that Pd, Pt, Ni, Cu, and Zn elements are uniformly distributed throughout the Mo_1-PdPtNiCuZn SAHEA nanosheets, while Mo atoms are distributed sporadically but evenly doped into the nanosheets in the form of isolated atoms, similar to that before stability tests (Fig. 1k). Furthermore, STEM-EDS show that the composition of Pd/Pt/Ni/Cu/Zn/Mo in Mo_1-PdPtNiCuZn SAHEA nanosheets is 25.7/12.6/27.8/21.2/10.2/2.5, very close to that of before stability tests (26.9/13.3/24.8/21.9/11.0/2.1). These results verify the impressive compositional stability of these tailored Mo_1-PdPtNiCuZn SAHEA nanosheets, arising from the formation of a more stable structure in terms of thermodynamics ($\Delta G_{mix} = \Delta H_{mix} - T\Delta S_{mix}$) and kinetics [R30]. We have included the corresponding details in the revised manuscript and updated the figures in the revised Supplementary Information.

"*The 2D ultrathin nanosheet shape and multimetallic compositions of Mo_1-PdPtNiCuZn SAHEA NSs are maintained, further verifying the impressive compositional and structural stability under the applied potential of 0.77 V (Supplementary Fig. 23).*"

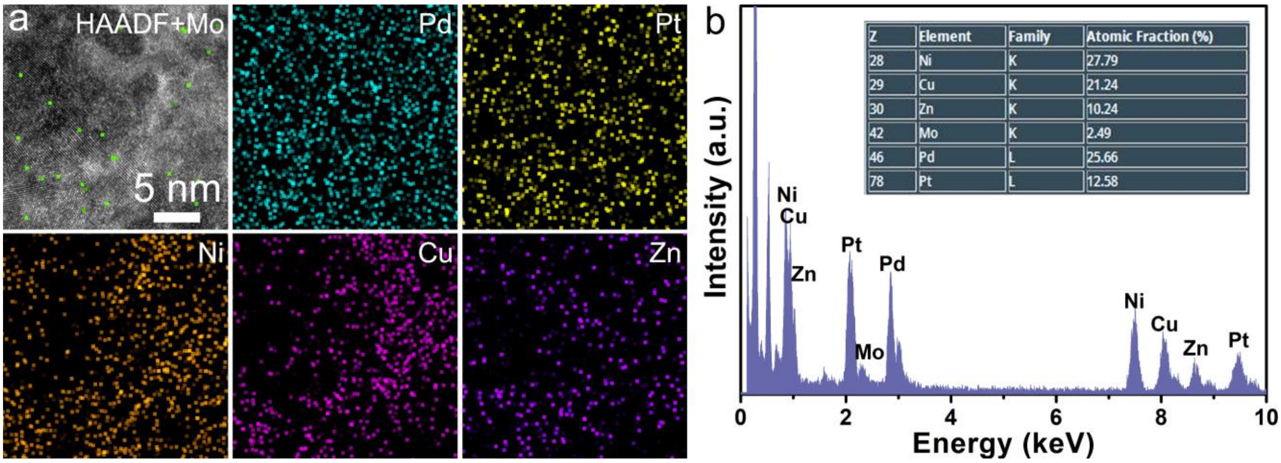

Figure R20. Compositional characterizations of Mo_1-PdPtNiCuZn SAHEA nanosheets after MOR stability tests. (a) High-resolution HAADF-STEM-EDS elemental mapping. (b) STEM-EDS and atomic ratio of different elements.

Reference:

[R30] Glasscott, M.W. *et al*. Electrosynthesis of high-entropy metallic glass nanoparticles for designer, multi-functional electrocatalysis. *Nat. Commun.* **10**, 2650 (2019).

REVIEWERS' COMMENTS

Reviewer #1 (Remarks to the Author):

The authors provided reliable answers to my questions with newly added supplementary materials. All my concerns on this manuscript have been addressed. I recommend accepting the manuscript without further revision.

Reviewer #2 (Remarks to the Author):

The quality of the manuscript has been greatly improved through fully revising the manuscript and adding new analyses. I recommend its acceptance to our journal and the following minor issues may be considered during further revision.

1. In the present work, the author selected PdPtNiCuZn as a high-entropy-alloy electrocatalyst to synthesize and research. What roles do the elements in such alloy play? Why choose such element combination? Are there any additional advantages through combining these elements?

2. For simulation calculation, the arrangement of MoPdPtNiCuZn is irregular. Any particular reason to choose such arrangement? How can one be sure that this model is suitable for the real sample structure? What is the influence of different arrangement on its activity toward methanol oxidation?

Point-by-point response to the reviewers' comments

To Reviewer 1:

The authors provided reliable answers to my questions with newly added supplementary materials. All my concerns on this manuscript have been addressed. I recommend accepting the manuscript without further revision.

Response: Many thanks for this kind comment. We are delighted to know that you were satisfied with the revisions.

To Reviewer 2:

The quality of the manuscript has been greatly improved through fully revising the manuscript and adding new analyses. I recommend its acceptance to our journal and the following minor issues may be considered during further revision.

Response: Thank you so much for the positive assessment of our revisions and additional professional comments to improve our manuscript.

1. In the present work, the author selected PdPtNiCuZn as a high-entropy-alloy electrocatalyst to synthesize and research. What roles do the elements in such alloy play? Why choose such element combination? Are there any additional advantages through combining these elements?

Response: Thank you so much for your valuable comments. It is well known that Pt/Pd-based materials are the most intensively studied MOR electrocatalysts with low overpotentials for initiating reactions and superior catalytic performance. However, their high dosage and poor tolerance to CO are inevitable barriers to large-scale commercial applications. Alloying Pt/Pd with one or more functional components, such as Mo, Ni, Co, and Cu, is one of the most conventional and effective strategies to enhance the anti-poisoning effect of the catalyst and improve its performance. Herein, we synthesize a class of single-atom Mo-tailored PdPtNiCuZn high-entropy-alloy nanosheets by rational selection of element configuration and composition as efficient electrocatalysts for enhancing the methanol oxidation reaction catalysis by promoting the formate pathway.

• Among them, the role of each metal element is summarized as follows: (1) Pt and Pd work as the main active metals for efficient adsorption of methanol and C-H/O-H activation. Of note, Pd is also the key metal in the fabrication of 2D ultrathin nanosheets. (2) Theoretical calculations and spectroscopic results reveal that Ni and Mo elements serve as electron depletion centers during the MOR process, meaning efficient electron transfer. (3) Meanwhile, Cu and Zn elements not only lower the energy barrier of electron transfer during oxidation, but also promote the stability of MOR intermediates (Fig. R1). (4) Ni, Cu, and Zn elements further dilute the Pt-Pt ensembles in Mo_1-PdPtNiCuZn SAHEA nanosheets and provide isolated Pt sites, leading to the reduction and even the elimination of CO adsorption and steering the MOR towards the formate-dominated pathway. (5) Oxophilic Mo single atoms as promoters modify the electronic structure of isolated Pt sites in the HEA host, optimizing the adsorption behavior of intermediates to achieve a more energetically favorable pathway and minimize the MOR barrier.

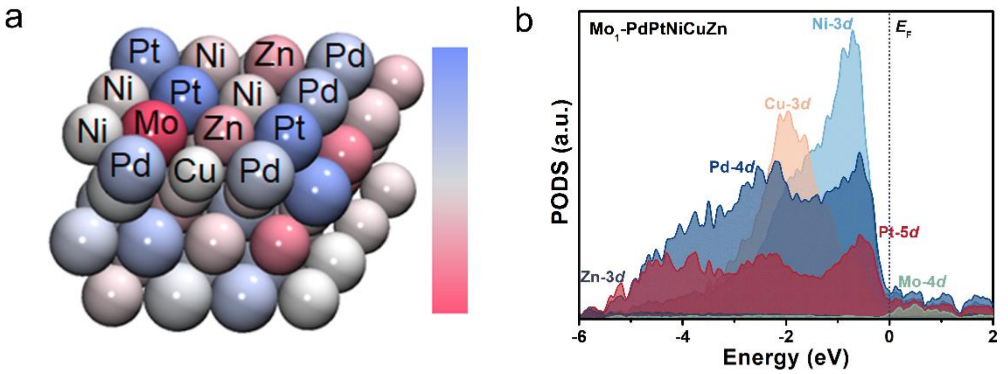

Figure R1. DFT calculations for the structural configuration and energetic reaction pathways. a The Bader charge simulation for Mo_1-PtPdFeCoNi. b The PDOSs of Mo_1-PdPtNiCuZn.

• Because of the synergy and electronegativity differences between each metal, the surface charge of HEAs will redistribute, optimizing their electronic structure. As depicted in Fig. R2, comparing with the electronic structure of Pt/C reveals that the overall *d*-band center of Mo_1-PdPtNiCuZn has significantly shifted upward, suggesting that the improved electron transfer efficiency of the electrocatalysts. Due to the reduced occupancy of the anti-bonding orbitals, the upward shift of the *d*-band center can contribute to the strong adsorption of intermediates. Therefore, Mo_1-PdPtNiCuZn with the up-shifted *d*-band center displays the stronger binding energy with key reaction intermediates and *OH, thus increasing the chance of these intermediates being further oxidized to CO_2. Combining experimental and theoretical research, such element combination can provide the optimal surface configuration, suitable electronic microenvironment, and ideal adsorption/desorption sites for MOR, enabling proper adsorption of key intermediates to enhance the formate-dominated MOR. In addition, given the high abundance, similar atomic radius, and lower heat of formation of Ni, Cu, and Zn, they can enhance the metal dispersion and alloying and easily form a more stable Pt/Pd-based HEA structure in terms of thermodynamics.

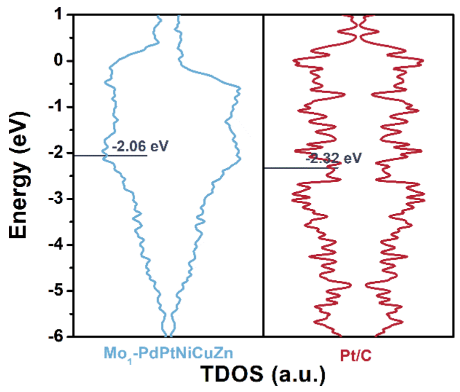

Figure R2. The TDOS of *d*-band in Mo_1-PdPtNiCuZn and Pt/C models.

• It is found that the surface transition metal may weaken the binding of the chemical species to the Pt surface layer, resulting in a catalytic surface with high ORR activity. Furthermore, Pt is used as the interaction site of *H, and oxyphilic metals, such as Ni, Mo, Co, *etc.*, with good *OH interaction sites to promote water dissociation. Given this, the single-atom Mo-tailored PdPtNiCuZn high-entropy-alloy catalyst allows for the formation of tailorable active sites in multiple elements adjacent to each other by modifying composition, and these active centers exhibit extensive properties, which can be adapted to any desired response. Therefore, applications are not necessarily limited to MOR.

We have included the corresponding details in the revised manuscript.

"*As depicted in Fig. 6b, c, the Ni and Mo elements serve as electron depletion centers during the MOR process, promoting electron transfer for the HEA surfaces. Meanwhile, the efficient d-d orbital coupling between Pt, Pd, Cu, and Zn not only lowers the energy barrier of electron transfer during*

oxidation but also can stabilize the valence state of Pt sites, facilitating the stabilization of intermediates for MOR[19]. Such element combination can provide the optimal surface configuration, suitable electronic microenvironment, and ideal adsorption/desorption sites for MOR, enabling proper adsorption of key intermediates to enhance the formate-dominated MOR. Compared with the PdPtNiCuZn, introducing Mo single atoms near the Pt sites further enhances the electroactivity."

2. For simulation calculation, the arrangement of MoPdPtNiCuZn is irregular. Any particular reason to choose such arrangement? How can one be sure that this model is suitable for the real sample structure? What is the influence of different arrangement on its activity toward methanol oxidation?

Response: Thank you so much for your professional comments. In this study, the as-made Mo_1-PdPtNiCuZn high entropy alloy has a highly disordered solid solution structure, with various atoms arranged in its lattice in a disordered and scattered manner. Considering the theoretical possibility of multiple elements occupying the same position in the original cell, we use the mcsqs method in the Density Functional Theory (DFT) Alloy Theoretic Automated Toolkit (ATAT) software package for simulation modeling of the Mo_1-PdPtNiCuZn high entropy alloy. We create an atomic arrangement such that for a given component, the various atoms in the alloy all appear at different lattice points with a specific probability distribution. The randomness of the atomic arrangements obtained is close to an ideal random state. We optimized the shape of the supercell while also optimizing the atomic position, thus ensuring a detailed spatial configuration without the influence of preset supercell shapes. This highly random structure can well reflect the changes in the local environment around each atom in the lattice, which is beneficial for subsequent research on the methanol oxidation performance of Mo_1-PdPtNiCuZn high entropy alloy.

• Several factors were considered to ensure that the constructed Mo_1-PdPtNiCuZn model is suitable for real samples. Firstly, the information on crystal structure types, atomic ratios of various elements and alloy compositions, and dominant crystal planes of Mo_1-PdPtNiCuZn high-entropy alloy are obtained by XRD, HAADF-STEM, ICP-OES, and other structural analyses to carry out the theoretical model building. After obtaining the above information, we chose an appropriate size of the supercell, which was usually large enough to accommodate various atoms and produce the desired disorder. Various atoms were randomly placed in the supercell to generate an initial idealized random structure. Energy calculations were performed on it to study the stability and other properties of the structure. Finally, in order to maintain the consistency of the electronic structure between the theoretical model and the actual material, we analyzed and compared the simulated electronic structures (*e.g.*, charge density differential, density of states) with experimentally obtained XPS data to verify the applicability of the model.

• The number of active sites exposed on the surface varies when the atoms are arranged differently, affecting the adsorption capacity of the catalyst for methanol molecules. Moreover, different arrangements may lead to changes in the electronic structure of the catalyst, affecting the stability of the reaction intermediates and the adsorption capacity of species on the catalyst surface. Different atomic arrangements may also lead to different reaction pathways and activation energies, affecting the rate and efficiency of methanol oxidation. In this study, we constructed a model with an atomic arrangement closer to the actual sample to investigate the MOR mechanism on the surfaces.

We have included the corresponding details in the revised Supplementary Information.

"We constructed the corresponding computation model based on the experimentally obtained structural and component information of Mo_1-PdPtNiCuZn. Specifically, we selected an appropriate supercell with a sufficient size capable of accommodating a wide variety of atoms and producing the desired disorder. Different atoms were randomly placed in the supercell to generate an initial

idealized random structure. Energy calculations were performed to find the most stable structure of Mo_1-PdPtNiCuZn and subsequently to investigate the mechanism of methanol oxidation reaction on its surface."

In the end, we would like to express our gratitude to the reviewers and the editor for their precious time. We sincerely appreciate all reviewers who are satisfied with this revision and recommend acceptance of the paper for publication in ***Nature Communications***.
